# Overcoming resistance in lung cancer: combined mitochondrial destabilization by lipophilic cations and doxycycline in hypoxic non-small cell lung carcinoma

**DOI:** 10.3389/fphar.2026.1797291

**Published:** 2026-05-04

**Authors:** Mabel Catalán, Denny Vidal, Alfredo Molina-Berríos, Javiera Carrasco-Rojas, Rodrigo López-Muñoz, Gisella Vásquez, Ivonne Olmedo, José A. Jara

**Affiliations:** 1 Instituto de Ciencias Biomédicas (ICBM), Facultad de Medicina, Universidad de Chile, Santiago, Chile; 2 Instituto de Investigación en Ciencias Odontológicas (ICOD), Facultad de Odontología, Universidad de Chile, Santiago, Chile; 3 Instituto de Farmacología y Morfofisiología, Facultad de Ciencias Veterinarias, Universidad Austral de Chile, Valdivia, Chile

**Keywords:** cancer treatment, doxycycline, drug combination, lipophilic cations, non-small cell lung carcinoma, targeting mitochondria

## Abstract

**Introduction:**

Lung cancer remains the leading cause of cancer-related mortality worldwide. Drug resistance is a major limitation of current therapies, prompting the search for new treatment strategies. Lung tumors frequently develop a hypoxic microenvironment associated with aggressive behavior and unfavorable clinical outcomes. Tumor-initiating cells (TICs), also known as cancer stem cells, and hypoxia-driven metabolic adaptations contribute significantly to therapy resistance. Mitochondrial destabilization has emerged as a promising invariant target in TICs. Triphenylphosphonium (TPP^+^)-conjugated hydroxybenzoates selectively accumulate in the mitochondrial matrix, driven by membrane potential, disrupting organelle function. Additionally, doxycycline inhibits mitochondrial biogenesis and reduces mitochondrial mass. Here, we evaluate a therapeutic strategy combining TPP^+^-conjugated lipophilic cations with doxycycline to target mitochondrial vulnerability in non-small cell lung cancer.

**Methods:**

TPP^+^ lipophilic cations conjugated to benzoate derivatives, alone or combined with doxycycline, were evaluated for their ability to disrupt mitochondrial function, reduce cell viability, and induce apoptosis in two lung cancer cell lines under normoxic and hypoxic conditions.

**Results:**

Our results demonstrate that these compounds exhibit cytotoxicity in lung cancer cells, particularly under hypoxic conditions, consistent with mitochondrial functional impairment. Combinations of TPP^+^C_10_/doxycycline and GA-TPP^+^C_10_/doxycycline exhibited synergistic cytotoxicity in both normoxia and hypoxia, and increased apoptotic cell death compared to monotherapies.

**Conclusion:**

Targeting mitochondrial functions using mitochondria-directed compounds, particularly in combination with doxycycline, represents a promising therapeutic approach for lung cancer. This strategy may be especially effective in hypoxic microenvironments, where conventional therapies often fail. Further *in vivo* validation is warranted to support the translational potential of this approach.

## Graphical Abstract

**Figure:**
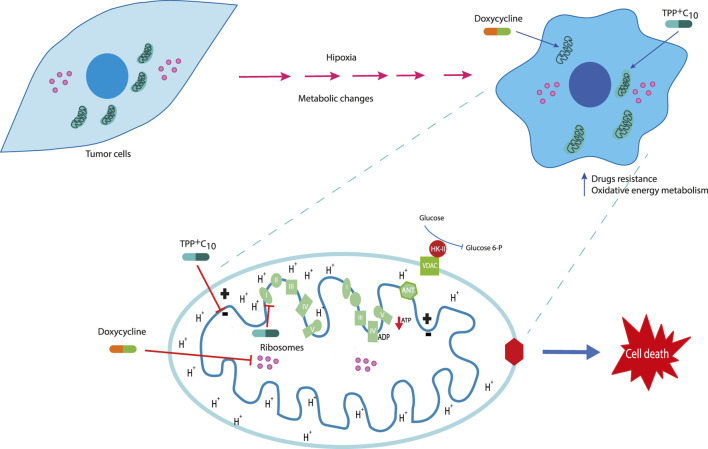
Graphical abstract TPP + C10 accumulates in the mitochondrial matrix of lung cancer cells, where it disrupts mitochondrial function, alters oxidative metabolism, and induces metabolic stress. This triggers a compensatory increase in mitochondrial biogenesis and contributes to therapy resistance, particularly under hypoxic conditions. Doxycycline inhibits mitochondrial protein synthesis and biogenesis by targeting mitochondrial ribosomes. Combined treatment with TPP + C10 and doxycycline prevents adaptive mitochondrial renewal, leading to impaired energy production and enhanced tumor cell death.

## 1 Introduction

Lung cancer remained the leading cause of cancer-related mortality worldwide, with an estimated 1.8 million deaths annually, accounting for approximately 18% of all cancer deaths (Meyer et al., 2024). Lung cancer is histologically classified into small-cell lung cancer, which represents approximately 13% of the cases, and non-small cell lung cancer (NSCLC), accounting for about 85% of cases (Su et al., 2025). For early-stage NSCLC, surgical resection remains the primary treatment modality. However, in locally advanced and metastatic disease, cytotoxic chemotherapy continues to be a central component of the treatment for most patients. In recent years, targeted therapies, particularly immune checkpoint inhibitors targeting the Programmed Death-1 (PD-1)/Programmed Death Ligand-1 (PD-L1) axis, have revolutionized NSCLC treatment by providing durable responses in a subset of patients. Nevertheless, fewer than 50% of patients derive significant clinical benefits, underscoring the urgent need to develop and expand alternative therapeutic strategies (Roussot et al., 2025).

Lung cancer has a poor prognosis, with a 5 years survival rate ranging from 5%–15%. In the early 2000s, the median overall survival of patients with recurrent or metastatic NSCLC was approximately 14–16 months with platinum-based chemotherapy. Although new therapeutic approaches have since emerged, clinical resistance remains as major obstacle in effective cancer treatment (Huang et al., 2025). Key contributors to therapy resistance include the presence of tumor-initiating cells (TICs), also referred to as cancer stem cells (CSCs), as well as microenvironmental factors such as hypoxia (Yokomichi et al., 2014).

Hypoxia is a hallmark of the lung cancer microenvironment and a major determinant of clinical outcomes, as it promotes genetic instability, tumor metastasis, and invasiveness (Chen et al., 2023). Under hypoxic conditions, hypoxia-inducible factor 1α (HIF-1α) accumulates in the nucleus and binds to target genes such as BNIP3, PGK1, HK1, and TP11(Jun et al., 2017). These genes regulate proliferation, apoptosis, metabolism, invasion, and resistance to cancer therapy. Increasing evidence indicates that HIFs play a central role in regulating CSCs population. Activation of HIF-1α not only increases the number of CSCs but also enhances their stemness or stem-like phenotype (Garziera et al., 2017). Additionally, the CSCs hypothesis suggests that tumors are arranged in a hierarchical structure, with a small subset of stem-like cells that are responsible for tumor initiation and growth. This population of CSCs has several key properties, including the ability to divide and differentiate into multiple lineages, self-renew, and exhibit higher intrinsic resistance to conventional therapies (Romeo and Arcos, 2023).

For over a decade, mitochondrial destabilization has been recognized as an invariant target in CSCs and a promising anti-cancer strategy (Fulda et al., 2010; Catalán et al., 2020). Numerous studies have reported the development of *mitocans*, compounds that target cancer cell mitochondria and exhibit cytotoxic and/or antitumor activity across various cancer types. Many of these agents are hydrophobic molecules that associate with multiple subcellular structures, which can limit their cellular uptake and specificity.

Following the strategy pioneered by Murphy and Smith (Kelso et al., 2001; Smith et al., 2003), conjugation of bioactive molecules to the cationic triphenylphosphonium (TPP^+^) moiety enhances mitochondrial accumulation and biological activity. The rationale for targeting mitochondria with TPP^+^-conjugated decyl mono- or polyhydroxybenzoates relies on the delocalization of the positive charge across the three flanking phenyl groups, which allows these compounds to accumulate preferentially in negatively charged environments such as mitochondria (Jara et al., 2014; Sandoval-Acuña et al., 2016). The plasma membrane potential is approximately −60 mV, whereas the mitochondrial transmembrane potential (ΔΨm) reaches about −180 mV. According to the Nernst equation, this potential difference drives a ∼10-fold accumulation of TPP^+^-conjugated compounds in the cytoplasm and ∼1,000-fold accumulation within mitochondria (Kelso et al., 2001). Thus, TPP^+^ conjugation overcomes the limitations of conventional lipophilic delocalized cations by promoting selective mitochondrial localization. However, mitochondrial dysfunction alone may not be sufficient for durable anticancer efficacy, as it can activate drug resistance mechanisms such as autophagy and mitophagy (Basit et al., 2017). Accordingly, TPP^+^C_10_, a gallic acid derivative with a 10-carbon aliphatic linker, was identified for our group as a potent mitochondria-targeted cytotoxic agent on cancer cell lines. In addition, TPP^+^ was also linked to a series of decyl mono- and dihydroxybenzoate derivatives to investigate structure–activity relationships related to benzoic acid substituents (Frey et al., 2007; Jara et al., 2014; Sandoval-Acuña et al., 2016)

On the other hand, doxycycline is a broad-spectrum bacteriostatic antibiotic that inhibits bacterial protein synthesis by binding to the 30S ribosomal subunit. Beyond its antimicrobial activity, doxycycline has been reported to reduce the viability and proliferation of breast cancer cells and breast cancer stem cells, primarily through inhibition of mitochondrial biogenesis via interference with the 28S ribosomal subunit and aldehyde dehydrogenase activity, leading to loss of mitochondrial mass (Lin et al., 2018; Zhang et al., 2017). Doxycycline also induces apoptosis and suppresses the proliferation and invasion of cancer stem cells derived from cervical and lung carcinomas (Yang et al., 2015; Dijk et al., 2020). Moreover, combined treatment with doxycycline, azithromycin, and vitamin C has been shown to impair mitochondrial function and eradicate cancer stem cells (Fiorillo et al., 2019).

Currently, no approved therapies for NSCLC specifically target hypoxic tumor cells or CSCs populations. Given that these subpopulations are highly dependent on mitochondrial function, particularly oxidative phosphorylation, a combination strategy employing lipophilic cations to disrupt mitochondrial function together with doxycycline to inhibit mitochondrial biogenesis may represent a promising therapeutic alternative. This approach differs from most current treatment strategies, which largely rely on immunotherapy using monoclonal antibodies targeting diverse immune checkpoints. Therefore, in this study, we will evaluate the effects of previously synthesized lipophilic cations derived from gallic (TPP^+^C_10_), gentisic (GA-TPP^+^C_10_), protocatechuic (PA-TPP^+^C_10_), or salicylic acids (SA-TPP^+^C_10_), in combination with doxycycline in NSCLC cells under hypoxic conditions.

## 2 Materials and methods

### 2.1 Materials

Roswell Park Memorial Institute RPMI 1640 culture medium (code R6504 Sigma-Aldrich®), Eagle`s Minimal Essential Medium MEM (code M0643 Sigma-Aldrich®), Amphotericin B (#03-028-1B, Biological Industries®), Fetal Bovine Serum (FBS, #04-027-1A Biological Industries®), Penicillin-Streptomycin (#03-031-1B Biological Industries®), Fetal Bovine Serum (FBS, #04-127-1A Biological Industries®), Sodium Bicarbonate (#S5761 Sigma-Aldrich®), dimethyl sulfoxide or DMSO (#276855 Sigma-Aldrich®), 10X phosphate buffered saline pH 7.4 (PBS) (#16505 Merck®), Trypsin-EDTA 0.25% (#03-050-1B Biological Industries®), 3-(4,5-dimethylthiazol-2-yl)-2,5- diphenyltetrazolium or MTT (#M6494 Thermo Fisher Scientific®), Doxycycline (D9891 Sigma-Aldrich®), CellTiter-Glo® Luminescent Cell Viability Assay Kit, tetramethylrhodamine methyl ester or TMRM (#ab275547, Abcam®), Albumin Fraction V (BSA, #A1391 PanReac®).

### 2.2 Synthesis of benzoate-TPP + derivatives

Benzoate-TPP^+^ derivatives were synthesized and characterized as previously described (Sandoval-Acuña et al., 2016). In detail, the first step involved the synthesis of (10-hydroxydecyl) triphenylphosphonium bromide, performed as follows: a solution of 10-bromodecan-1-ol in dry acetonitrile (100 mL) was treated with triphenylphosphine. The solution was refluxed with stirring for 48 h. The solvent was removed under vacuum, and the crude product was subjected to chromatography on silica gel (EtOAc, MeOH) to yield (10-hydroxydecyl) triphenylphosphonium bromide as a colorless oil (63%). ^1^H NMR (400 MHz, DMSO-d6): δ 1.01–1.51 (m, 18H, CH2), 3.57 (t, J = 7.1 Hz, 2H, CH2), 7.73–7.91 (m, 15H, ArH). HRMS: m/z 485.4312 (calc for C_27_H_33_BrOP 485.4357). The second step involved a Steglich esterification, utilizing N, N-dicyclohexylcarbodiimide (DCC) as the reagent, 4-dimethylaminopyridine (DMAP) as the catalyst, and N, N-dimethylformamide (DMF) as the solvent. Briefly, in the atmosphere of N_2_, a solution of each respective hydroxybenzoic acid (3,4,5-trihydroxybenzoic acid, 2-hydroxybenzoic acid, 2,5-dihydroxybenzoic acid, or 2,3-dihydroxybenzoic acid) in dry DMF was treated with a solution of DCC in dry DMF. The mixture was cooled to 0 °C, and a solution of (10-hydroxydecyl) triphenylphosphonium bromide and DMAP in DMF were added. The reaction was stopped the next day, and any resulting precipitate was removed by filtering. The solvent was then removed, producing a residue that was subjected to chromatography on silica gel (DCM, MeOH). On the other hand, as doxycycline is a commercial drug, this was purchased from Sigma-Aldrich®. All drug stock solutions and dilutions were prepared in DMSO.

### 2.3 Cell lines

NCI-H727 and NCI-H1299 were acquired from ATCC. NCI-H727 represents a well-differentiated pulmonary neuroendocrine tumor model (Ear et al., 2024), whereas NCI-H1299 is an aggressive NSCLC line lacking functional p53 (Todorova et al., 2021. Lung fibroblasts served as non-tumoral controls to assess treatment selectivity. Lung fibroblast (non-tumor cells) was acquired in the Chilean Institute of Public Health.

### 2.4 Normoxic cell cultures

NCI-H1299 and NCI-H727 cells were cultured in T25 flasks containing RPMI-1640 medium supplemented with 10% heat-inactivated fetal bovine serum (FBS), 2% penicillin/streptomycin, and 1% amphotericin B, and maintained at 37 °C in a humidified atmosphere with 5% CO_2_ and 21% O_2_. The lung fibroblast cell line was cultured in Petri dishes or T75 flasks using DMEM High Glucose supplemented with 10% FBS, 1% penicillin/streptomycin, and 1% amphotericin B, under the same temperature and atmospheric conditions.

### 2.5 Hypoxic cell cultures

NCI-H1299 and NCI-H727 cells were cultured in T25 flasks with RPMI-1640 medium supplemented with 10% FBS, 2% penicillin/streptomycin, and 1% amphotericin B. Hypoxic conditions were established using a hypoxia chamber. Nitrogen gas (N_2_) was introduced into the chamber for 14 s at a flow rate of 30 L/min, reducing the oxygen concentration to 5% O_2_. Oxygen levels were verified using an oxygen meter and maintained for 24 h prior to experimental procedures. The hypoxia chamber was kept at 37 °C, as previously described (Alva et al., 2022).

### 2.6 Cell viability assay

Cells were maintained under normoxic or hypoxic conditions for 24 h prior to treatment. Subsequently, cells were seeded into 96-well plates at a density of 2 × 10^4^ cells per well. After 24 h of attachment, benzoate–TPP^+^ compounds were added at increasing concentrations and incubated for 24, 48, or 72 h. At the end of each treatment period, cells were washed twice with 1x PBS, and 100 μL of MTT solution (0.5 mg/mL) was added to each well. Following a 2-h incubation at 37 °C, the MTT solution was removed, and the resulting formazan crystals were dissolved in 40 μL of DMSO. Absorbance was measured at 570 nm using a microplate reader (Infinite F50®, Tecan Group Ltd., Switzerland). Cell viability was analyzed using GraphPad Prism 9 software, and results are expressed as IC_50_ values (half-maximal inhibitory concentration) calculated from sigmoidal concentration–response curves with variable slope, as previously described (Catalán et al., 2021).

### 2.7 Drug combination assays

After determining the IC_50_ values of the individual compounds, the effects of drug combinations were evaluated in NCI-H727 and NCI-H1299 cell lines. Serial dilutions were prepared for (a) TPP^+^C_10_ (1–15 µM), (b) GA-TPP^+^C_10_ (1–10 µM), and (c) doxycycline (5–50 µM). Cells from normoxic or hypoxic conditions were seeded into 96-well flat-bottom plates at a density of 2 × 10^4^ cells per well and incubated for 24 h at 37 °C in a humidified atmosphere with 5% CO_2_. Subsequently, one compound was added along the horizontal axis and the second compound along the vertical axis of the plate, both in increasing concentrations, to generate the drug combination matrix. Cells were then incubated for an additional 72 h. At the end of the treatment period, the medium containing the compounds was discarded, and wells were washed twice with sterile 1x PBS to remove residual drugs. MTT solution was added and incubated for 4 h. The supernatant was then removed, and the resulting formazan crystals were solubilized in DMSO. Absorbance was measured at 570 nm using a microplate reader. Cell viability percentages were calculated, and combination effects (synergistic, additive, or antagonistic) were analyzed using Combenefit software (version 2.021).

### 2.8 Mitochondrial transmembrane potential (ΔΨ)

Changes in mitochondrial transmembrane potential (ΔΨm) were assessed using tetramethylrhodamine methyl ester (TMRM) as a fluorescent probe. Cells maintained under normoxic or hypoxic conditions were seeded into 24-well plates at a density of 1 × 10^5^ cells per well and allowed to attach for 24 h. Cells were then loaded with 200 nM TMRM and immediately exposed to increasing concentrations of each compound (5–50 μM) for 30 min at 37 °C. After treatment, cells were washed with PBS 1x, detached, and resuspended in cold PBS 1x for flow cytometric analysis using a FACSAria® III cytometer (BD Biosciences). TMRM fluorescence was detected at 540 nm (excitation) and 595 nm (emission), as previously described (Catalán et al., 2021).

### 2.9 Intracellular ATP levels

Intracellular ATP levels were quantified using the CellTiter-Glo® Luminescent Assay (Promega, Madison, WI) according to the manufacturer’s instructions. Briefly, 1 × 10^4^ cells per well were seeded into 96-well plates and cultured for 24 h. Cells were then treated with the compounds at increasing concentrations for 4 h. Subsequently, 100 µL of cell suspension was transferred to opaque 96-well plates and incubated at room temperature in the dark for 10 min. Luminescence was measured using a Varioskan Flash spectral scanning reader (Thermo Scientific) (Jara et al., 2020).

### 2.10 Oxygen consumption assay

Cellular respiration was measured using a polarographic method to assess oxygen consumption rates (OCR) in tumor cells cultured under normoxic and hypoxic conditions. Cells were trypsinized and counted as previously described (Frey et al., 2007). Subsequently, 0.6 mL of cell suspension in 1x PBS (pH 7.4) at 25 °C was added to the electrode chamber containing 8.3 mM of L-glutamine. Oxidative phosphorylation was first inhibited by the addition of oligomycin (2.5 μg/mL), and maximal uncoupled respiration was then determined by adding the protonophore CCCP (0.133 µM). Thereafter, the effects of the test compounds on OCR were evaluated by adding increasing concentrations of each compound (70, 140, and 280 µM). Results were compared with vehicle-treated controls (DMSO).

### 2.11 Apoptosis analysis by flow cytometry

Apoptosis was evaluated by flow cytometry using Annexin V and propidium iodide (PI) staining. NCI-H727 cells were seeded and treated with doxycycline, TPP^+^C_10_, GA-TPP^+^C_10_, or their combinations at the indicated concentrations under normoxic or hypoxic conditions. After treatment, cells were harvested, washed with cold PBS 1x, and resuspended in binding buffer. Cells were then incubated with fluorochrome-conjugated Annexin V and PI, protected from light, according to the manufacturer’s instructions. Samples were analyzed using a flow cytometer, and at least 10,000 events per sample were recorded. Data were processed using FlowJo software to quantify viable (Annexin V^−^/PI^−^), early apoptotic (Annexin V^+^/PI^−^), late apoptotic (Annexin V^+^/PI^+^), and necrotic (Annexin V^−^/PI^+^) cell populations. The percentage of total apoptotic cells was calculated.

### 2.12 Mitochondrial mass analysis by MitoTracker green staining

Mitochondrial mass was assessed using MitoTracker™ Green FM, a fluorescent probe that accumulates in mitochondria independently of membrane potential. NCI-H727 cells were treated with TPP^+^C_10_, GA-TPP^+^C_10_, doxycycline, or their combinations under normoxic or hypoxic conditions for the indicated time points. After treatment, cells were incubated with MitoTracker Green (100 nM) in complete culture medium at 37 °C for 30 min, protected from light. Cells were then washed with phosphate-buffered saline, harvested, and analyzed by flow cytometry. At least 10,000 events per sample were acquired, and mean fluorescence intensity was quantified using FlowJo software as an index of mitochondrial mass. Data were normalized to untreated controls.

### 2.13 Statistical analysis

The data were analyzed using one-way or two-way ANOVA, followed by Tukey’s *post hoc* test. The IC_50_ was calculated using a dose-response curve with a variable slope in GraphPad Prism 9. The differences were considered significant at p < 0.05. The analysis of synergy and selectivity index was performed through Combenefit 2.021 software using Loewe´s combination models.

## 3 Results

### 3.1 Effect on cell viability

**FIGURE 1:**
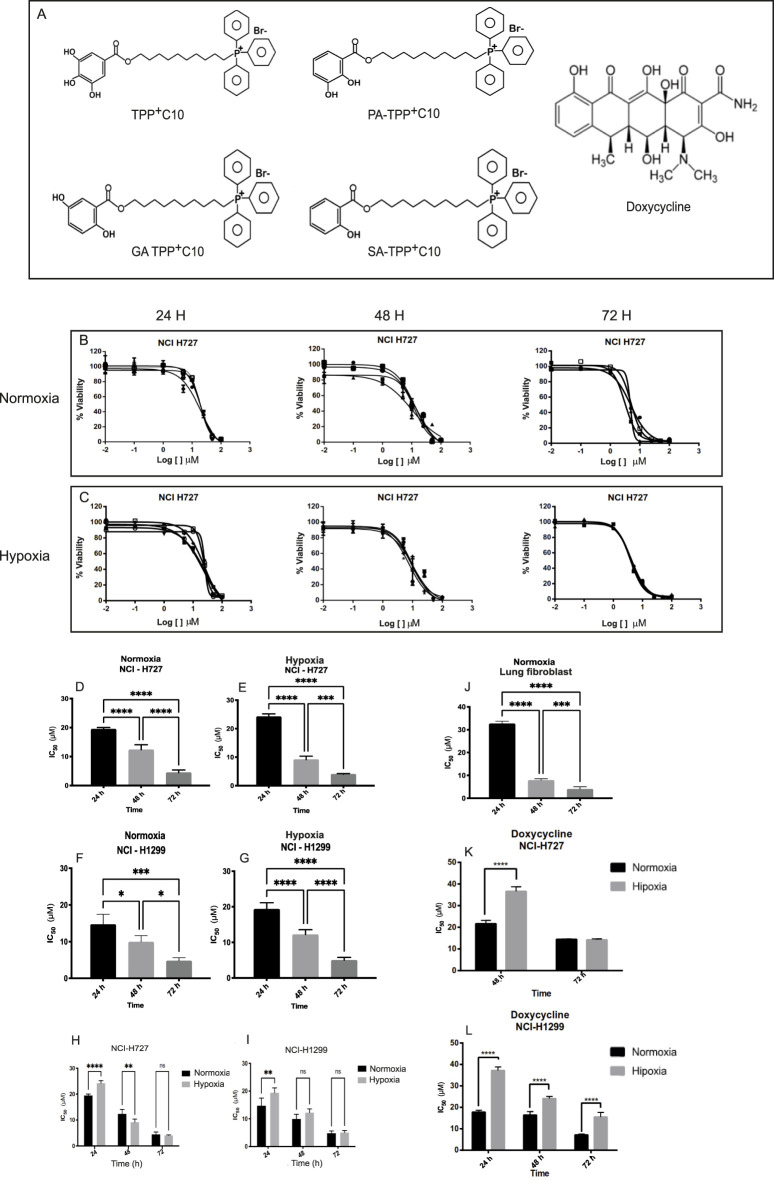
Cytotoxic effect of TPP^+^C_10_ on NSCLC. **(A)** chemical structures of TPP^+^C_10_ (Triphenyl(10-((3,4,5-trihydroxybenzoyl)oxy)-decyl)phosphonium bromide; GA-TPP^+^C_10_ (Triphenyl(10-((2,5-dihydroxybenzoyl)oxy)-decyl)phosphonium bromide); SA-TPP^+^C_10_ (Triphenyl(10-((2-hydroxybenzoyl)oxy)-decyl)phosphonium bromide); and PA-TPP^+^C_10_ Triphenyl(10-((2,3-dihydroxybenzoyl)oxy)-decyl)phosphonium bromide. **(B)** Semi-logarithmic curves depict the concentration-dependent cytotoxic effect of TPP^+^C_10_ at three time points in normoxic NCI-H727 cells. For each time point, data from independent experiments are shown. The corresponding results for the remaining lipophilic cations are presented in Supplementary Figures S1–S6, and those for doxycycline in Supplementary Figures S7, S8. **(C)** Semi-logarithmic curves showing the concentration-dependent cytotoxic effect of TPP^+^C_10_ at three time points in hypoxic NCI-H727 cells **(D–G)** Time-dependent changes in IC_50_ values for NCI-H727 and NCI-H1299 cell lines under normoxic and hypoxic conditions. **(H–I)** Comparison of IC_50_ values between normoxic and hypoxic conditions over time in NCI-H727 **(H)** and NCI-H1299 **(I)** cells. **(J)** Comparison of IC_50_ values between on lung. Fibroblast at different times. **(K–L)** Direct comparison of IC_50_ values between normoxic and hypoxic cultures over time in NCI-H727 **(K)** and NCI-H1299 **(L)** cell lines. Data represent results from at least 3 independent experiments. Statistical significance: *p < 0.05; ***p < 0.001; ****p < 0.0001.

Figure 1A shows the chemical structures of the mitochondria-targeted lipophilic cations derived from gallic (TPP^+^-C_10_), gentisic (GA-TPP^+^-C_10_), protocatechuic (PA-TPP^+^-C_10_), or salicylic acid (SA-TPP^+^-C_10_), along with doxycycline. Since hypoxic tumor cells frequently display increased resistance to anticancer agents, we evaluated the cytotoxic activity of all synthesized molecules in NCI-H727 and NCI-H1299 lung tumor cells under normoxic and hypoxic conditions. All compounds induced concentration-dependent reductions in cell viability, as shown by the concentration–response curves obtained for TPP^+^C_10_ (Figures 1B,C). IC_50_ values were calculated by nonlinear regression using a four-parameter model (Table 1).

**TABLE 1:** IC_50_ values for the tested molecules. The IC_50_ values were obtained from data analysis in GraphPad Prism using concentration-response curves with variable slope (four-parameter). Each value represents the average of at least 4 independent experiments in triplicate ± standard deviation.

			Drugs		IC_50_ ± SD (µM)		
Cell lines conditions			TPP^+^ C_10_	GA TPP^+^ C_10_	Doxycycline	PA-TPP^+^C_10_	SA-TPP^+^C_10_
NCI-H727	24 h	Normoxia	19.46 ± 0.54	10.48 ± 1.64	>100	13.27 ± 0.23	12.76 ± 0.63
		Hypoxia	24.22 ± 0.98	10.32 ± 0.65	>300	13.52 ± 1.24	13.00 ± 0.71
	48 h	Normoxia	12.37 ± 1.69	7.42 ± 0.71	21.63 ± 1.56	7.54 ± 0.54	6.46 ± 1.14
		Hypoxia	9.16 ± 1.21	6.98 ± 2.11	36.60 ± 2.11	12.34 ± 0.79	12.80 ± 1.20
	72 h	Normoxia	4.47 ± 0.90	1.75 ± 0.24	14.42 ± 0.18	5.73 ± 0.62	1.45 ± 0.14
		Hypoxia	4.04 ± 0.22	2.52 ± 0.49	14.22 ± 0.44	2.55 ± 0.13	0.94 ± 0.05
NCI-H1299	24 h	Normoxia	14.73 ± 2.73	10.66 ± 0.42	17.76 ± 0.87	9.93 ± 1.20	7.68 ± 0.343
		Hypoxia	19.36 ± 1.77	12.08 ± 2.03	37.20 ± 1.64	12.53 ± 1.17	7.93 ± 0.93
	48 h	Normoxia	9.94 ± 1.69	7.92 ± 1.10	16.39 ± 1.63	8.19 ± 1.12	1.25 ± 0.183
		Hypoxia	12.22 ± 1.33	9.96 ± 1.42	24.09 ± 1.02	8.27 ± 1.21	3.33 ± 0.34
	72 h	Normoxia	4.80 ± 0.82	2.65 ± 0.48	7.16 ± 0.35	3.29 ± 0.47	0.56 ± 0.145
		Hypoxia	5.02 ± 0.78	4.01 ± 0.92	15.47 ± 2.13	6.06 ± 1.03	0.54 ± 0.09
Lung Fibroblast	24 h	Normoxia	32.61 ± 1.08	12.34 ± 1.43	>300	8.86 ± 0.79	10.14 ± 1.65
	48 h	Normoxia	7.85 ± 0.70	6.73 ± 2.30	>300	5.13 ± 0.44	6.09 ± 1.04
	72 h	Normoxia	4.01 ± 1.00	2.37 ± 0.30	>300	10.96 ± 1.16	1.26 ± 0.08

For TPP^+^C_10_, cytotoxicity increased with longer incubation times in both tumor cell lines, as shown by a progressive decrease in IC_50_ values from 24 to 72 h under both normoxia and hypoxia. In NCI-H727 cells, IC_50_ values decreased from 19.46 ± 0.54 μM at 24 h to 4.47 ± 0.90 μM at 72 h under normoxia, and from 24.22 ± 0.98 µM to 4.04 ± 0.22 µM under hypoxia. A similar time-dependent pattern was observed in NCI-H1299 cells, where IC_50_ values decreased from 14.73 ± 2.73 μM at 24 h to 4.80 ± 0.82 μM at 72 h in normoxia, and from 19.36 ± 1.77 µM to 5.02 ± 0.78 µM under hypoxia (Figures 1D–G; Table 1). Among the tested derivatives, GA–TPP^+^C_10_ and SA–TPP^+^C_10_ consistently exhibited the highest cytotoxic potency. For example, in NCI-H727 cells at 72 h, GA–TPP^+^C10 reached IC_50_ values of 1.75 ± 0.24 µM (normoxia) and 2.52 ± 0.49 µM (hypoxia), while SA–TPP^+^C_10_ reached 1.45 ± 0.14 µM and 0.94 ± 0.05 µM, respectively. In NCI-H1299 cells, SA–TPP^+^C_10_ was also the most potent compound, with IC_50_ values of 0.56 ± 0.15 µM under normoxia and 0.54 ± 0.09 µM under hypoxia at 72 h. PA-TPP^+^C_10_ showed intermediate potency, whereas TPP^+^C10 was consistently less active than its GA and SA derivatives (Supplementary Figures S1–S14; Table 1).

Comparison between normoxic and hypoxic conditions revealed a modest reduction in drug potency at early time points under hypoxia, particularly at 24 h. However, these differences largely disappeared at 48 and 72 h, indicating that prolonged exposure overcomes the initial hypoxia-associated resistance in both tumor cell lines (Figures 1H,I; Table 1).

On the other hand, to assess selectivity toward cancer cells, we evaluated the effect of the compounds on non-malignant lung fibroblasts. At 24 h, IC_50_ values for TPP^+^C_10_, GA–TPP^+^C_10_, PA–TPP^+^C_10_ and SA–TPP^+^C_10_ tended to be higher in fibroblasts than in tumor cells, suggesting a possible trend toward lower early cytotoxicity in non-cancerous cells. However, these differences were modest and not consistently observed across all compounds. Nevertheless, after 72 h of exposure, IC_50_ values in fibroblasts became comparable to those observed in tumor cell lines for most compounds, suggesting limited long-term selectivity under the tested conditions (Figure 1J; Table 1, Supplementary Figure S15). Finally, doxycycline showed substantially lower cytotoxic activity than the mitochondria-targeted compounds. In NCI-H727 cells, no cytotoxicity was detected at 24 h, while at 48 and 72 h IC_50_ values ranged from 21.63 ± 1.56 µM to 14.42 ± 0.18 µM under normoxia, with slightly reduced potency under hypoxia (Figure 1K; Table 1). In NCI-H1299 cells, doxycycline exhibited consistently higher IC_50_ values under hypoxia at all-time points, confirming reduced efficacy in low oxygen conditions (Figure 1L; Table 1).

Based on the cytotoxicity screening, two mitochondria-targeted compounds were selected for subsequent mechanistic studies. TPP^+^C_10_ was included as the parental reference structure, while GA–TPP^+^C_10_ was chosen as the most promising derivative, displaying consistently lower IC_50_ values than TPP^+^C_10_ across both tumor cell lines and oxygen conditions. Although SA-TPP^+^C_10_ exhibited slightly higher cytotoxic potency, it also showed pronounced toxicity in non-cancerous lung fibroblasts at longer exposure times, suggesting a narrower therapeutic window. PA-TPP^+^C_10_ did not provide a clear advantage over GA-TPP^+^C_10_ in terms of potency or response consistency. Therefore, TPP^+^C_10_ and GA-TPP^+^C_10_ were selected for further evaluation of mitochondrial function.

### 3.2 Drug combination

**FIGURE 2:**
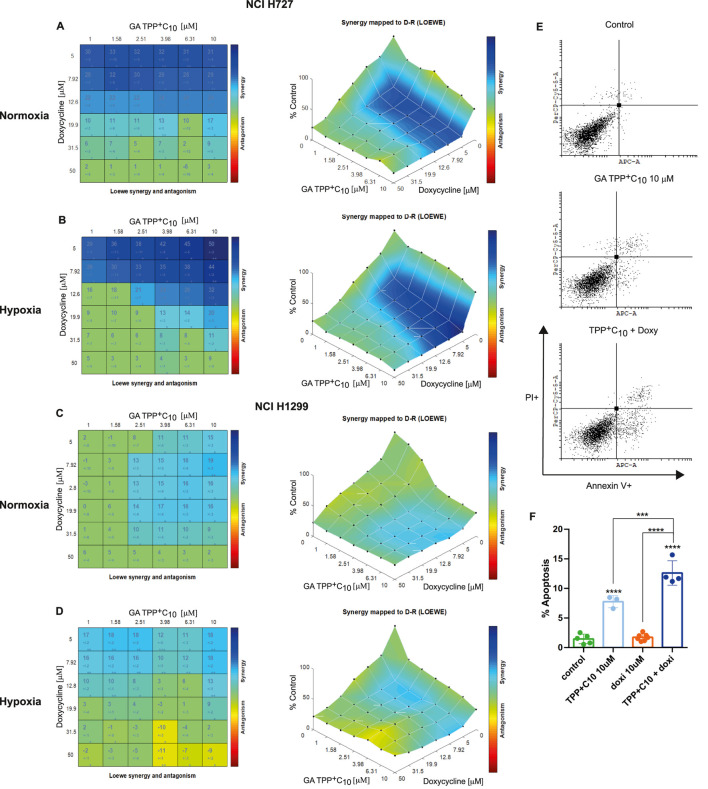
Synergic effect of TPP^+^C_10_ and doxycycline. **(A–D)** Synergistic effects of TPP^+^C_10_ and doxycycline on cell viability in **(A)** normoxic NCI-H727, **(B)** hypoxic NCI-H727, **(C)** normoxic NCI-H1299, and **(D)** hypoxic NCI-H1299 cells. Numbers in the combination matrix represent the combination index. **(E)** Flow cytometry analysis of NCI-H1299 cells treated with TPP^+^C_10_ (10 µM), doxycycline (10 µM), or their combination. **(F)** Quantification of apoptosis by flow cytometry. Data represent results from at least 3-4 independent experiments. Statistical significance: *p < 0.05; **p < 0.01; ***p < 0.001; ****p < 0.0001.

To determine the nature of the interaction between TPP^+^C_10_ or GA–TPP^+^C_10_ and doxycycline, combination assays were performed as described in the Methods section. Figure 2A shows the combination matrix and Loewe synergy map, illustrating the effect of TPP^+^C_10_ plus doxycycline on cell viability in NCI-H727 cells under normoxic conditions. Nineteen out of 36 drug combinations exhibited synergistic effects. Notably, most synergistic interactions occurred at low doxycycline concentrations, and no antagonistic effects were detected (Figure 2A). These findings were supported by flow cytometry-assessed apoptosis assays, which showed that the combination induced higher levels of apoptosis than either drug alone (Figures 2E,F). Under hypoxic conditions, the combination of TPP^+^C_10_ and doxycycline yielded fewer synergistic interactions, as expected. Nevertheless, the lowest doxycycline concentration still produced at least four synergistic combinations with TPP^+^C_10_, and no antagonistic effects were observed (Figure 2B). The NCI-H1299 cell line displayed greater resistance to the TPP^+^C_10_-doxycycline combination. Under normoxic conditions, only 12 of 36 combinations were synergistic, primarily at the highest concentrations of both drugs. In this cell line, two drug combinations exhibited antagonistic effects (Figure 2C). As anticipated, synergy further decreased under hypoxic conditions, with only 9 of 36 combinations showing significant synergy, and 2 showing antagonism (Figure 2D). Interestingly, in this setting, synergistic interactions persisted at low doxycycline concentrations.

**FIGURE 3:**
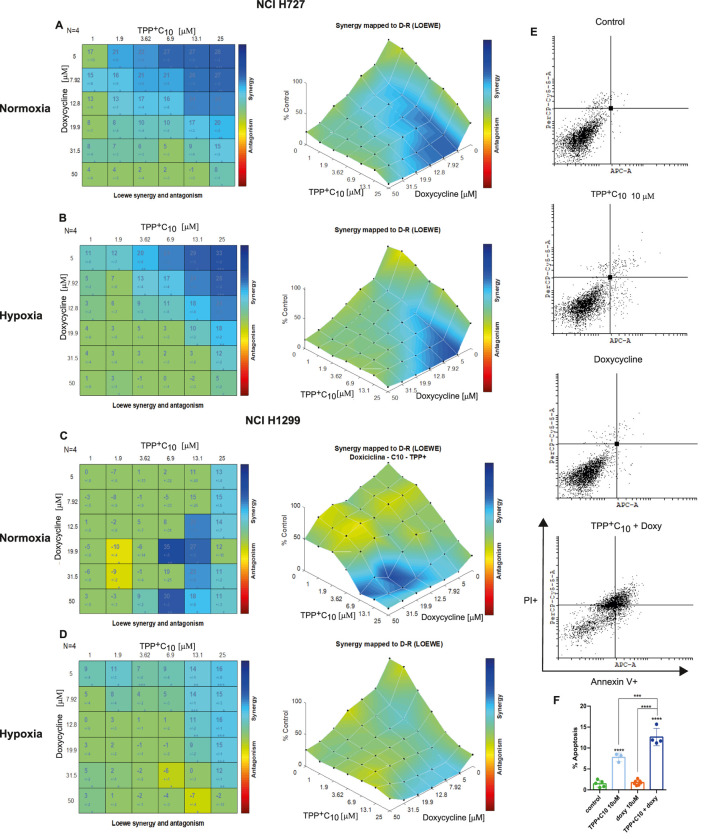
Synergic effect of GA-TPP^+^C_10_ and doxycycline. **(A–D)** Synergistic effects of GA-TPP + C10 and doxycycline on cell viability in **(A)** normoxic NCI-H727, **(B)** hypoxic NCI-H727, **(C)** normoxic NCI-H1299, and **(D)** hypoxic NCI-H1299 cells. Numbers in the combination matrix represent the combination index. **(E)** Flow cytometry analysis of NCI-H1299 cells treated with GA-TPP + C10 (10 µM), doxycycline (10 µM), or their combination. **(F)** Quantification of apoptosis by flow cytometry. Data represent results from 3 independent experiments.Statistical significance: *p < 0.05; **p < 0.01; ***p < 0.001; ****p < 0.0001.

We next evaluated the combination of GA-TPP^+^C_10_ with doxycycline. In NCI-H727 cells under normoxic conditions, at least 22 of 36 combinations showed synergistic effects, particularly at low concentrations of both compounds (Figure 3A). Apoptosis assays confirmed that the combination was more effective than monotherapies (Figures 3E,F). Under hypoxic conditions, the number of synergistic combinations slightly decreased; however, 20 combinations still showed significant synergy, while the remaining interactions were additive (Figure 3B). NCI-H1299 cells again exhibited higher resistance to the GA-TPP^+^C_10_-doxycycline combination. Although several synergistic interactions were observed, combination indices were consistently lower than those obtained in NCI-H727 cells (Figure 3C). Finally, under hypoxic conditions, NCI-H727 cells showed greater resistance to the GA-TPP^+^C_10_ and doxycycline combination. Nevertheless, 13 combinations remained synergistic, most occurring at low doxycycline concentrations. In addition, a small number of combinations showed antagonistic effects, particularly at high concentrations of both drugs tested (Figure 3D).

### 3.3 Effects on mitochondrial function

**FIGURE 4:**
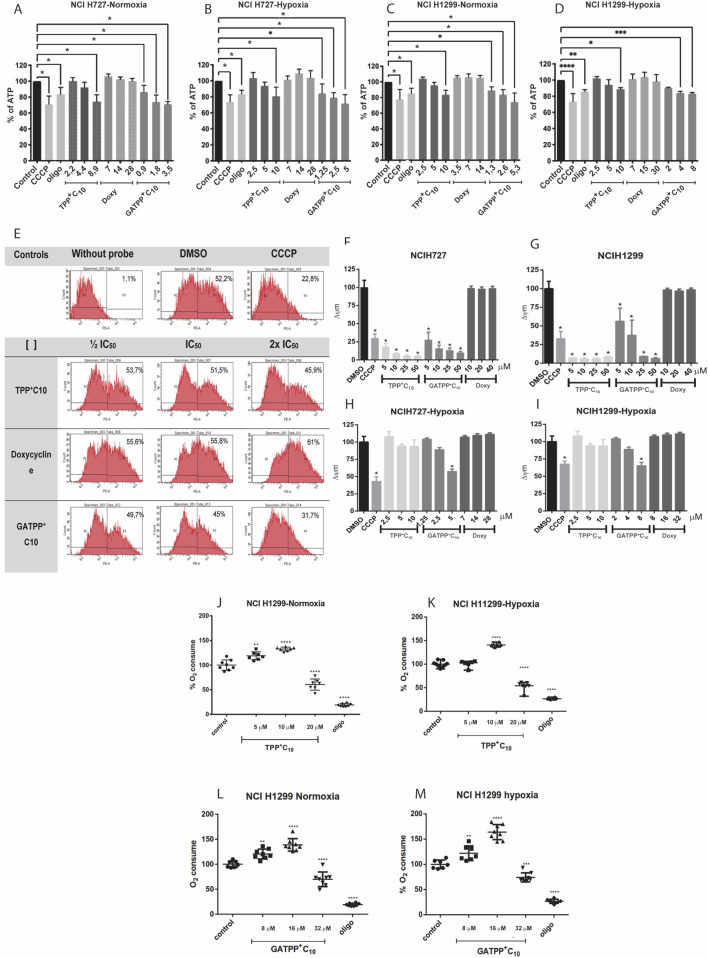
Mitochondrial functional inhibition induced by TPP + C10, GA-TPP + C10, and doxycycline. **(A–D)** Effects of TPP + C10, GA-TPP + C10, and doxycycline (Doxy) on cell viability in **(A)** normoxic NCI-H727, **(B)** hypoxic NCI-H727, **(C)** normoxic NCI-H1299, and **(D)** hypoxic NCI-H1299 cell lines. **(E)** Representative histograms showing the effects of the compounds on mitochondrial transmembrane potential. **(F–I)** Quantification of changes in mitochondrial membrane potential in **(F)** normoxic NCI-H727, **(G)** normoxic NCI-H1299, **(H)** hypoxic NCI-H727, and **(I)** hypoxic NCI-H1299 cells. **(J–M)** Oxygen consumption rate (OCR) in NCI-H1299 cells treated with TPP + C10, GA-TPP + C10, and doxycycline under **(J)** normoxic, **(K)** hypoxic, **(L)** normoxic, and **(M)** hypoxic conditions. Data represent results from at least 3-4 independent experiments Statistical significance: *p < 0.05; **p < 0.01; ***p < 0.001; ****p < 0.0001.

We next evaluated the impact of the selected compounds on mitochondrial function. First, intracellular ATP levels were measured in NCI-H727 and NCI-H1299 cells under normoxic and hypoxic conditions. Both TPP^+^C_10_ and GA-TPP^+^C_10_ induced a concentration-dependent reduction in ATP content levels in both tumor cell lines. In NCI-H727 cells under normoxia, GA-TPP^+^C_10_ produced a stronger decrease in ATP levels than TPP^+^C_10_ at equivalent concentrations, whereas under hypoxia, both compounds retained the ability to significantly reduce ATP despite an overall higher basal ATP content. A similar pattern was observed in NCI-H1299 cells, where GA-TPP^+^C_10_ consistently caused a more pronounced ATP depletion than TPP^+^C_10_. As expected, doxycycline did not significantly affect ATP levels under any of the tested conditions. CCCP and oligomycin were included as positive controls for mitochondrial uncoupling and ATP synthase inhibition, respectively, validating the assay performance (Figures 4A–D).

We then assessed mitochondrial transmembrane potential (Δψm) by flow cytometry. Under normoxic conditions, both TPP^+^C_10_ and GA-TPP^+^C_10_ induced a substantial loss of Δψm in NCI-H727 and NCI-H1299 cells, consistent with mitochondrial depolarization. In contrast, under hypoxic conditions, both cell lines exhibited partial resistance to Δψm dissipation, and a significant decrease in membrane potential was only detected at higher concentrations of GA-TPP^+^C_10_ (5 μM in NCI-H727 and 8 μM in NCI-H1299), whereas TPP^+^ C_10_ produced a weaker depolarizing effect. Doxycycline did not alter Δψm in any condition tested (Figures 4F–I).

Mitochondrial respiration was evaluated by measuring oxygen consumption rate (OCR). Both TPP^+^ C_10_ and GA-TPP^+^C_10_ exhibited a biphasic effect on OCR in the NCI-H1299 cell line. At low concentrations (½ IC_50_ and IC_50_), both compounds increased OCR, indicating a transient stimulation of mitochondrial respiratory activity. However, at higher concentrations (2×IC_50_), OCR was markedly reduced, consistent with mitochondrial respiration inhibition. This dual concentration-dependent behavior was consistently observed under both normoxic and hypoxic conditions. In contrast, doxycycline did not significantly modify OCR at the concentrations tested (Figures 4J–M). Finally, to determine whether the alterations in mitochondrial respiration were accompanied by changes in mitochondrial content, we next evaluated mitochondrial mass by flow cytometry. OCR and mitochondrial mass measurements were performed in NCI-H1299 cells under normoxic and hypoxic conditions, as this cell line displayed the most consistent and robust responses to mitochondria-targeted compounds in preliminary functional assays.

Treatment with TPP^+^ C_10_ alone significantly increased mitochondrial content compared with control cells, whereas doxycycline alone had no effect (Figure 4N). Notably, co-treatment with TPP^+^ C_10_ and doxycycline further enhanced mitochondrial mass compared with either treatment alone. A similar pattern was observed when NCI-H1299 cells were treated with GA-TPP^+^C_10_, where GA-TPP^+^C_10_ significantly increased mitochondrial content, doxycycline alone did not alter mitochondrial mass, and combined treatment with GA-TPP^+^C_10_ and doxycycline produced an increase in mitochondrial content (Figure 4O). The concordant increase in mitochondrial mass and stimulation of OCR at low compound concentrations suggests an early compensatory mitochondrial response, whereas the OCR inhibition observed at higher concentrations reflects subsequent mitochondrial functional impairment.

## 4 Discussion

### 4.1 Effect of TPP^+^ lipophilic cations conjugated to benzoate derivatives on cell viability

We investigated the cytotoxic effects of four delocalized lipophilic cations (TPP^+^ C_10_, GA- TPP^+^ C_10_, PA- TPP^+^ C_10_, and SA- TPP^+^ C_10_), and their combination with doxycycline, in two lung tumor cell lines (NCI-H727 and NCI-H1299) under normoxic and hypoxic conditions. Both cell lines are classified as non-small cell lung carcinoma and harbor p53 mutations (Lai et al., 2000; Eriksson et al., 2017), yet they exhibit distinct sensitivities to conventional antineoplastic drugs. Notably, NCI-H727 has been reported to be sensitive to MEK/ERK pathway inhibitors, whereas NCI-H1299 does not show this response (Iezzi et al., 2018). Compounds were tested at multiple concentrations and exposure times, and cell viability was assessed using the MTT assay. All compounds induced cytotoxicity in both NCI-H727 and NCI-H1299 cells, with effects increasing in a concentration- and time-dependent manner. Overall, as expected, cytotoxicity was higher under normoxic than hypoxic conditions. Moreover, differential sensitivity between cell lines was observed; for instance, TPP^+^C_10_ was more cytotoxic to NCI-H727 than to NCI-H1299.

In a previous study by our group in 2020, cell viability experiments were performed using these delocalized lipophilic cations in normoxic cultures of colon cancer cells. The IC_50_ values obtained in this study ranged from 5.3 to 15.2 μM at 24 h, from 2.7 to 14.2 μM at 48 h, and from 3.0 to 12.2 μM at 72 h, depending on the cell line and the compound used. Therefore, when comparing our IC_50_ values for NCI-H727 and NCI-H1299 tumor cell lines with those reported in the previous study, the IC50 values are similar. Furthermore, the study highlights the cytotoxic effects of cationic compounds, which are time- and concentration-dependent (Jara et al., 2020). Previous studies, also conducted by our group in breast cancer, have shown the cytotoxic effect of the compounds and their time- and concentration-dependent properties. Moreover, this cytotoxic effect is produced by an inhibition of mitochondrial function (Sandoval-Acuña et al., 2016).

Lung fibroblasts were used as a non-tumoral control cell line. Upon treatment with TPP^+^C_10_, cytotoxicity reached 92% at 24 and 48 h and 100% at 72 h. In contrast, all other lipophilic cationic benzoate derivatives showed 100% cytotoxicity at all tested concentrations, with IC50 values decreasing over time, as observed in tumor cell lines. Despite these *in vitro* findings, Peredo-Silva et al. investigated the effects of selected delocalized lipophilic cations in an *in vivo* model of mammary adenocarcinoma. In this work, the authors reported no evidence of systemic toxicity or organ damage in mice involved in compound metabolism and excretion, as determined by hematological and biochemical analyses. Histopathological examination of heart, liver, and kidney tissues further confirmed the absence of treatment-related toxicity (Peredo-Silva et al., 2017). These observations are consistent with previous reports describing the systemic safety of TPP^+^ cations *in vivo* models (Murphy, 2008).

Regarding the effect of doxycycline on cell viability in the NCI-H727 cell line, the results showed a concentration-dependent response, with higher concentrations producing greater cytotoxicity. This effect became evident only after 48 h of treatment and was not observed at 24 h. Additionally, IC_50_ values were higher in cultures exposed to hypoxia, consistent with previous findings showing that hypoxia can promote resistance to chemotherapy (Rohwer and Cramer, 2011; Zhang et al., 2014; Shang et al., 2025). The potential for hypoxia to induce a drug-resistant phenotype in our model is supported by previous reports in NCI-H1299 cells. It has been demonstrated that low oxygen tension significantly reduces the apoptotic response to DNA-damaging agents such as cisplatin (Deben et al., 2018). This chemoresistance is often mediated by the HIF-1α/PI3K/Akt signaling axis, which promotes cell survival and metabolic adaptation (Fu et al., 2026). Furthermore, hypoxia-induced stabilization of HIF-1α upregulates the expression of multidrug resistance genes (e.g., MDR1), thereby facilitating drug efflux. Therefore, although not directly measured in the present study, the hypoxic conditions established in our experiments are consistent with the molecular environment required for the development of therapeutic resistance in NSCLC.

Similar results were observed on the NCI-H1299 cell line. In this case, the impact of hypoxia on drug cytotoxicity depended on both concentration and exposure time, with consistently higher IC_50_ values in hypoxic cultures across all time points evaluated. Previous studies have evaluated doxycycline in breast cancer cell lines for 24 h, reporting cytotoxic effects at concentrations higher than those observed in our NCI-H1299 cell line (70.5–109.3 µM vs. 17.7 µM) (Fuentes-Retamal et al., 2020). Additionally, other studies in breast cancer have reported IC_50_ values of 11.39 µM and 7.13 µM in MCF-7 and MDA-MB-468 cells, respectively, after 72 h of incubation. These studies also demonstrated effects on spheroid formation, with IC_50_ values of 37 µM and 27 μM, respectively, at 72 h (Zhang et al., 2017).

In contrast to the tested compounds, which exhibited a pronounced cytotoxic effect on fibroblasts, doxycycline yielded indeterminate IC_50_ values in this cell line because the concentrations tested were insufficient to generate an accurate dose-response curve. This suggests that doxycycline may exert cytotoxic effects on fibroblasts only at substantially higher concentrations (Holmes and Charles, 2009). This observation is not unexpected, as doxycycline has been in clinical use since 1967, has a well-established safety profile, and its pharmacological dosing ranges are extensively documented in the literature (Holmes and Charles, 2009; Navarro-Triviño et al., 2020; Hassan et al., 2024).

### 4.2 Synergistic effects on cell viability

A significant number of combinations of benzoate-derived lipophilic cations and doxycycline exhibited synergistic effects on cell viability. This synergy was consistently observed in both NCI-H727 and NCI-H1299 cell lines. Of particular interest, combinations displaying strong synergy (high combination index) occurred at low concentrations of both TPP^+^C_10_ and GA-TPP^+^C_10_. Furthermore, a considerable number of synergistic interactions were also detected under hypoxic conditions.

Fuentes-Retamal and colleagues investigated the effects of GA-TPP^+^C_10_ in breast cancer cell lines and reported that this compound induces metabolic stress, leading to increased expression of peroxisome proliferator-activated receptor gamma coactivator 1α (PGC-1α) (Fuentes-Retamal et al., 2020). PGC-1α is a key regulator of mitochondrial biogenesis, and its expression is controlled by AMP-activated protein kinase (AMPK), a central sensor of cellular energy status. Under conditions of reduced ATP availability, AMPK becomes activated and promotes PGC-1α expression, which, in turn, drives the transcription of genes involved in mitochondrial biogenesis, ultimately increasing mitochondrial mass and function (Herzig and Shaw, 2018). These results agree with ours because our lipophilic cations can, in a compensatory manner, induce mitochondrial mass expansion via a mitochondrial biogenesis mechanism. Besides, doxycycline binds to the 28S subunit of mitochondrial ribosomes (Fuentes-Retamal et al., 2020), which prevents the formation of polypeptide chains. Polypeptide chains are essential for protein synthesis; therefore, inhibition of their formation reduces the production of proteins necessary for mitochondrial biogenesis (Moullan et al., 2015). This can lead to metabolic collapse in cells and trigger a compensatory increase in mitochondrial biogenesis. The use of TPP^+^C_10_ or GA-TPP^+^C_10_ in combination with doxycycline would therefore inhibit tumor cells’ response to mitochondrial dysfunction, increasing observed cell death, while at the same time contributing to a compensatory response that indirectly induces mitochondrial biogenesis.

### 4.3 Inhibitory effects on mitochondrial function

Regarding intracellular ATP levels, the results showed that in NCI-H727 cells, TPP^+^C_10_ induced a significant decrease only at the highest concentration tested, corresponding to twice the IC_50_ value. In contrast, GA-TPP^+^C_10_ reduced ATP levels at all concentrations evaluated, which were lower than those required for TPP^+^C_10_. Very similar results were obtained in the NCI-H1299 cell line. Overall, ATP depletion was concentration-dependent, and the magnitude of the decrease closely resembles that reported by Sandoval-Acuña et al. in breast cancer cell lines (Sandoval-Acuña et al., 2016). In contrast, doxycycline did not significantly alter ATP levels compared with control conditions, regardless of concentration or cell line. This is consistent with its distinct mechanism of action. Delocalized lipophilic cations target the mitochondrial electron transport chain, inducing mild uncoupling and/or inhibition of oxidative phosphorylation. Doxycycline, by comparison, binds to the 28S subunit of mitochondrial ribosomes, impairing mitochondrial protein synthesis and biogenesis without directly affecting ATP production (Lin et al., 2018; Zhang et al., 2017). Moreover, both cations induced a concentration-dependent decrease in mitochondrial transmembrane potential in the 2 cell lines examined. This effect is consistent with previous observations in oral, breast, and colorectal cancer cells, as well as with other gallic acid–derived lipophilic cations (Jara et al., 2014; Catalán et al., 2020; Jara et al., 2020). Notably, although the reduction in membrane potential was less pronounced under hypoxic conditions, it remained clearly detectable. This is a relevant finding, as it suggests that these cations may retain activity in hypoxic tumor microenvironments.

Finally, our results suggest that TPP^+^C_10_ and GA-TPP^+^C_10_ exerted a dual effect on cellular respiration. At low concentrations, they appear to act as mild uncouplers, increasing oxygen consumption. At higher concentrations, they inhibit respiration, reducing oxygen consumption. This biphasic behavior may result from an initial dissipation of the negative charge on the inner mitochondrial membrane, followed by inhibition of one or more complexes of the electron transport chain. Similar concentration-dependent effects of delocalized lipophilic cations on mitochondrial respiration have been reported in other cancer models (Trendeleva et al., 2013; Trnka et al., 2015; Yang et al., 2017). Further experiments are required to confirm the precise molecular targets involved. While respiratory inhibition and subsequent electron leakage from the transport chain are well-established triggers of reactive oxygen species (ROS) generation, our previous findings in other cancer models suggest that the cytotoxicity of these lipophilic cations is primarily driven by mitochondrial uncoupling rather than by direct ROS production (Jara et al., 2020). The biphasic effect observed in the OCR measurements, characterized by an initial stimulation followed by collapse, further supports a mechanism of action centered on targeted mitochondrial destabilization and bioenergetic failure.

We also observed a significant increase in mitochondrial mass in cells treated with TPP^+^C_10_ or GA- TPP^+^C_10_. The concordant rise in mitochondrial content and stimulation of oxygen consumption at low compound concentrations suggests the activation of a compensatory mitochondrial response aimed at counteracting early mitochondrial stress. Such adaptive increases in mitochondrial biogenesis have been described as a survival mechanism in cancer cells exposed to mitochondrial dysfunction (Vellinga et al., 2015; Crosby et al., 2020)

Doxycycline alone did not alter mitochondrial mass, consistent with its role as an inhibitor of mitochondrial protein synthesis rather than a direct inducer of biogenesis. However, combined treatment with the cations further increased mitochondrial mass, despite the concurrent impairment of mitochondrial function. In this sense, we found it challenging to observe a synergistic effect at the mitochondrial level, as the timing of our measurements did not align with the delayed effects of doxycycline on mitochondrial biogenesis. This contrasts with mitochondrial parameters such as ATP content, oxygen consumption rate (OCR), and mitochondrial transmembrane potential, which reflect early functional changes and are measured after short incubation times with the compounds. Consequently, demonstrating a synergistic effect on mitochondrial function beyond the cytotoxicity observed with the combined treatment remains a technical challenge.

### 4.4 Study limitations

Among the limitations of this study is the need for a more detailed characterization of the mechanisms underlying mitochondrial function inhibition, including direct quantification of ROS levels and evaluation of specific mitochondrial protein markers. Such data would clarify whether oxidative stress acts as a primary or secondary driver of the observed cell death in these NSCLC cell lines. Additionally, further combination experiments are required to directly demonstrate synergistic effects on mitochondrial function. It would also be of interest to enrich cell cultures for cancer stem-like cells by prolonged incubation under hypoxic conditions and to evaluate compound activity in this model. Finally, *in vivo* studies are necessary to validate the effects observed on cell viability and to assess the antineoplastic potential of these compounds and their combination.

## 5 Conclusion

Lipophilic cations derived from gallic acid metabolites demonstrated cytotoxic activity against non-small cell lung carcinoma cells under both normoxic and hypoxic conditions. These compounds also retained cytotoxic effects in models associated with drug-resistant phenotypes. In addition, the combination of TPP^+^C_10_ or GA-TPP^+^C_10_ with doxycycline produced a synergistic reduction in cell viability under both oxygen conditions, consistent with the proposed mitochondrial-targeting mechanisms of these molecules.

Our results suggest that the observed cytotoxicity may be associated with mitochondrial dysfunction leading to tumor cell death. However, these findings are based on *in vitro* models, and further studies are required to validate their therapeutic relevance. Future investigations should incorporate more physiologically relevant models, including multicellular spheroids that reproduce tumor-like oxygen gradients, as well as *in vivo* studies to evaluate the efficacy of these combinations in reducing tumor volume, improving survival outcomes, and assessing safety profiles to confirm the clinical relevance of this dual-targeting strategy.

## Data Availability

The original contributions presented in the study are included in the article/Supplementary Material, further inquiries can be directed to the corresponding authors.

## In memoriam

In memory of Dr. Jorge Ferreira Parker (R.I.P.).
