# Supplementary material for: Overcoming resistance in lung cancer: combined mitochondrial destabilization by lipophilic cations and doxycycline in hypoxic non-small cell lung carcinoma
Source: Front Pharmacol. 2026 May 4;17:1797291. doi: 10.3389/fphar.2026.1797291 (PMC13180888; doi:10.3389/fphar.2026.1797291)
Supplement: Supplementary file 1 [file DataSheet1.docx]

Supporting Information

**Synergistic cytotoxic effect of lipophilic cations derived from gallic acid metabolites and doxycycline in non-small cell lung cancer *in vitro*.**

*Denny Vidal^1^.; Mabel Catalán^2^.; Ivonne Olmedo^3^.; Alfredo Molina-Berríos^1^.; Javiera Carrasco-Castillo.^4^; Rodrigo López-Muñoz^5^.; Jorge Ferreira^1†^ and José A. Jara ^1*^.*

^1^Institute for Research in Dental Sciences (ICOD), Faculty of Dentistry, Universidad de Chile, Olivos 943, 8380544, Santiago, Chile.

^2^Clinical and Molecular Pharmacology Program, Institute of Biomedical Sciences (ICBM), Faculty of Medicine, University of Chile, Independencia 1027, 8380453, Santiago, Chile.

^3^Physiopathology Program, Institute of Biomedical Sciences (ICBM), Faculty of Medicine, University of Chile, Independencia 1027, 8380453, Santiago, Chile.

^4^Center for Regenerative Medicine, School of Medicine, Clínica Alemana-Universidad del Desarrollo, 7610658, Santiago, Chile.

^5^Instituto de Farmacología y Morfofisiología, Facultad de Ciencias Veterinarias, Universidad Austral de Chile, 5090010, Valdivia, Chile.

*Corresponding Author: ^1^Institute for Research in Dental Sciences (ICOD), Faculty of Dentistry, Universidad de Chile, Santiago, Chile.  Phone: +56 2 29781730- +56 2 29781718. E-mail: [jsandovalj@u.uchile.cl](mailto:jsandovalj@u.uchile.cl).

^†^ R.I.P.

**Supplementary figures**

**a. TPP^+^ C10**

**Cell line NCI–H727**

|  | **24 h** | **48 h** | **72 h** |
| --- | --- | --- | --- |
| **Normoxic** | **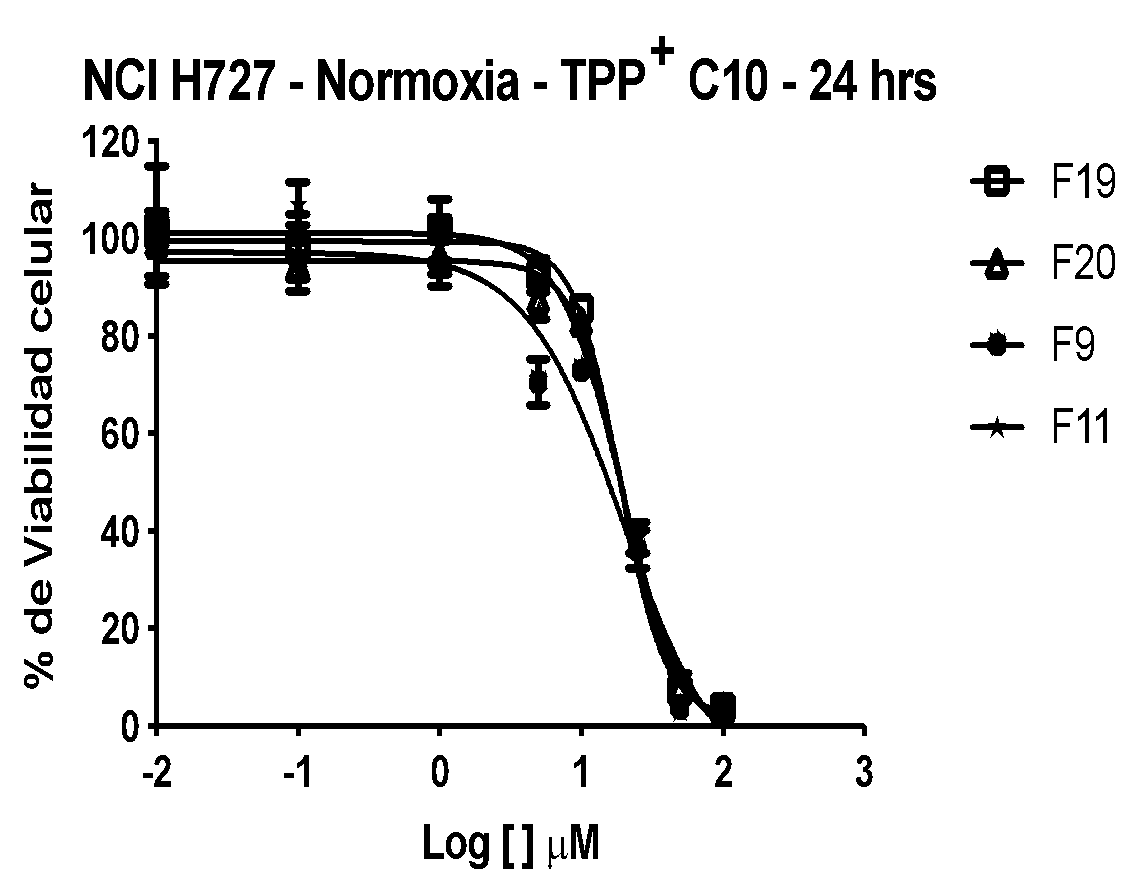**  **A** | **B**  **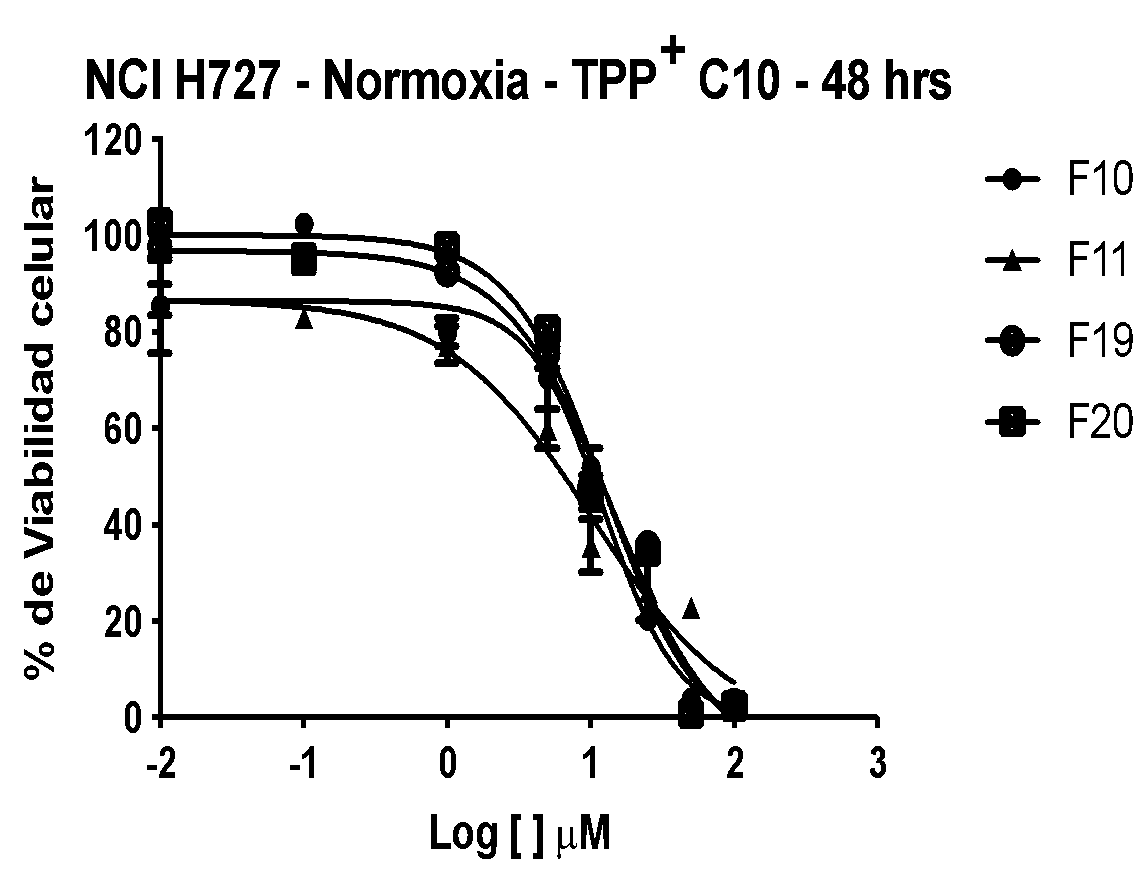** | **C**  **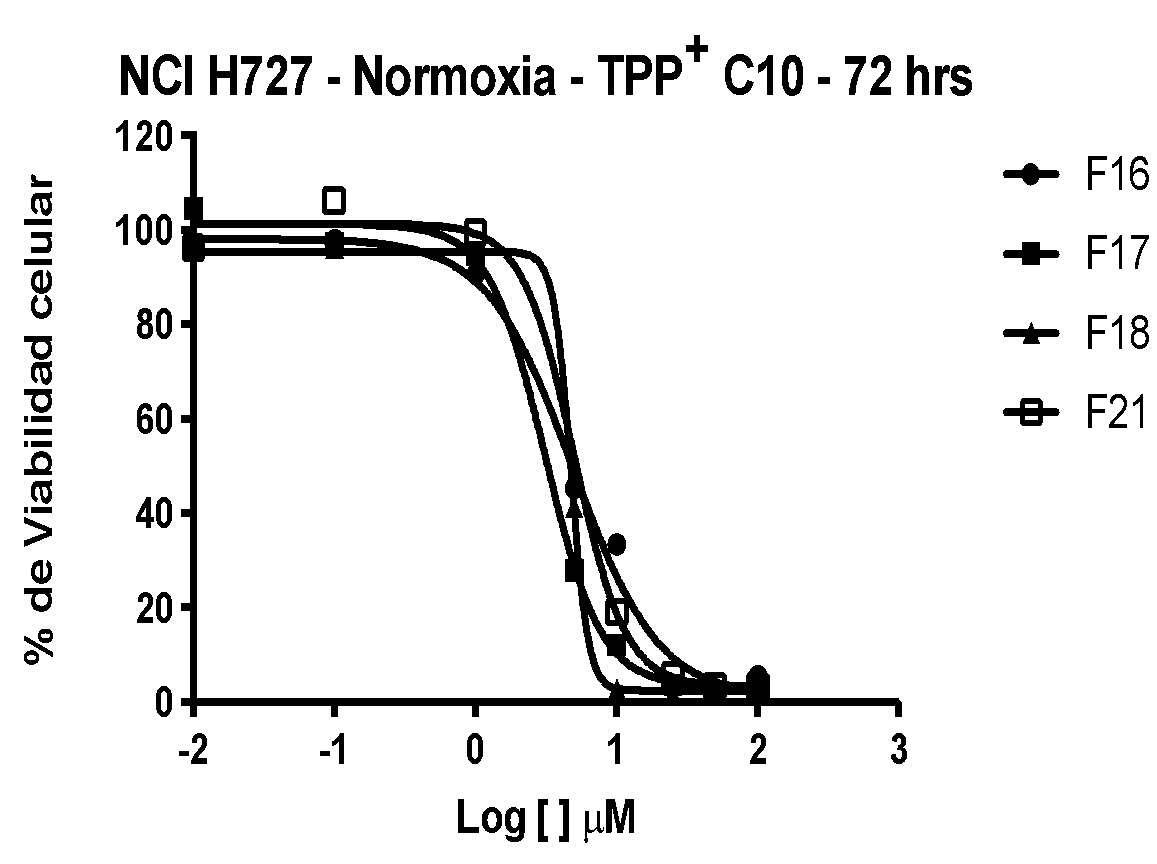** |
| **IC_50_** | 19,46 ± 0,54 *μM* | 12,37 ± 1,69 *μM* | 4,47 ± 0,9 *μM* |
| **Hypoxia** | **D**  **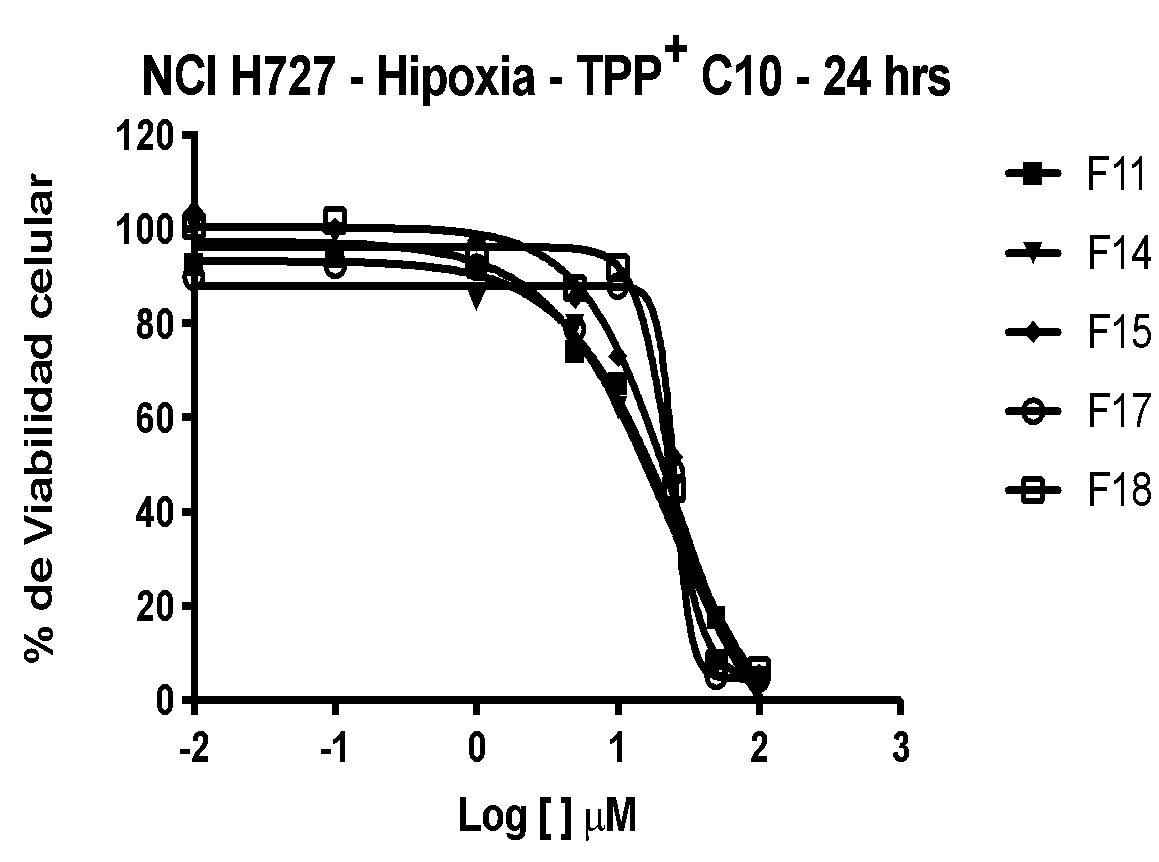** | **E**  **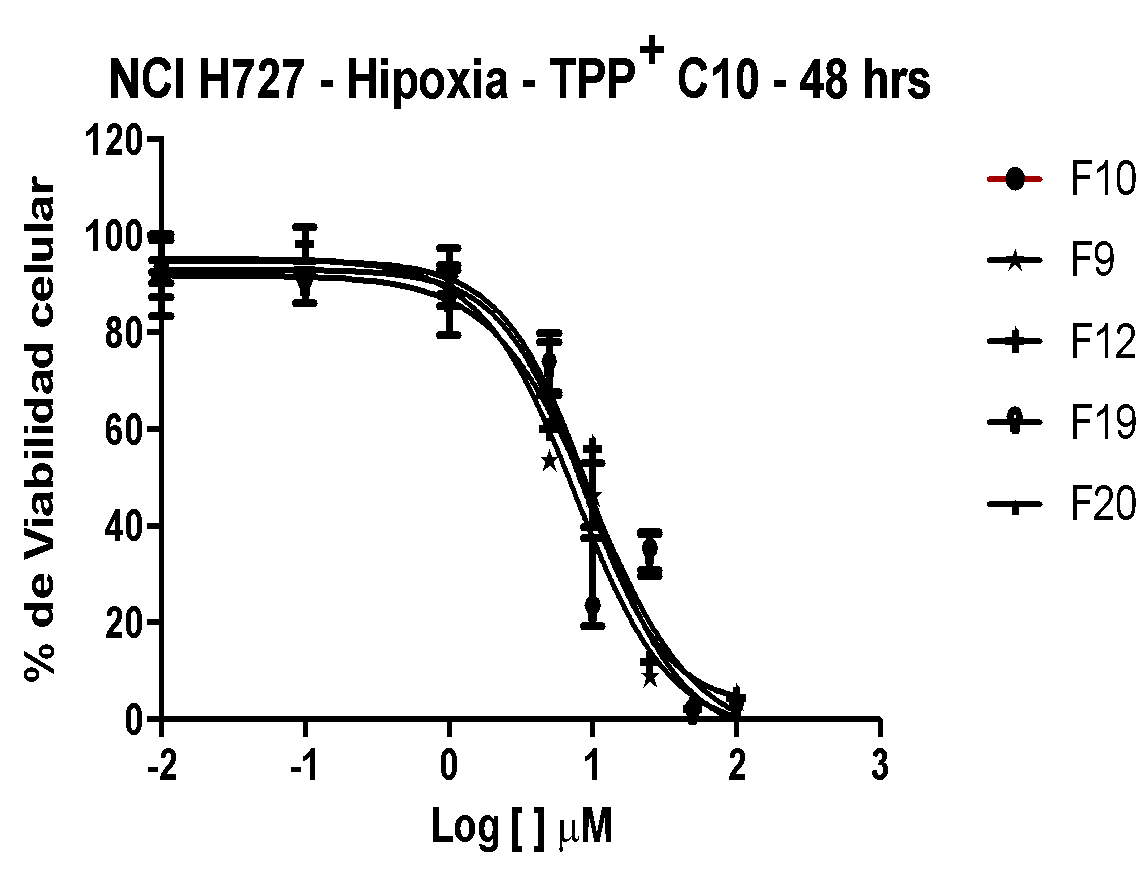** | **F**  **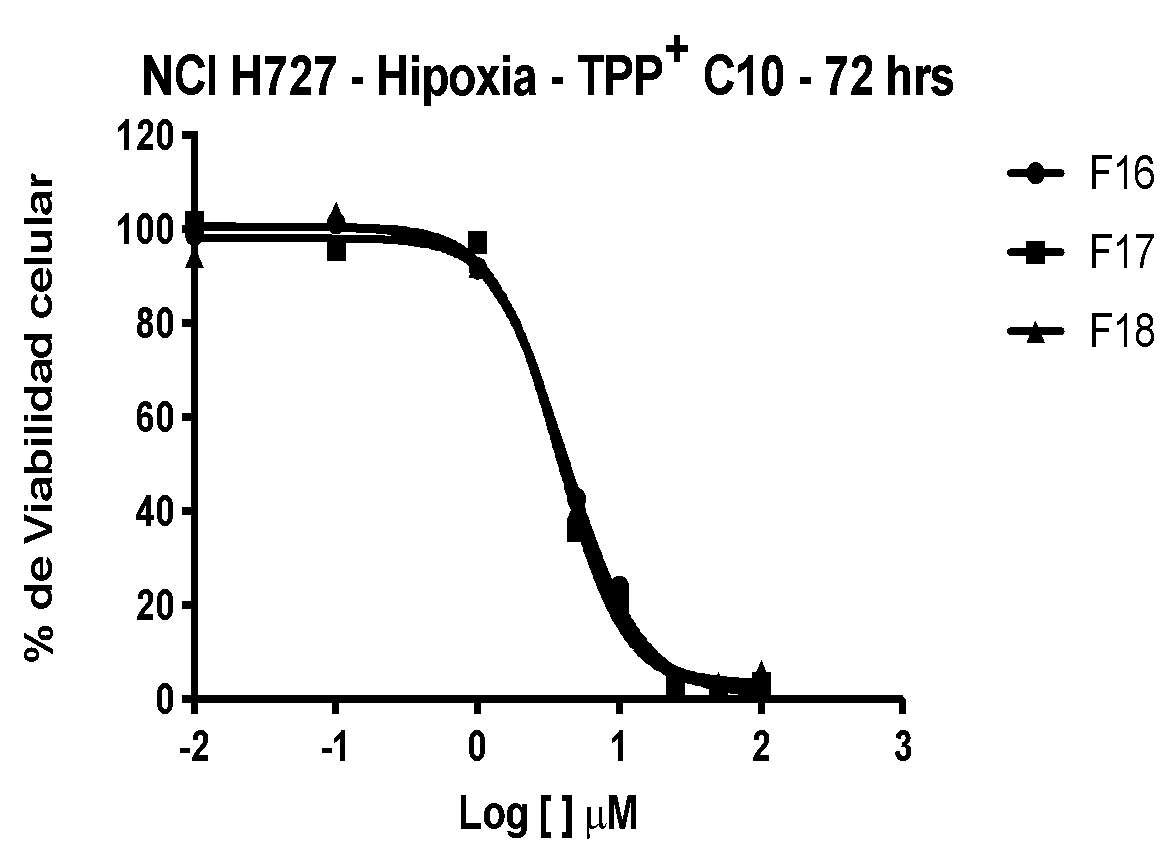** |
| **IC_50_** | 24,22 ± 0,98 *μM* | 9,16 ± 1,21 *μM* | 4,04 ± 0,22 *μM* |

**Figure. S1.** Cytotoxic effect of Galico-TPP+C10 on NCI H727 cell line monolayer cultures under normoxic and hypoxic conditions. Graphical representation of the effect of the compound TPP+C10 on cell viability as a function of the logarithm of the concentration. The cytotoxic effect is observed at 24 hr (A and D), 48 hr (B and E) and 72 hr (C and F). Data obtained by averaging 5 independent experiments. The averages of the IC_50_ ± SD are shown.

**Cell line NCI–H1299**

|  | **24 h** | **48 h** | **72 h** |
| --- | --- | --- | --- |
| **Normoxic** | **A**  **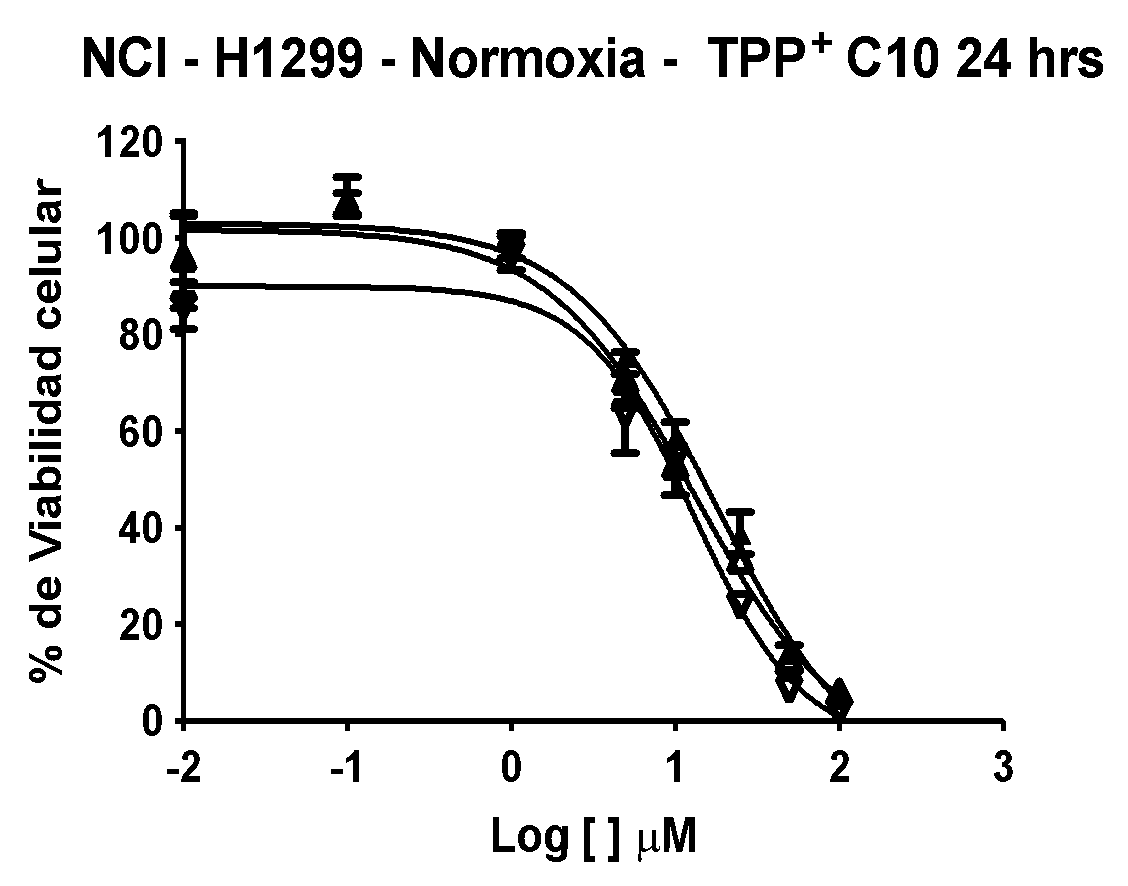** | **B**  **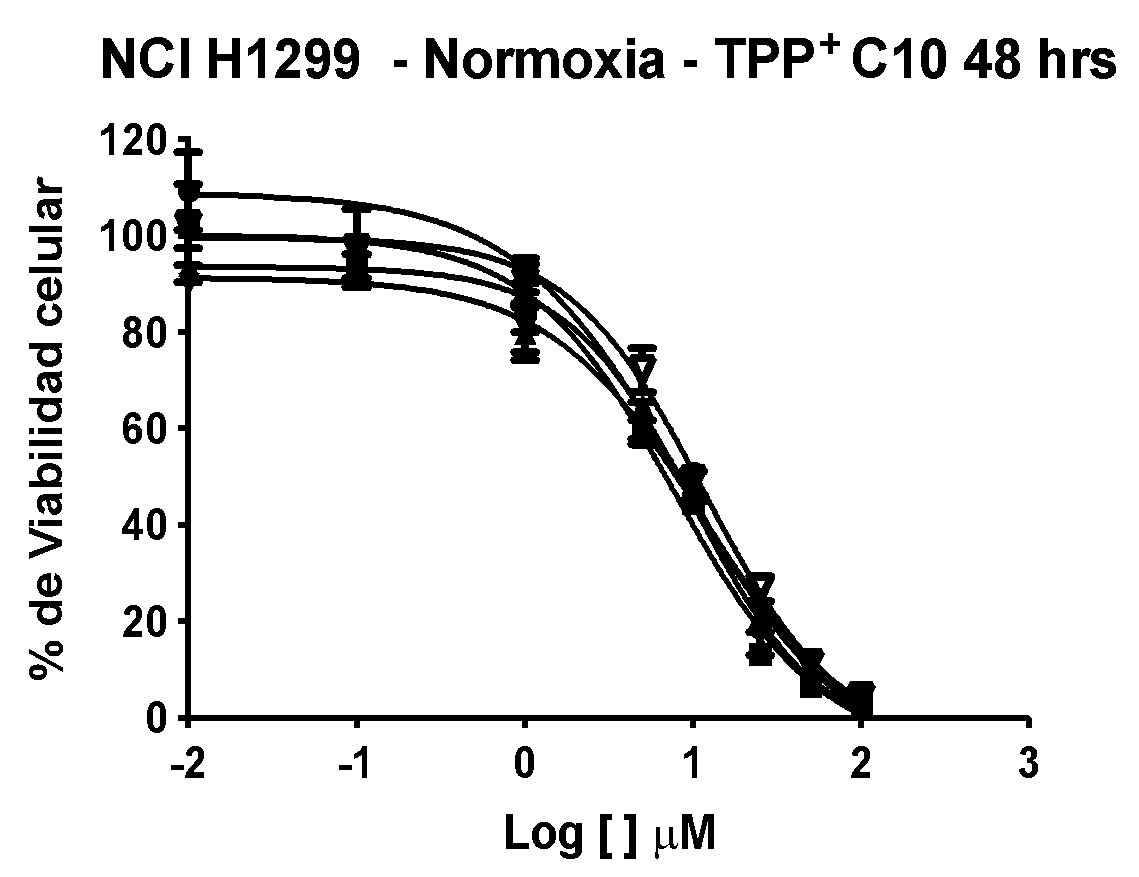** | **C**  **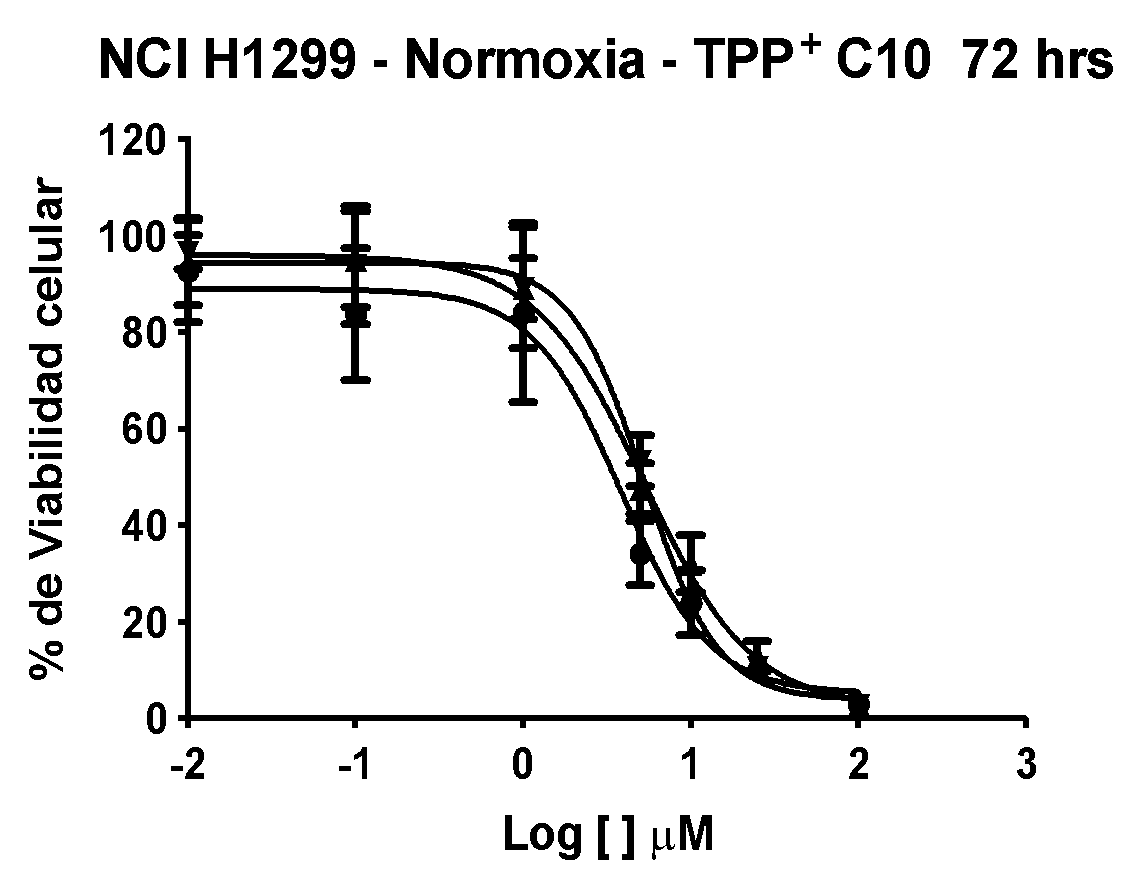** |
| **IC_50_** | 14,73 ± 2,73 *μM* | 9,94 ± 1,69 *μM* | 4,8 ± 0,82 *μM* |
| **Hypoxic** | **D**  **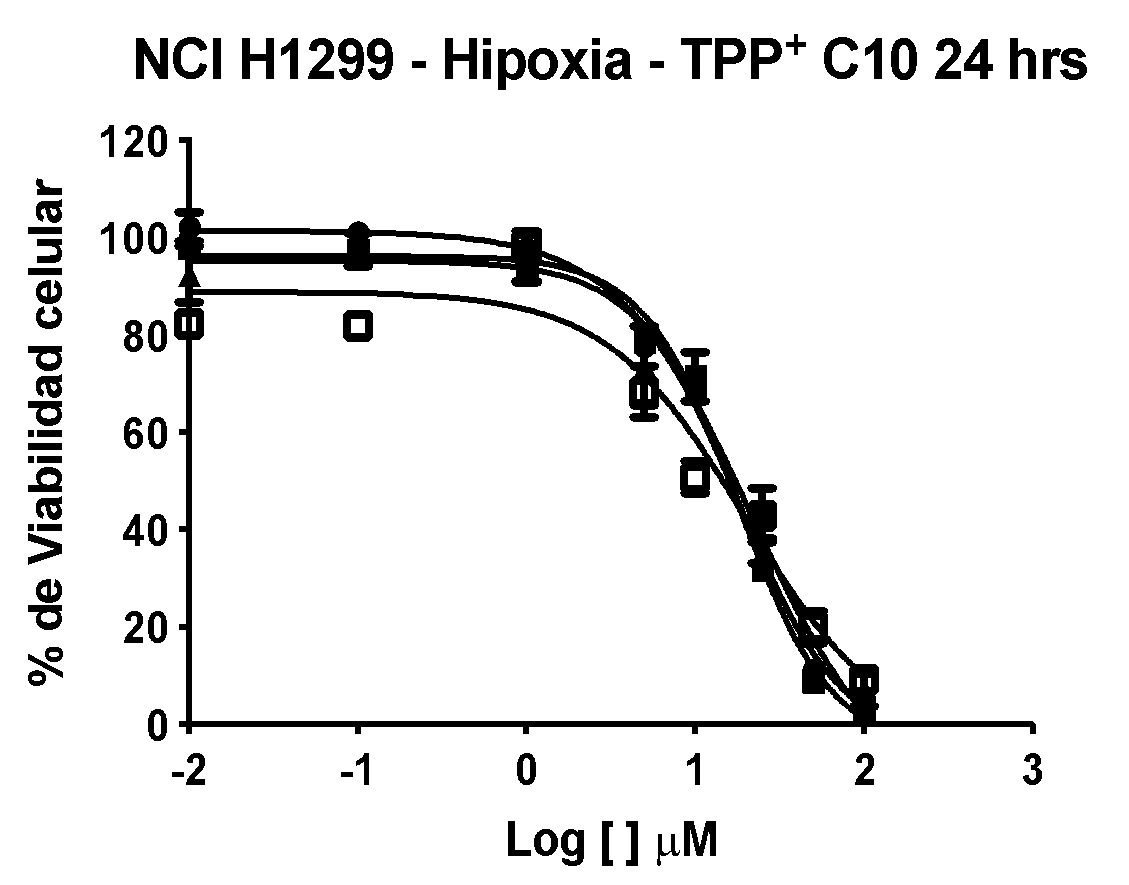** | **E**  **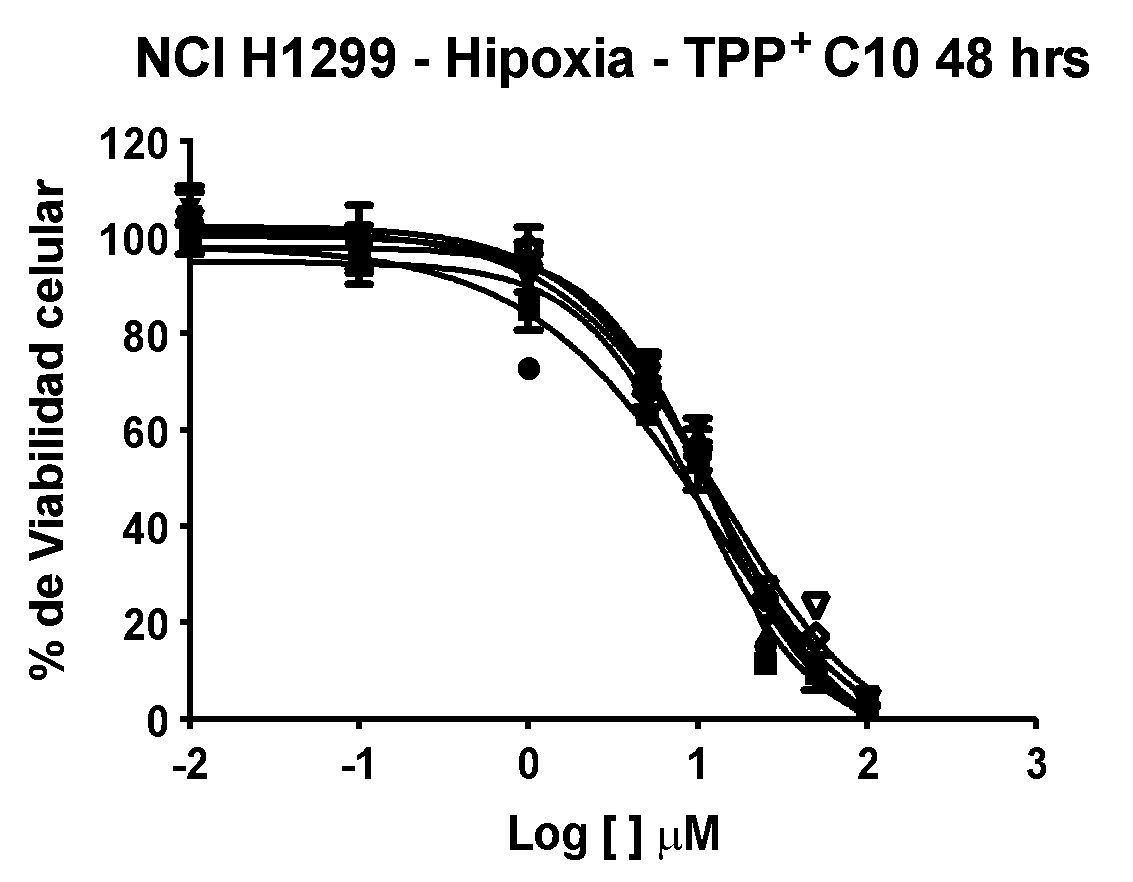** | **F**  **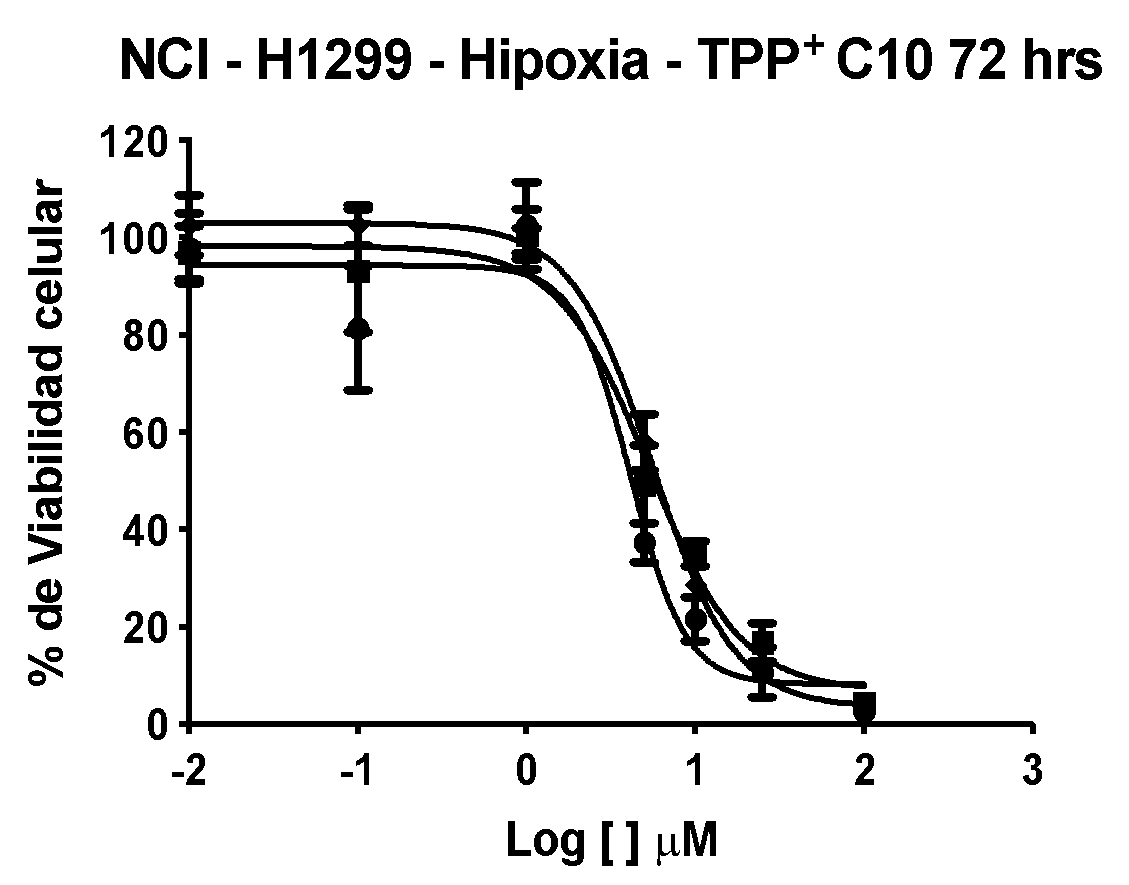** |
| **IC_50_** | 19,36 ± 1,77 *μM* | 12,22 ± 1,33 *μM* | 5,02 ± 0,78 *μM* |

**Figure. S2.** Cytotoxic effect of TPP^+^C_10_ on NCI H1299 cell line monolayer cultures under normoxic and hypoxic conditions. Graphical representation of the effect of the compound TPP^+^C_10_ on cell viability as a function of the logarithm of the concentration. The cytotoxic effect is observed at 24 h (A and D), 48 h (B and E) and 72 h (C and F). Data obtained by averaging 5 independent experiments. The averages of the IC_50_ ± SD are shown.

**Lung Fibroblasts**

|  | **24 h** | **48 h** | **72 h** |
| --- | --- | --- | --- |
| **Normoxic** | **A**  **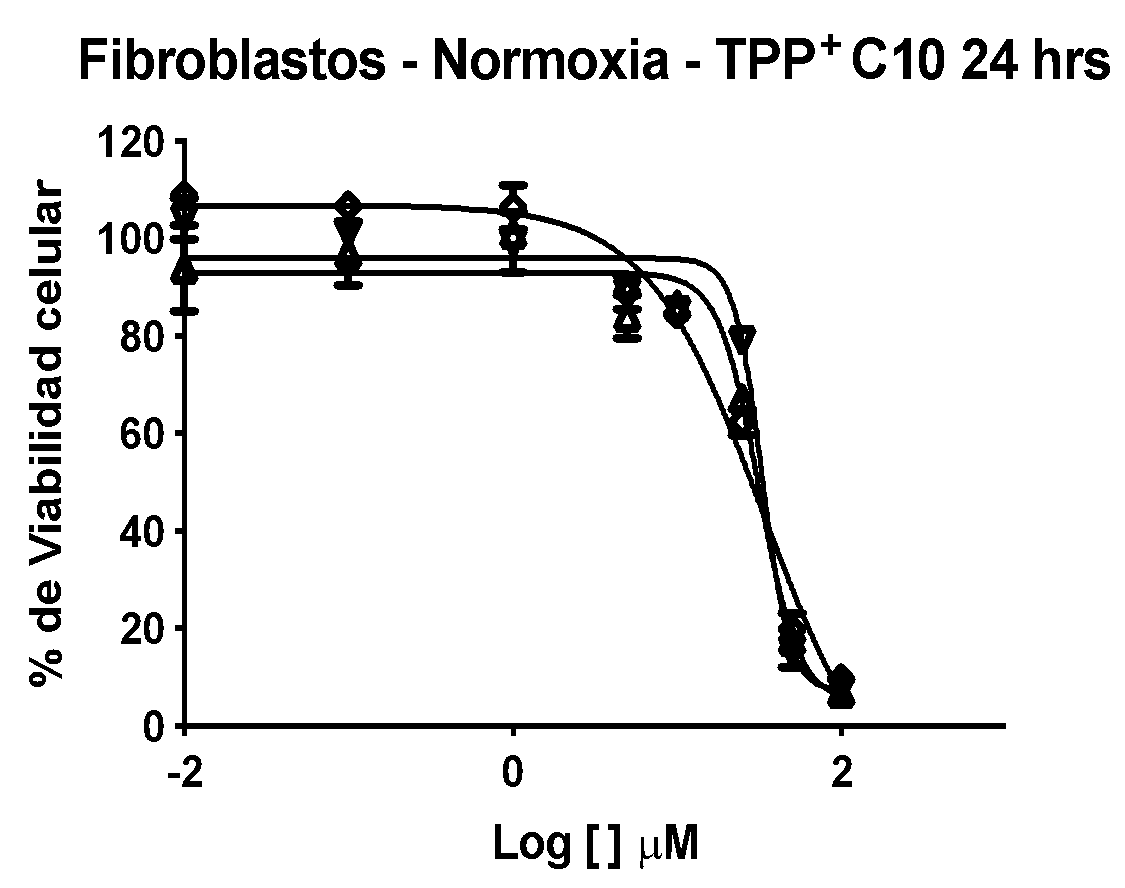** | **B**  **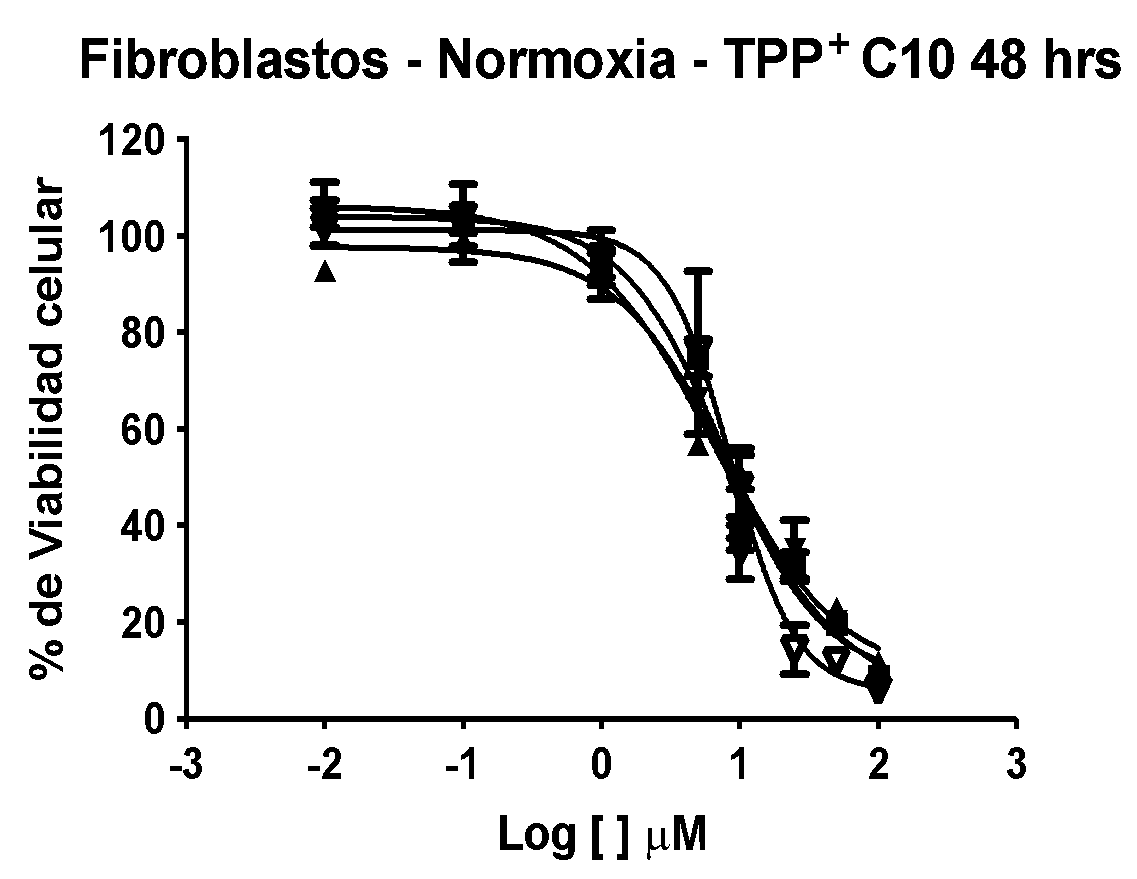** | **C**  **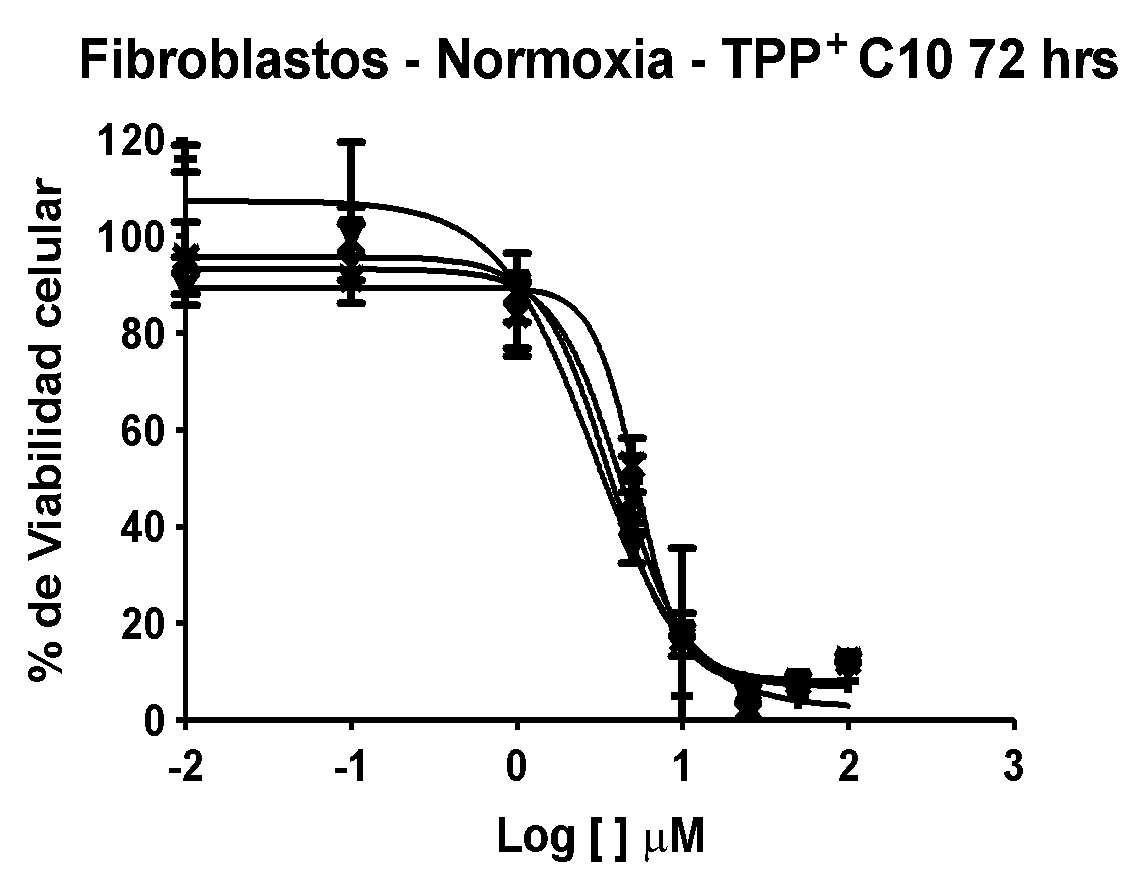** |
| **IC_50_** | 32,61 ± 1,08 *μM* | 7,85 ± 0,7 *μM* | 4,01 ± 1,0 *μM* |

**Figure. S3.** Cytotoxic effect of TPP^+^C_10_ on monolayer cultures of lung fibroblast cell lines under normoxic conditions. Graphical representation of the effect of the compound TPP^+^C_10_ on cell viability as a function of the logarithm of the concentration. The cytotoxic effect is observed at 24 h (A), 48 h (B) and 72 h (C). Data obtained by averaging 5 independent experiments. The averages of the IC_50_ ± SD are shown.

**b. GA TPP^+^ C10**

**Cell line NCI – H727**

|  | **24 h** | **48 h**  **B** | **72 h** |
| --- | --- | --- | --- |
| **Normoxic** | **A**  **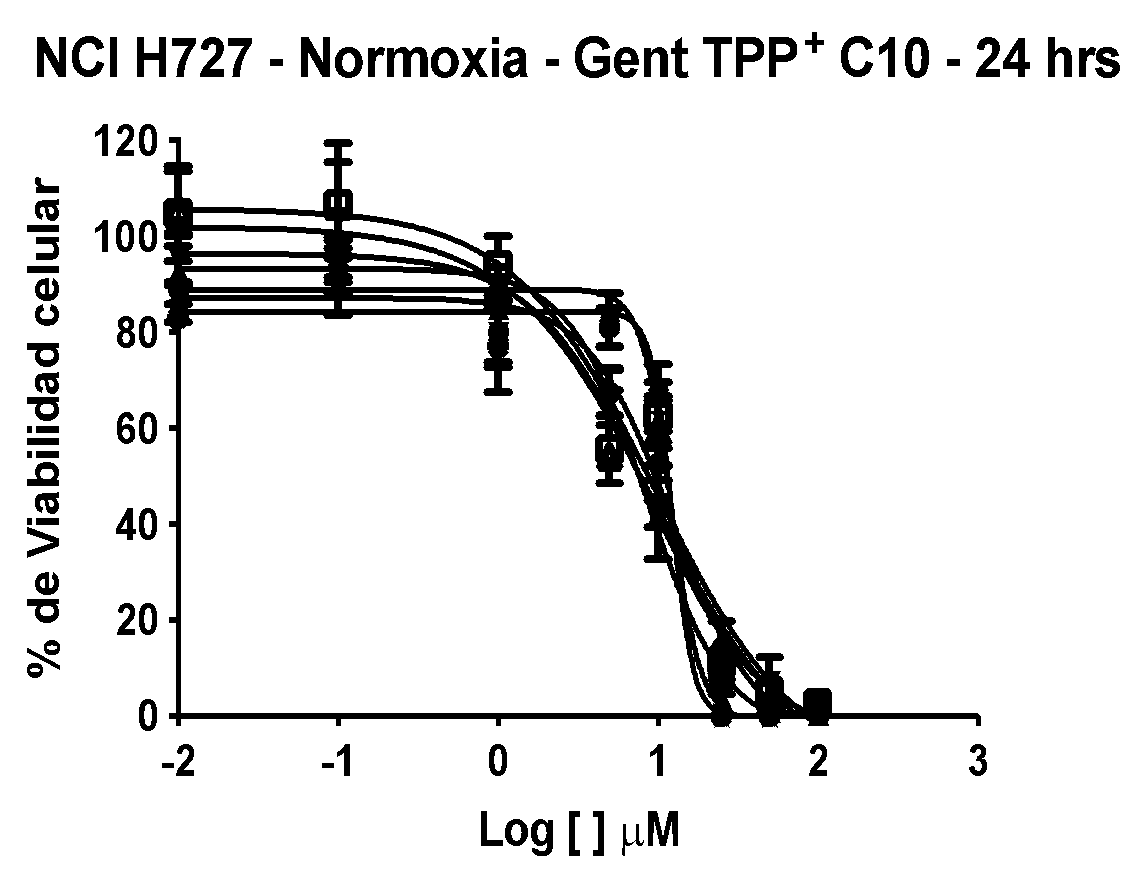** | **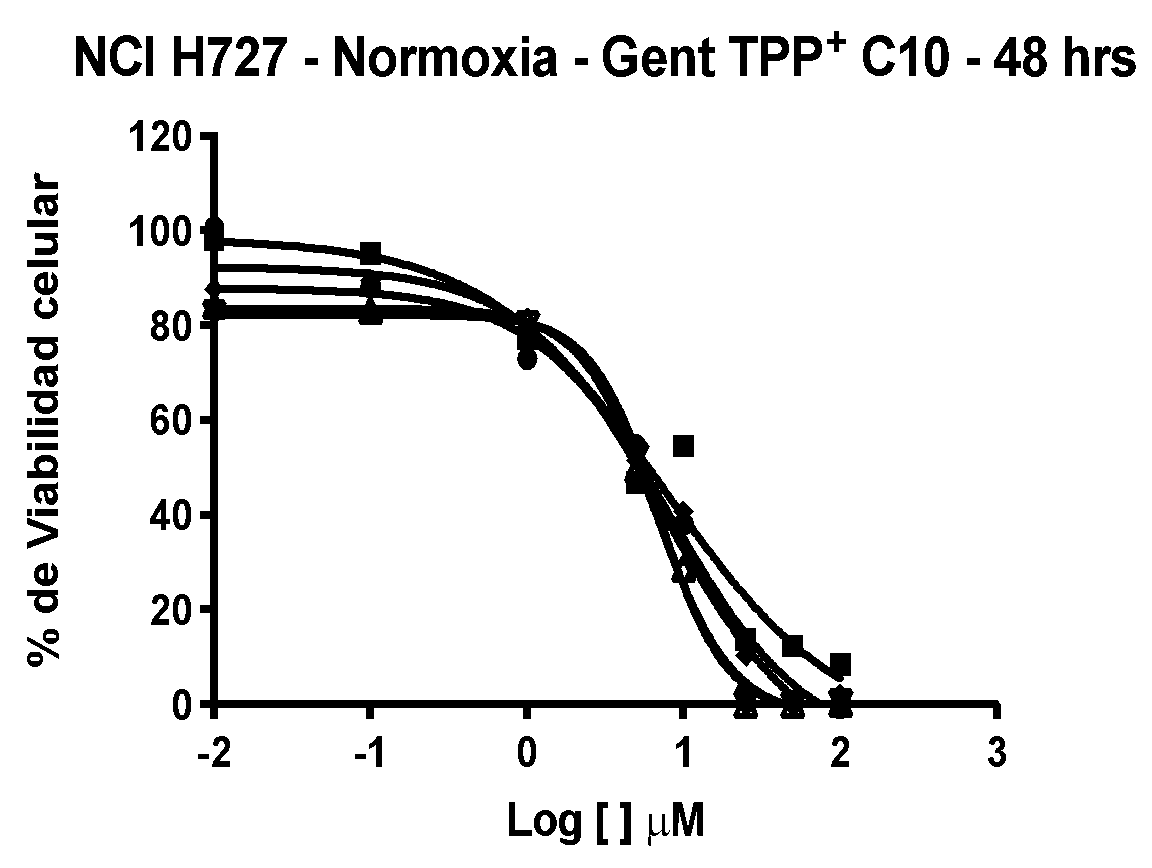** | **C**  **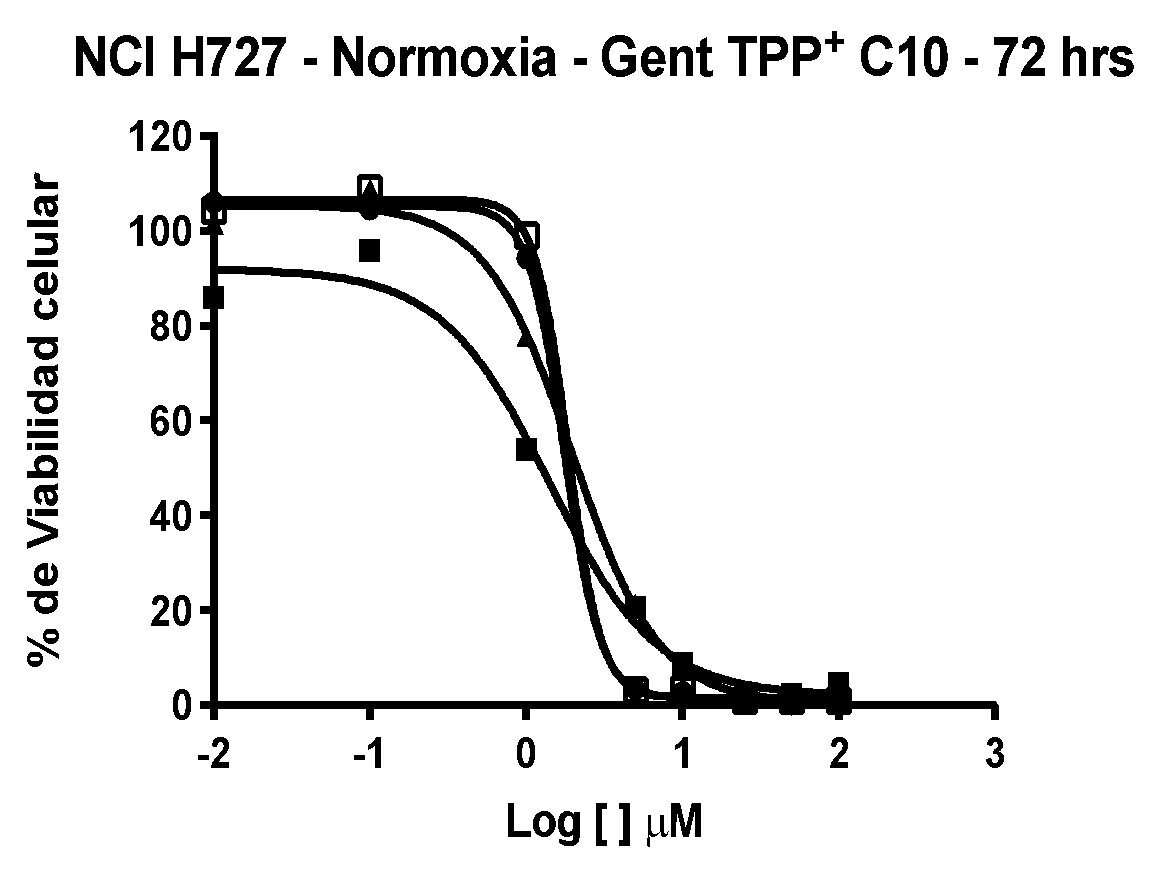** |
| **IC_50_** | 10,48 ± 1,64 *μM* | 7,42 ± 0,71 *μM* | 1,75 ± 0,24 *μM* |
| **Hypoxic** | **D**  **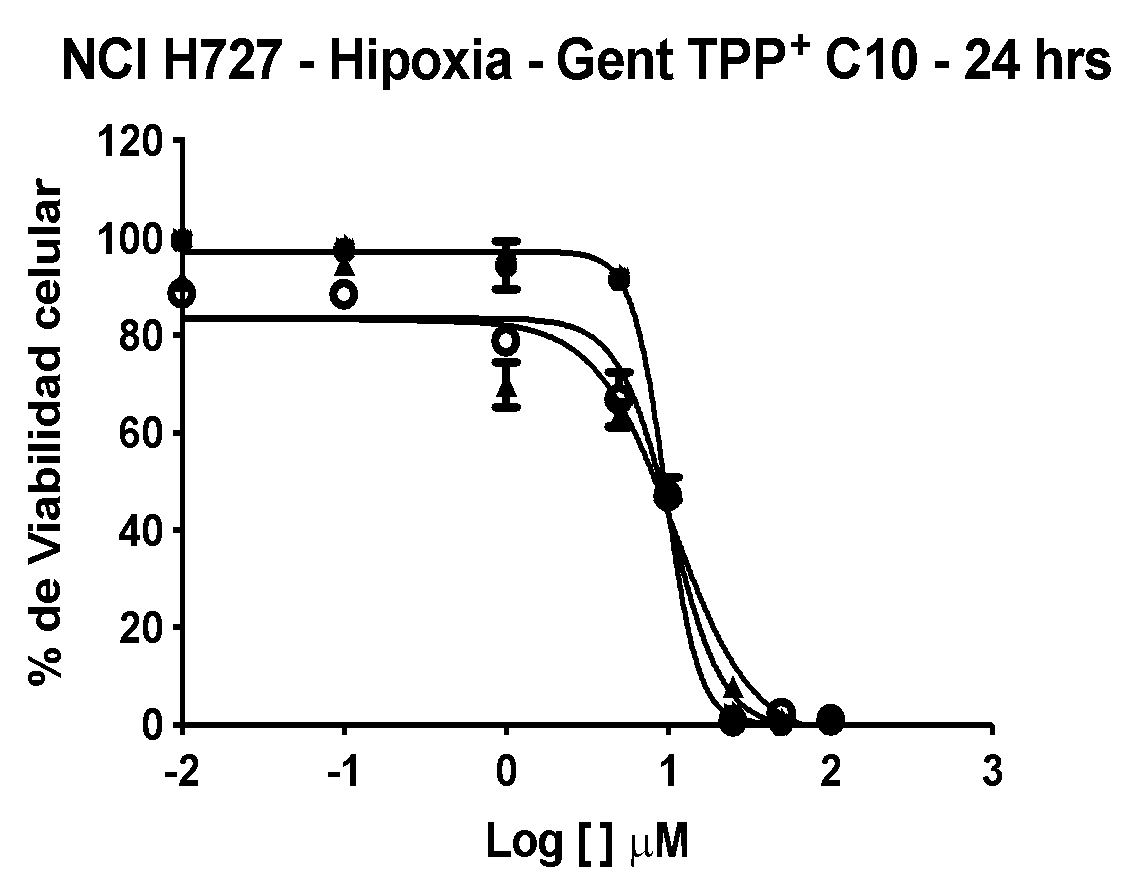** | **E**  **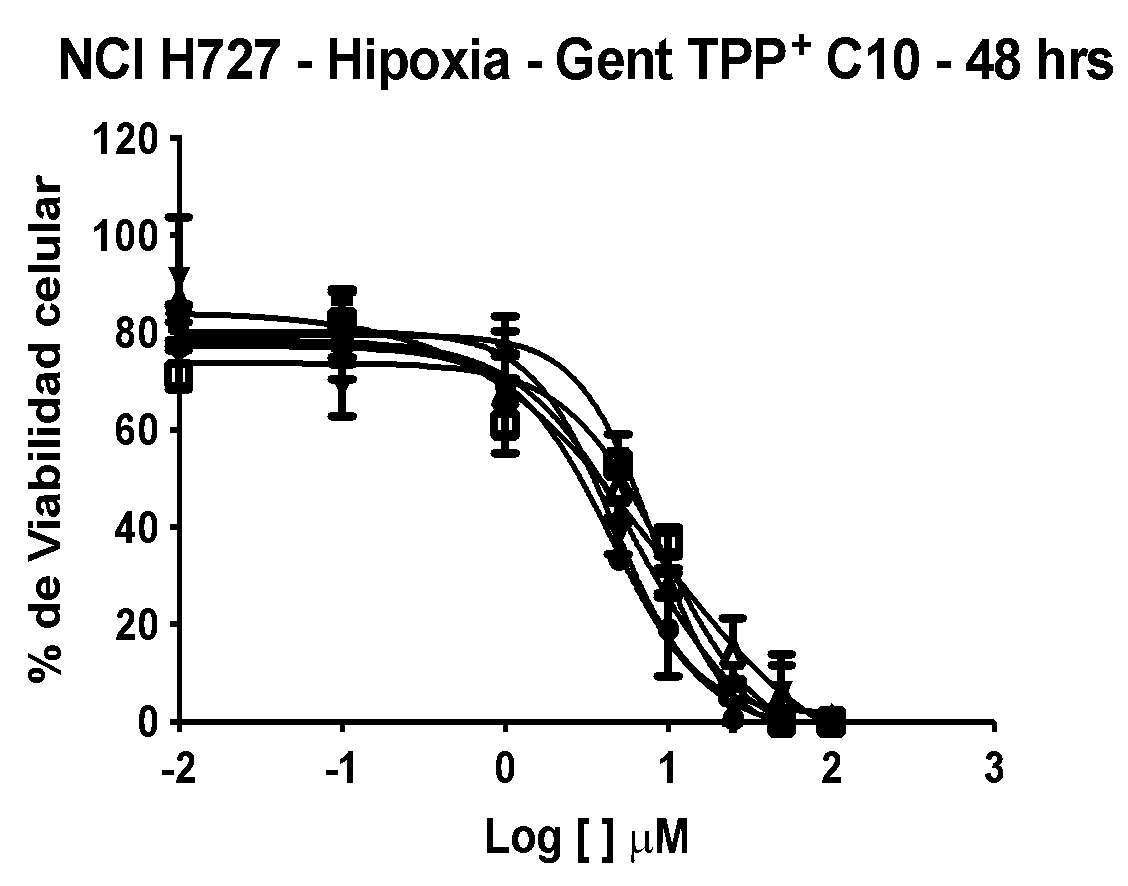** | **F**  **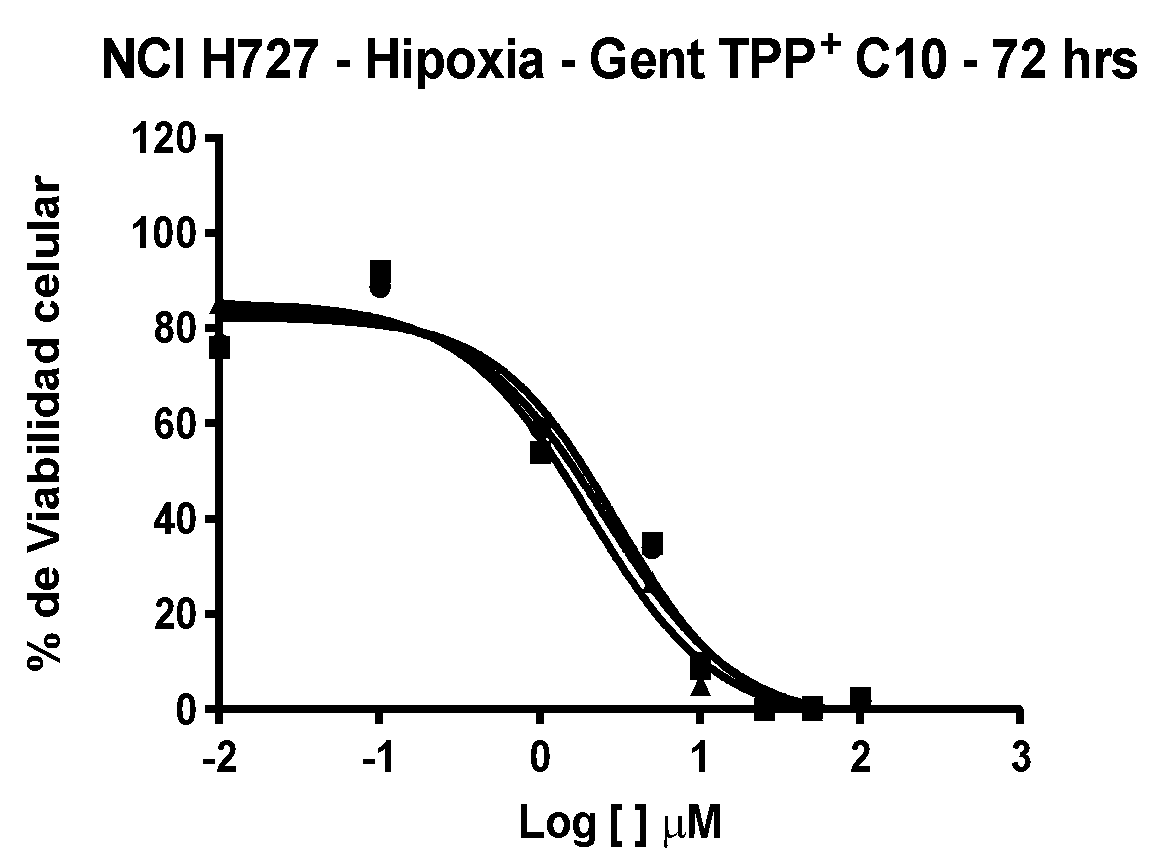** |
| **IC_50_** | 10,32 ± 0,65 *μM* | 6,98 ± 2,11 *μM* | 2,52 ± 0,49 *μM* |

***Figure. S4.*** Cytotoxic effect of GATPP^+^C_10_ on NCI H727 cell line monolayer cultures under normoxic and hypoxic conditions. Graphical representation of the effect of the compound GATPP^+^C_10_ on cell viability as a function of the logarithm of the concentration. The cytotoxic effect is observed at 24 h (A and D), 48 h (B and E) and 72 h (C and F). Data obtained by averaging at least 3 independent experiments. The averages of the IC_50_ ± SD are shown.

**Cell line NCI–H1299**

|  | **24 h** | **48 h** | **72 h** |
| --- | --- | --- | --- |
| **Normoxic** | **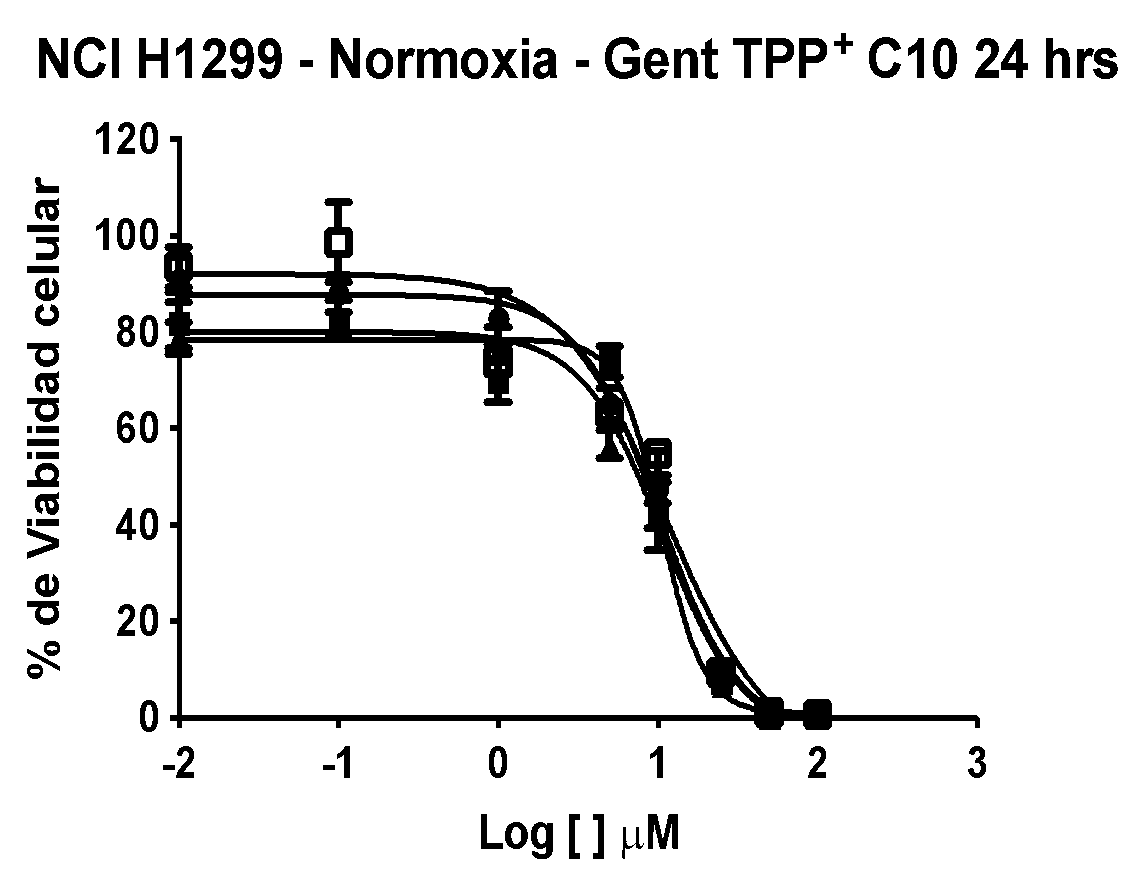**  **A** | **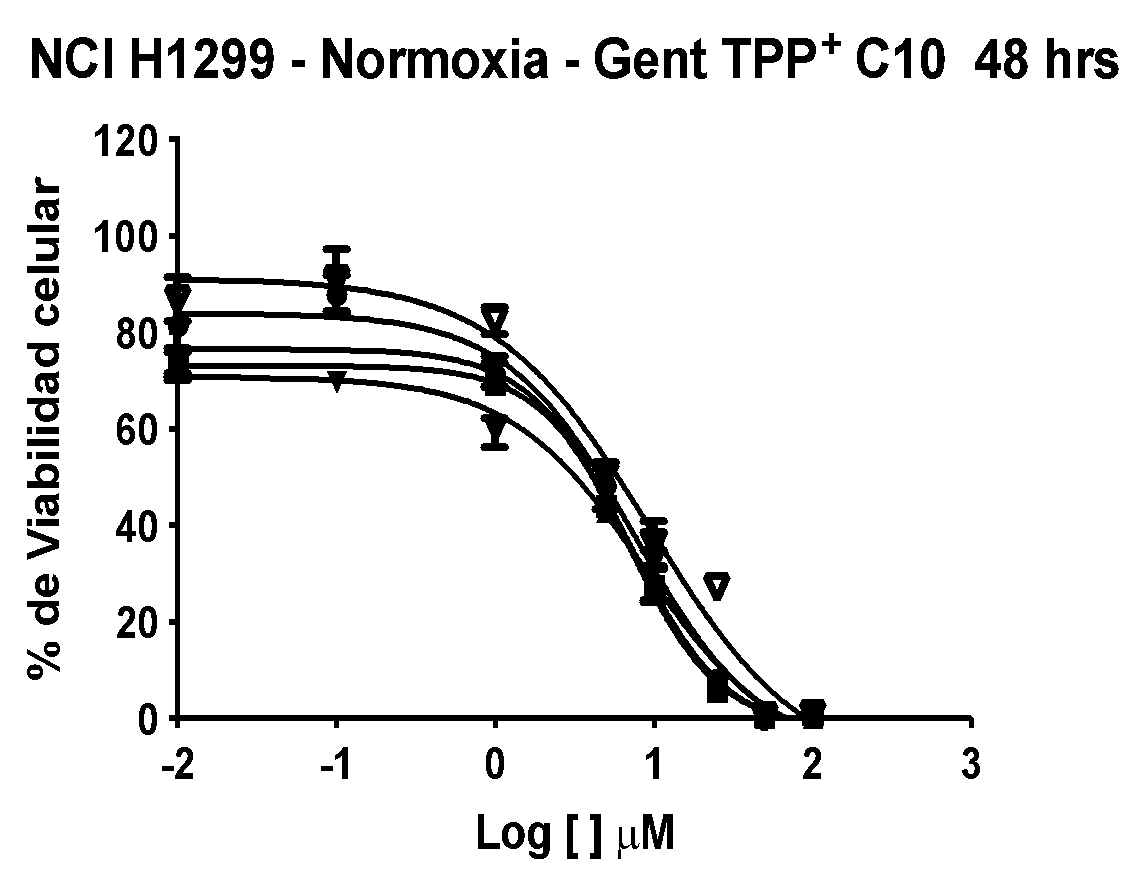**  **B** | **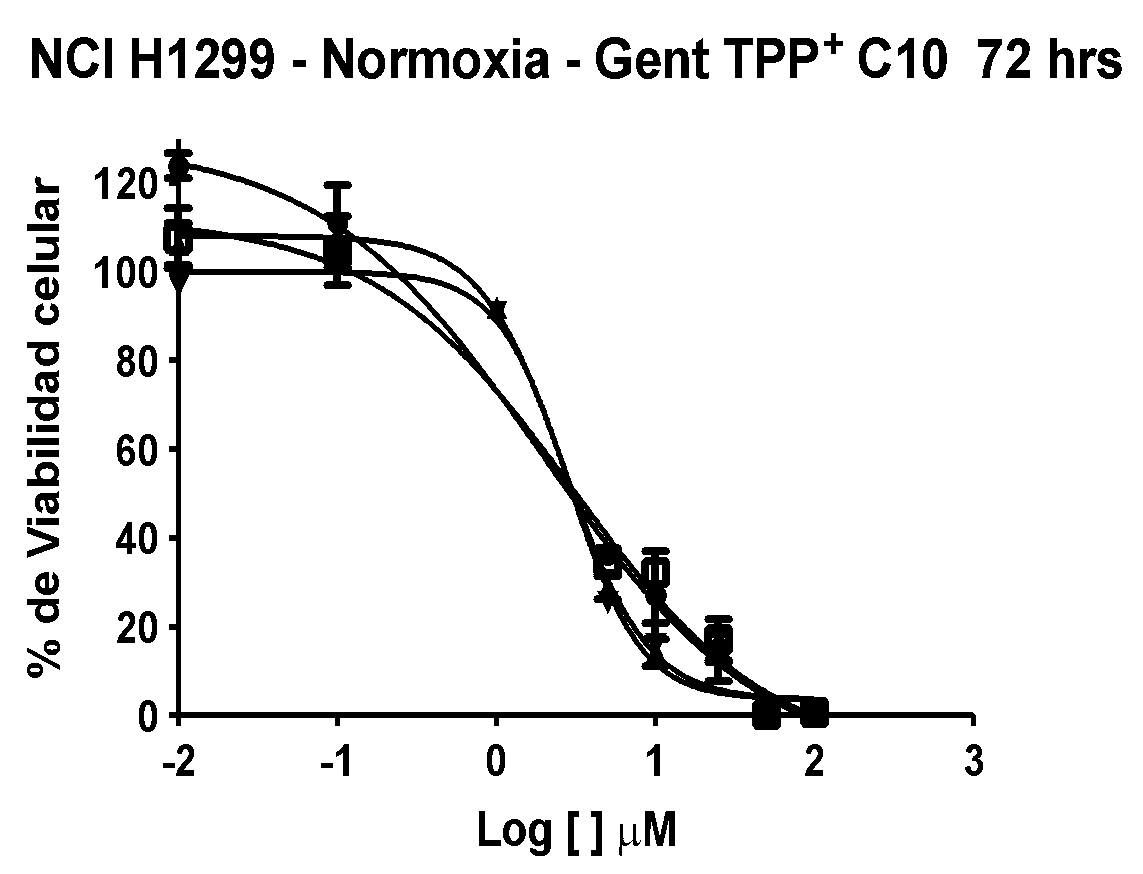**  **C** |
| **IC_50_** | 10,66 ± 0,42 *μM* | 7,92 ± 1,1 *μM* | 2,65 ± 0,48 *μM* |
| **Hypoxic** | **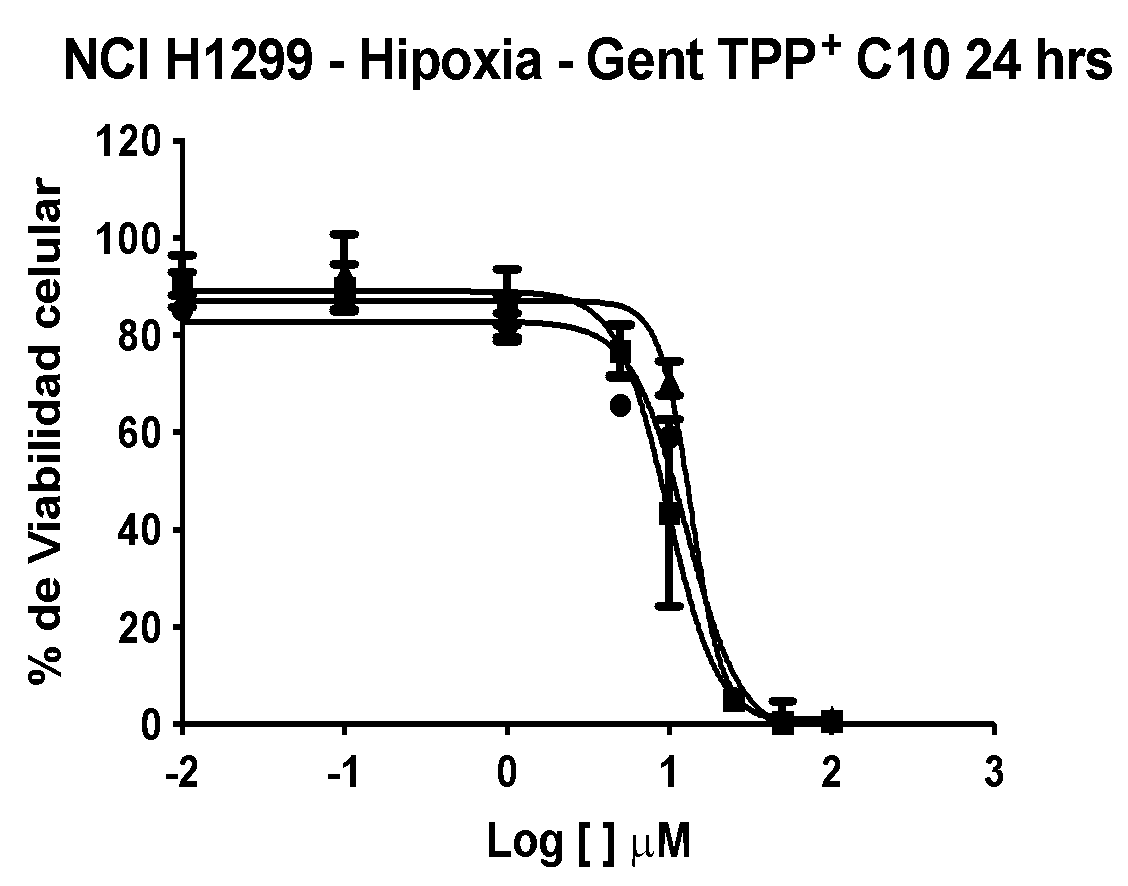**  **D** | **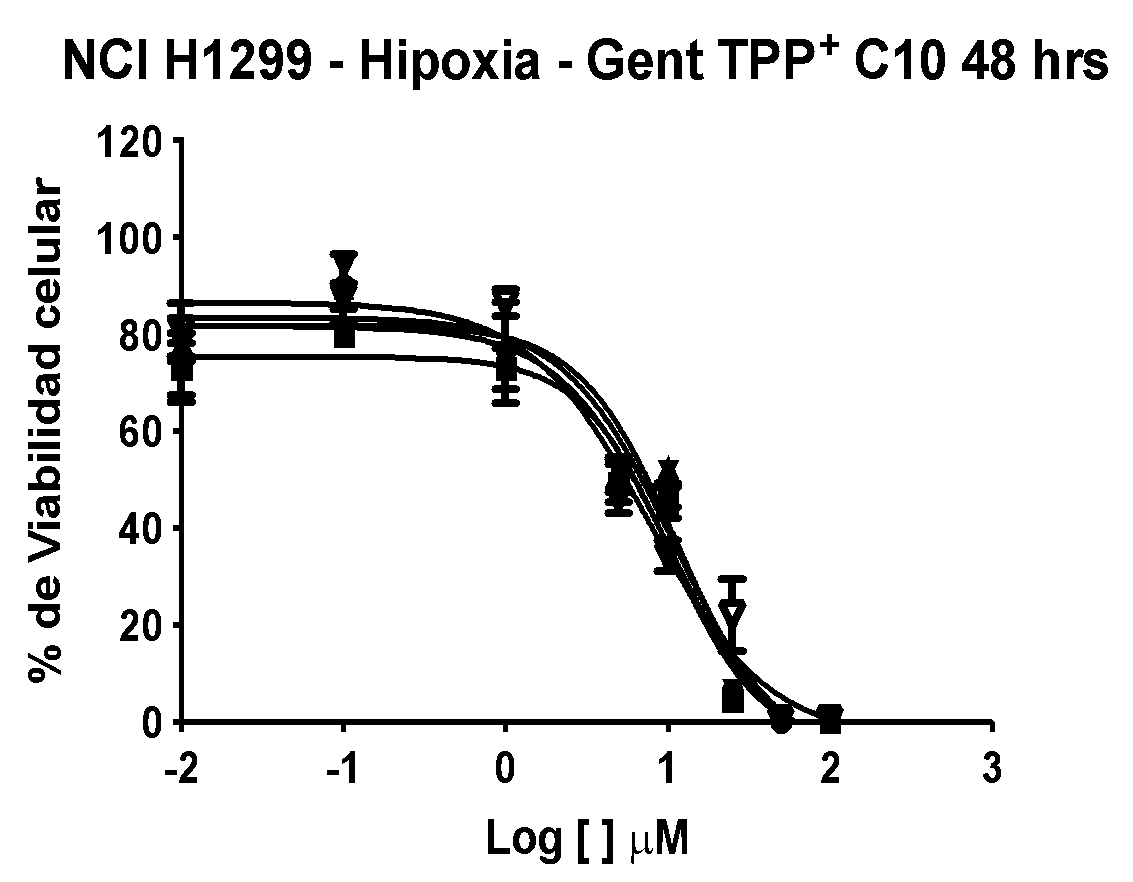**  **E** | **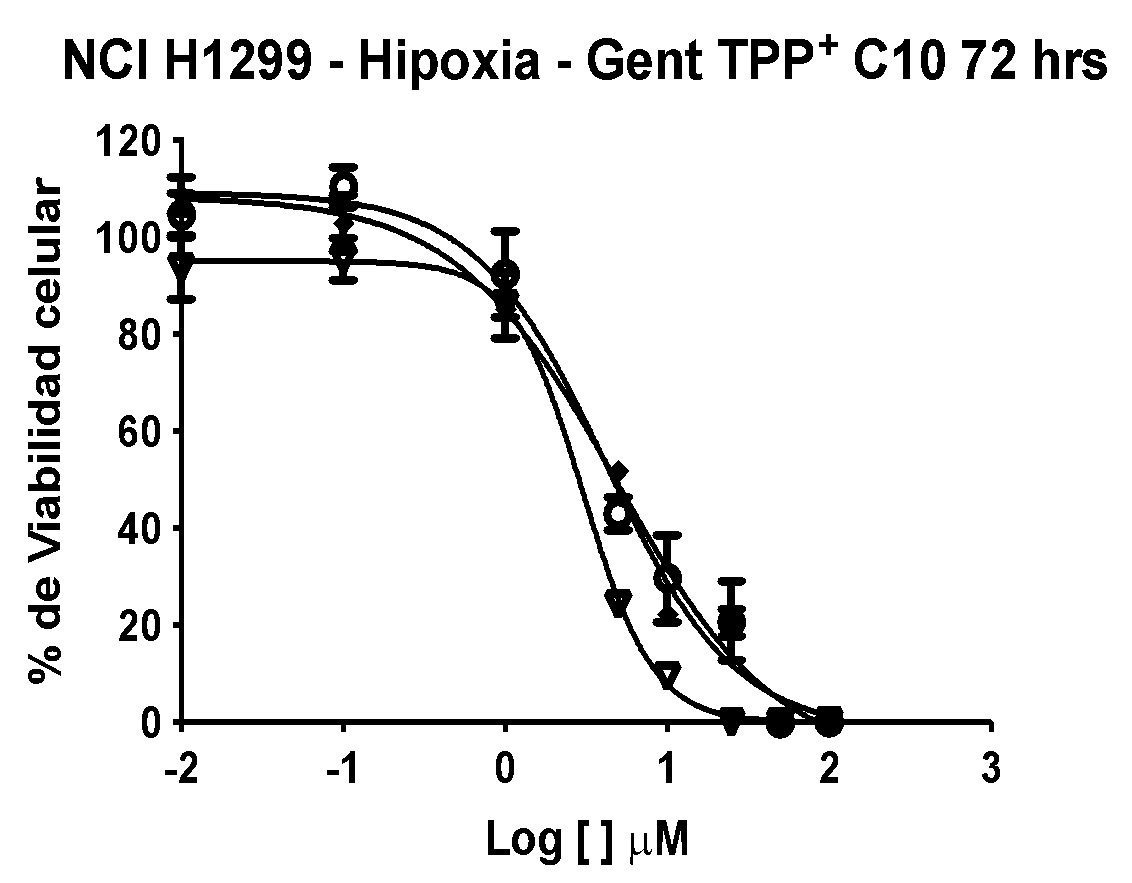**  **F** |
| **IC_50_** | 12,08 ± 2,03 *μM* | 9,96 ± 1,42 *μM* | 4,01 ± 0,92 *μM* |

***Figure. S5.*** Cytotoxic effect of GA-TPP^+^C_10_ on NCI H1299 cell line monolayer cultures under normoxic and hypoxic conditions. Graphical representation of the effect of the compound GA-TPP^+^C_10_ on cell viability as a function of the logarithm of the concentration. The cytotoxic effect is observed at 24 h (A and D), 48 h (B and E) and 72 h (C and F). Data obtained by averaging 5 independent experiments. The averages of the IC_50_ ± SD are shown.

**Lung Fibroblast**

|  | **24 h** | **48 h** | **72 h** |
| --- | --- | --- | --- |
| **Normoxic** | **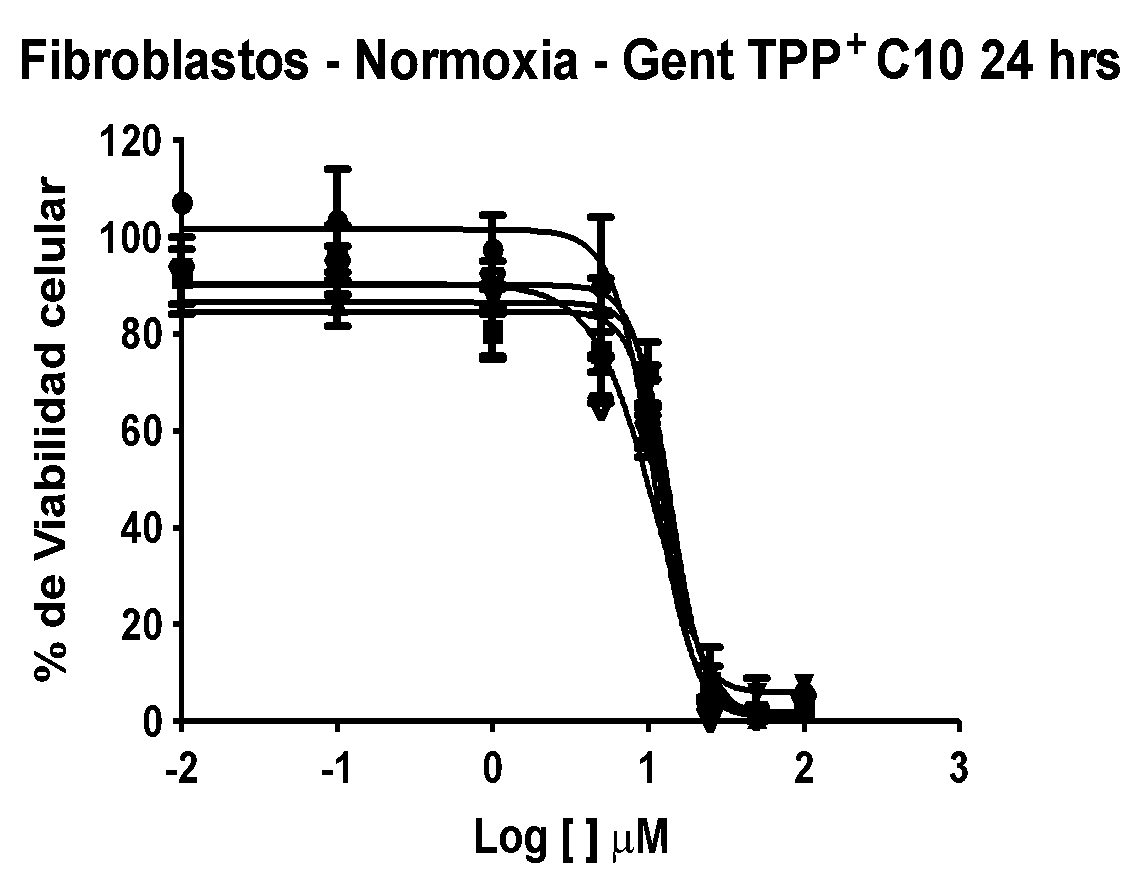**  **A** | **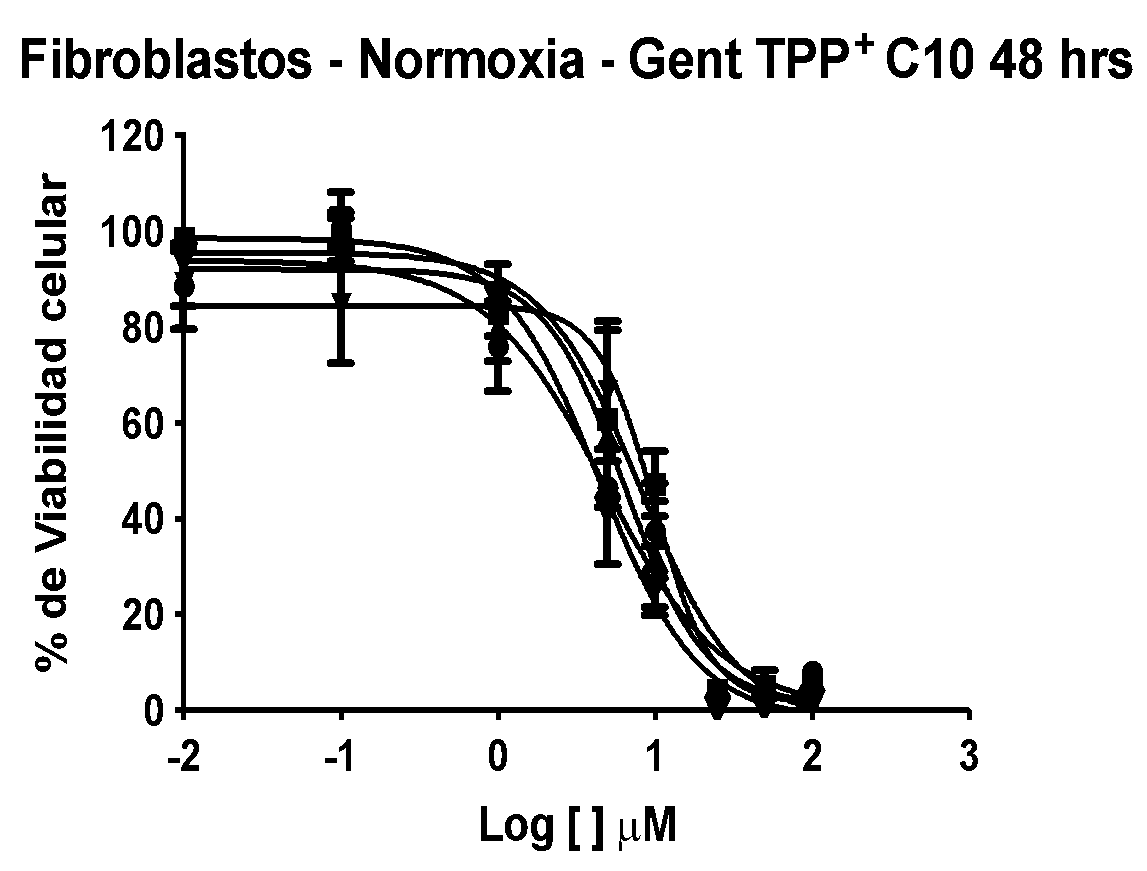**  **B** | **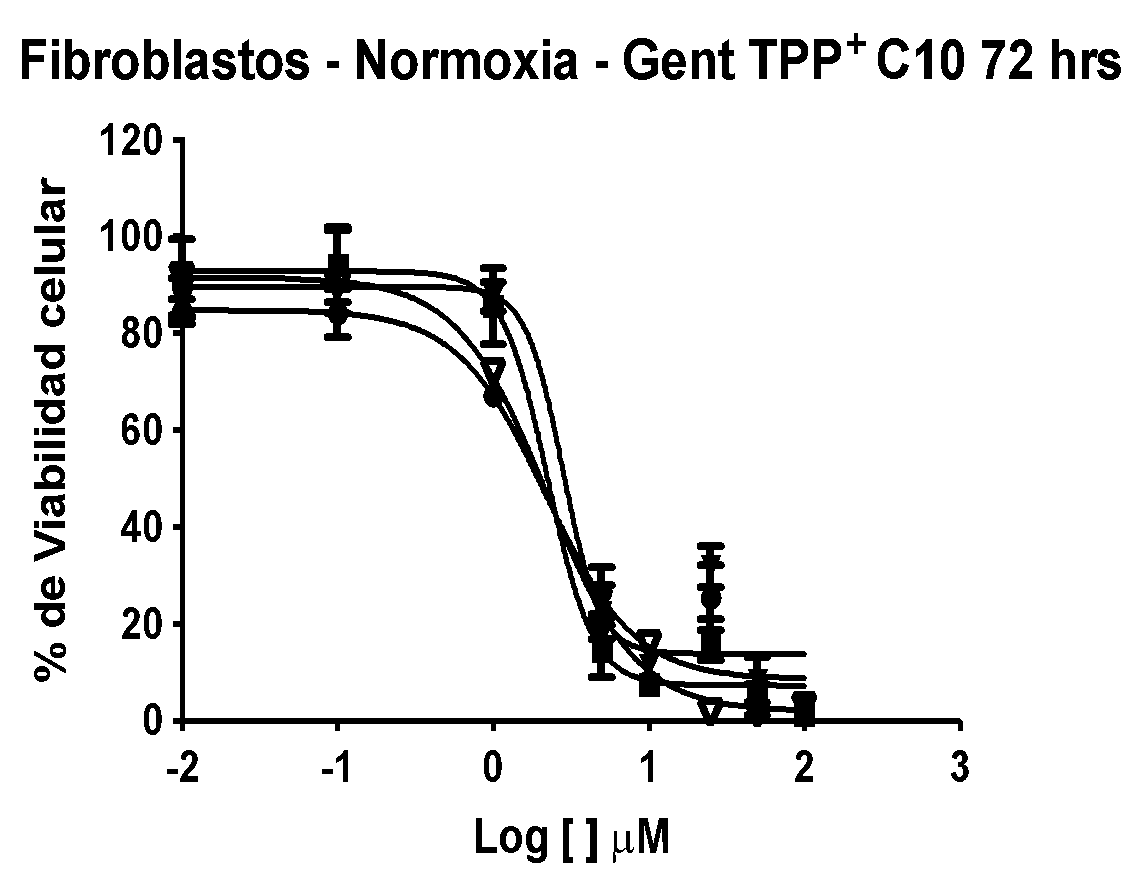**  **C** |
| **IC_50_** | 12,34 ± 1,43 *μM* | 6,73 ± 2,3 *μM* | 2,37 ± 0,3 *μM* |

***Figure. S6.*** Cytotoxic effect of GA-TPP^+^C_10_ on monolayer cultures of lung fibroblast cell line under normoxic and hypoxic conditions. Graphical representation of the effect of the compound GA-TPP^+^C_10_ on cell viability as a function of the logarithm of the concentration. The cytotoxic effect is observed at 24 h (A), 48 h (B) and 72 h (C). Data obtained by averaging 5 independent experiments. The averages of the IC_50_ ± SD are shown.

**c. Doxycycline**

**Cell line NCI–H727**

|  | **24 h** | **48 h** | **72 h**  **C** |
| --- | --- | --- | --- |
| **Normoxic** | **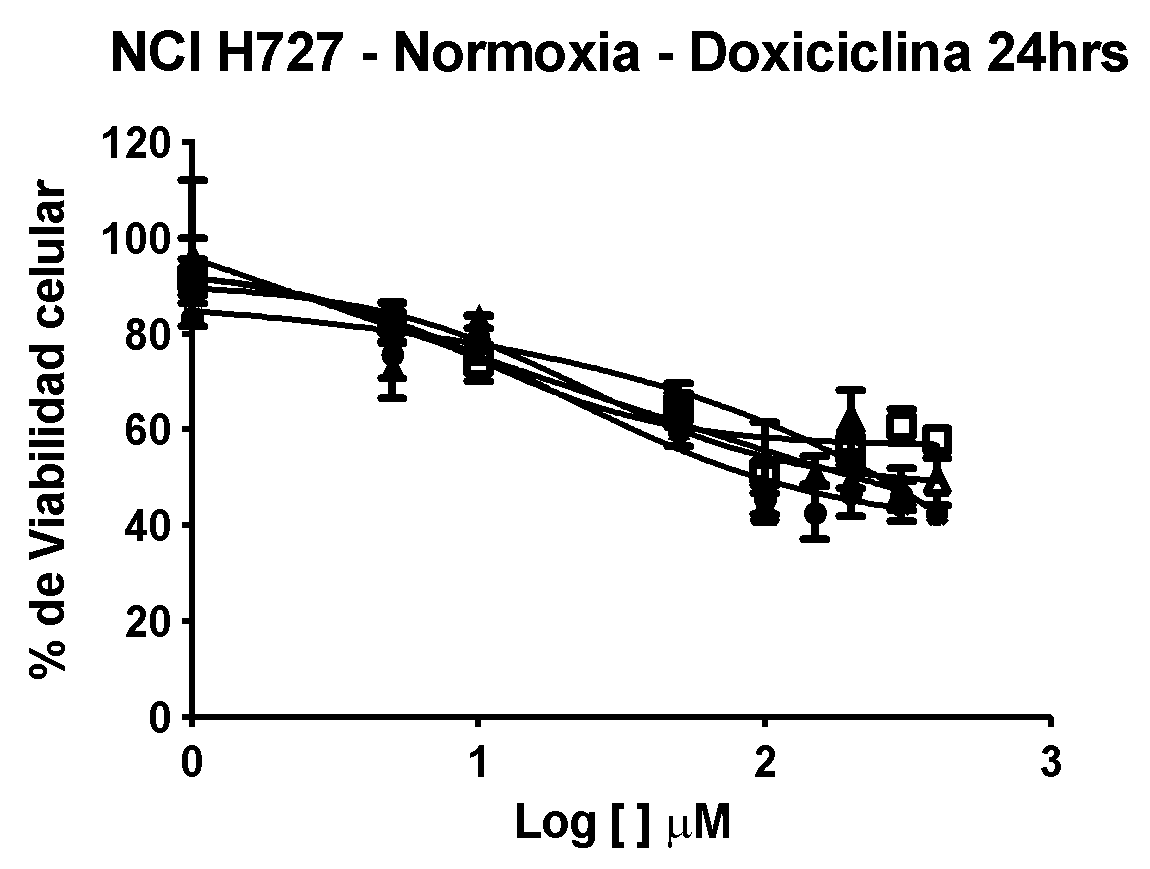**  **A** | **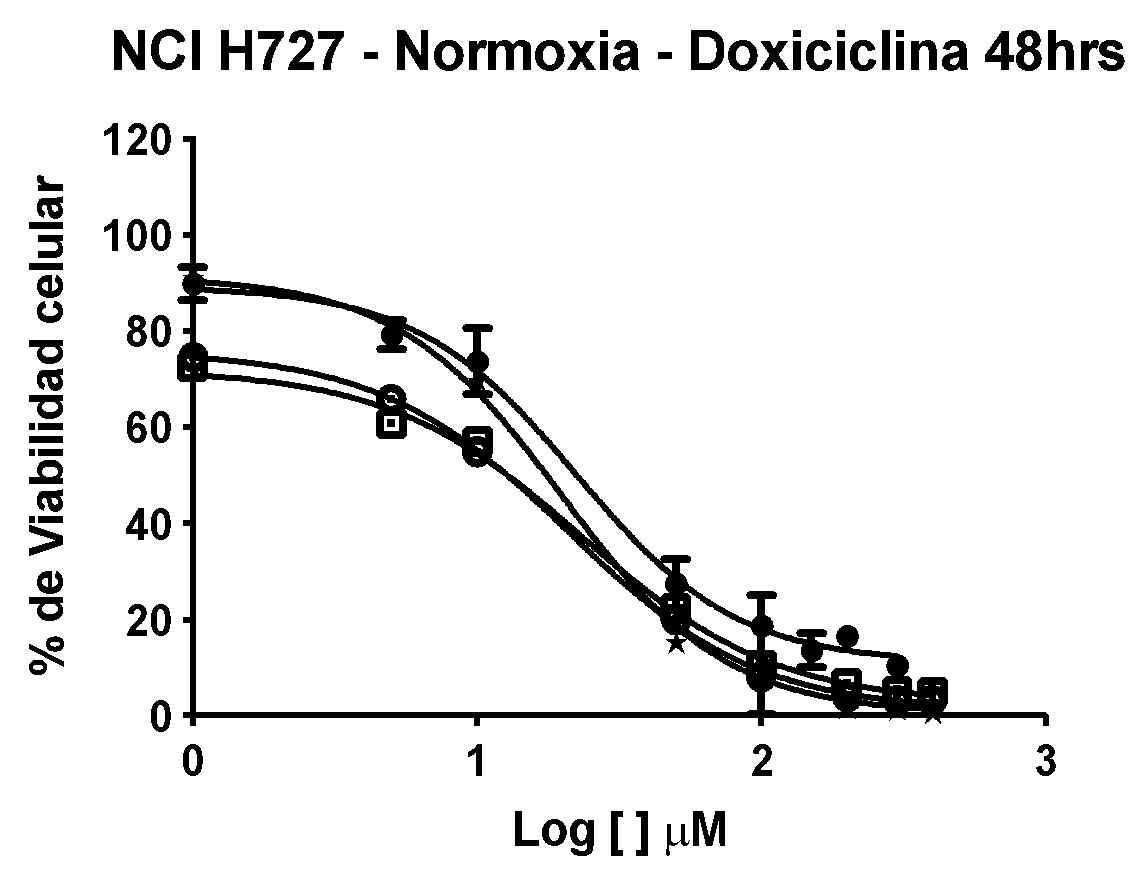**  **B** | **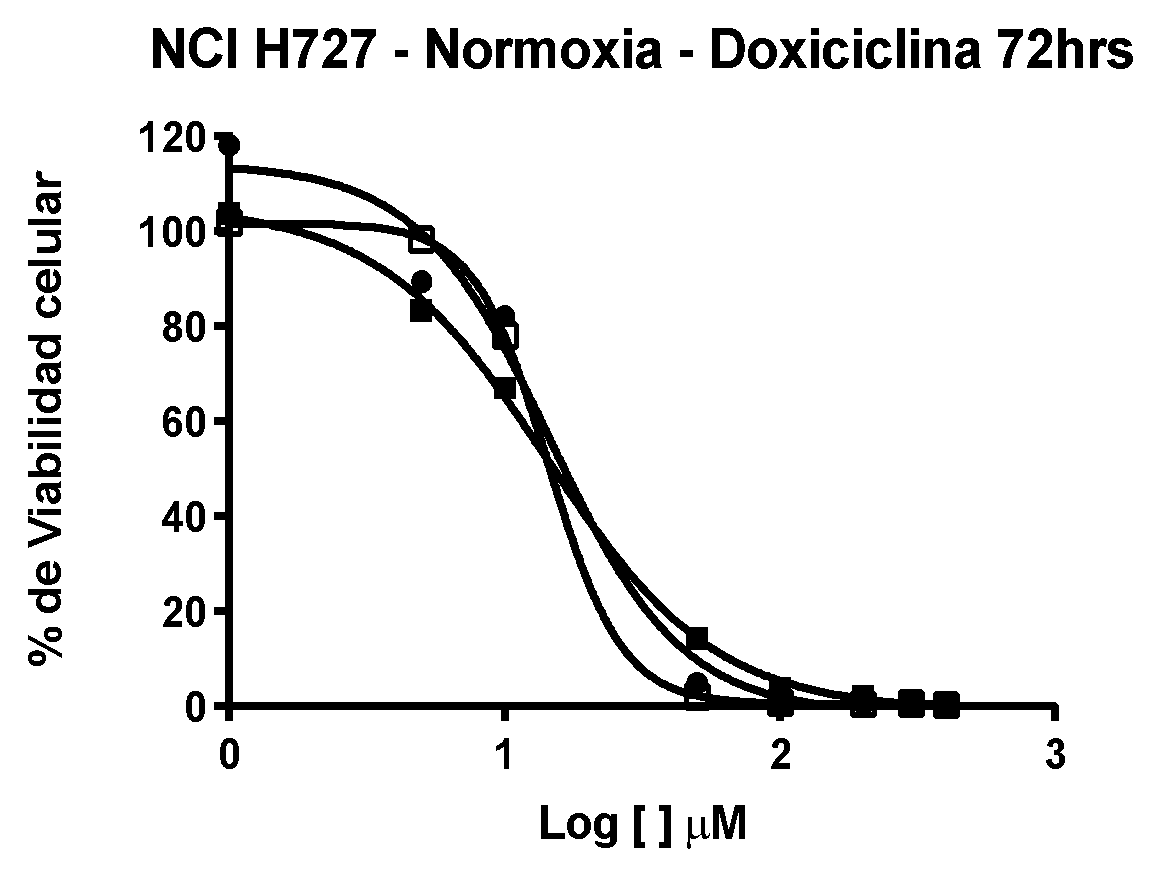** |
| **IC_50_** | >100 *μM* | 21,63 ± 1,56 *μM* | 14,42 ± 0,18 *μM* |
| **Hypoxia** | **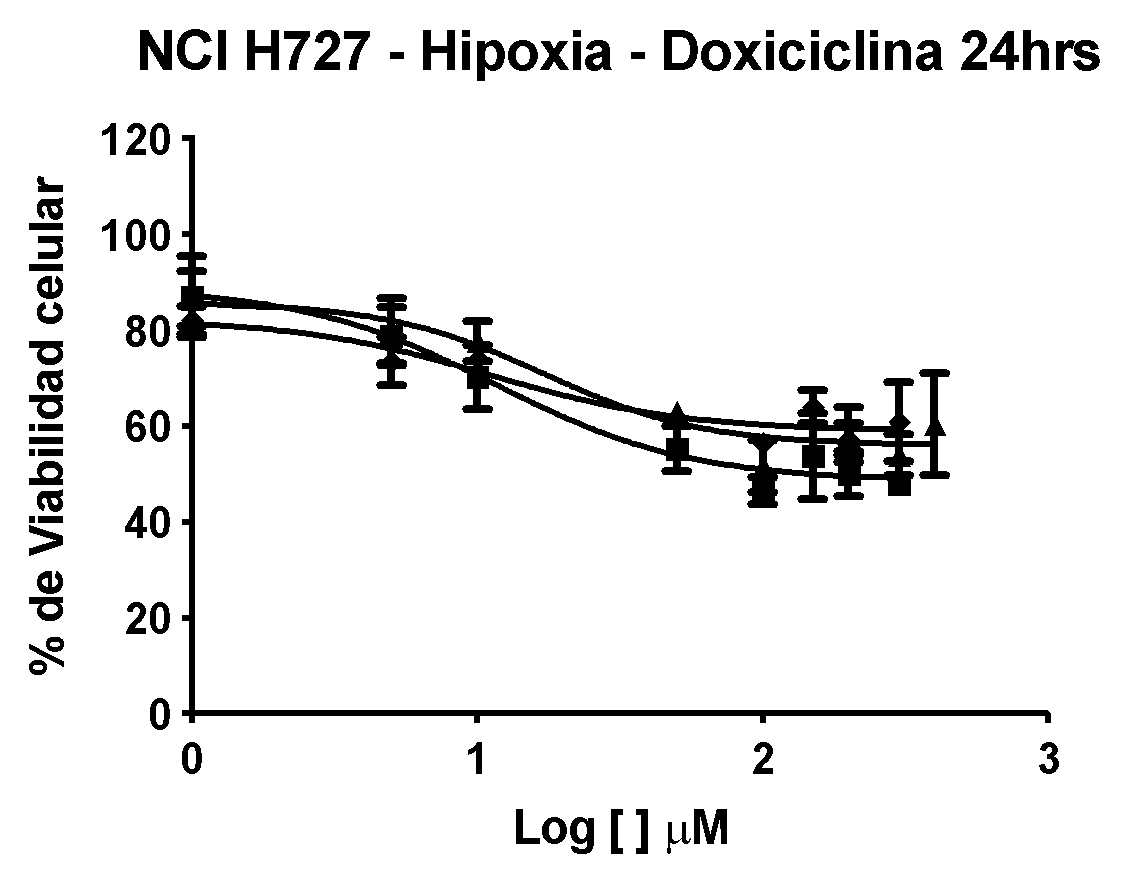**  **D** | **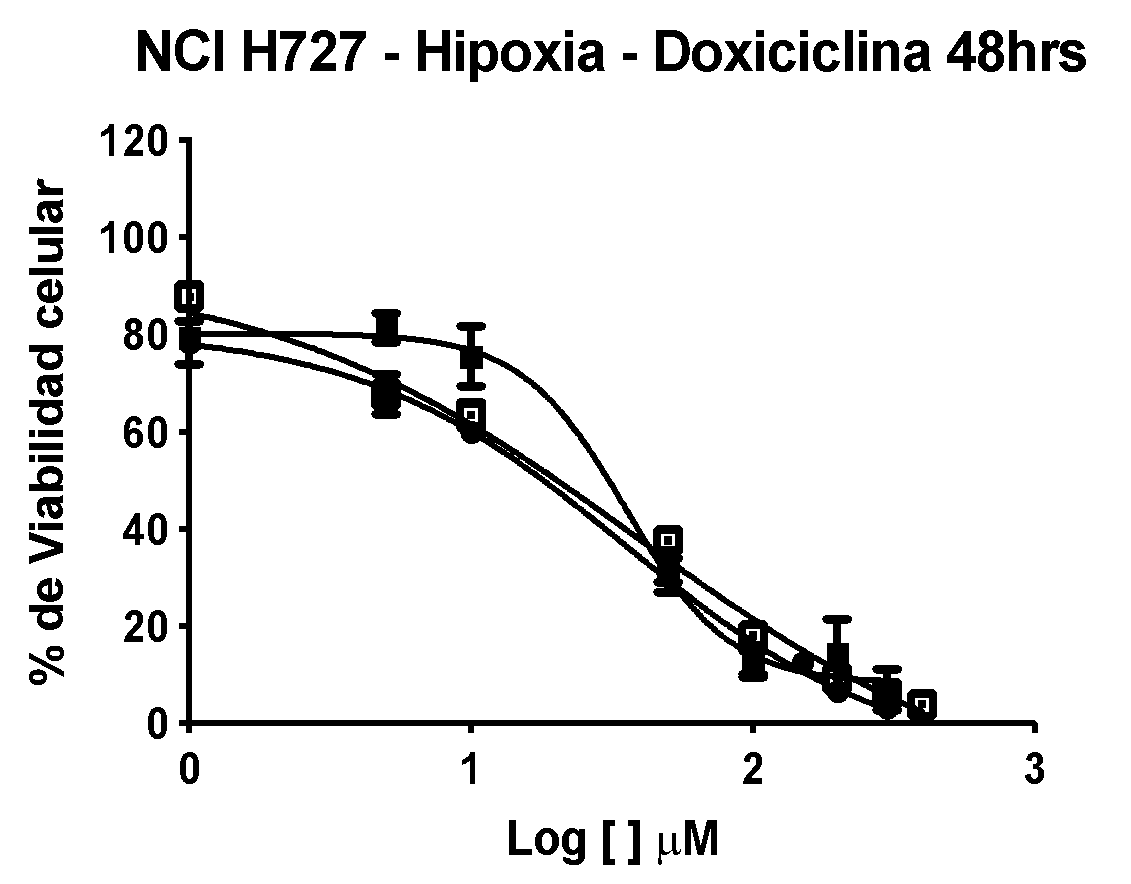**  **E** | **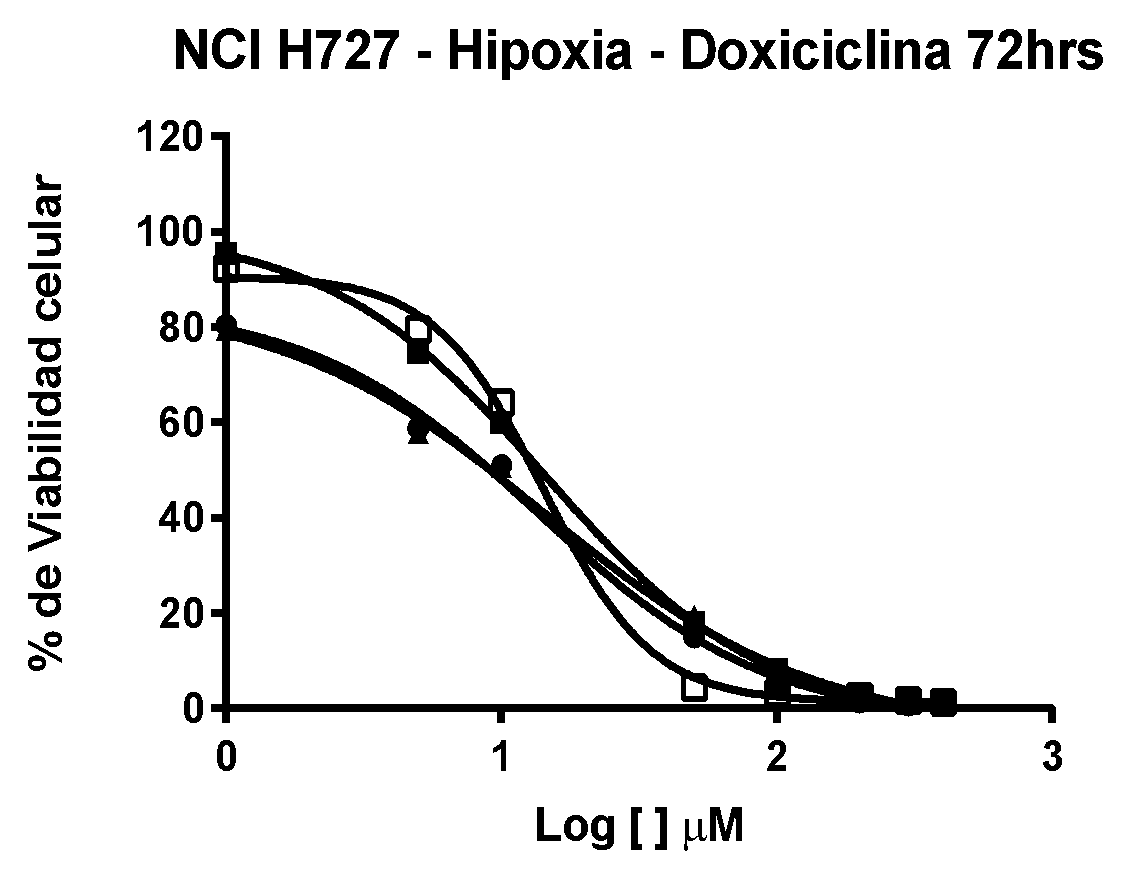**  **F** |
| **IC_50_** | >300 *μM* | 36,6 ± 2,11 *μM* | 14,22 ± 0,44 *μM* |

***Figure. S7.*** Cytotoxic effect of Doxycycline on NCI H727 cell line monolayer cultures under normoxic and hypoxic conditions. Graphical representation of the effect of the compound Doxycycline on cell viability as a function of the logarithm of the concentration. The cytotoxic effect is observed at 24 h (A and D), 48 h (B and E) and 72 h (C and F). Data obtained by averaging at least 4 independent experiments. The averages of the IC_50_ ± SD are shown.

**Cell line NCI–H1299**

|  | **24 h** | **48 h** | **72 h** |
| --- | --- | --- | --- |
| **Normoxic** | **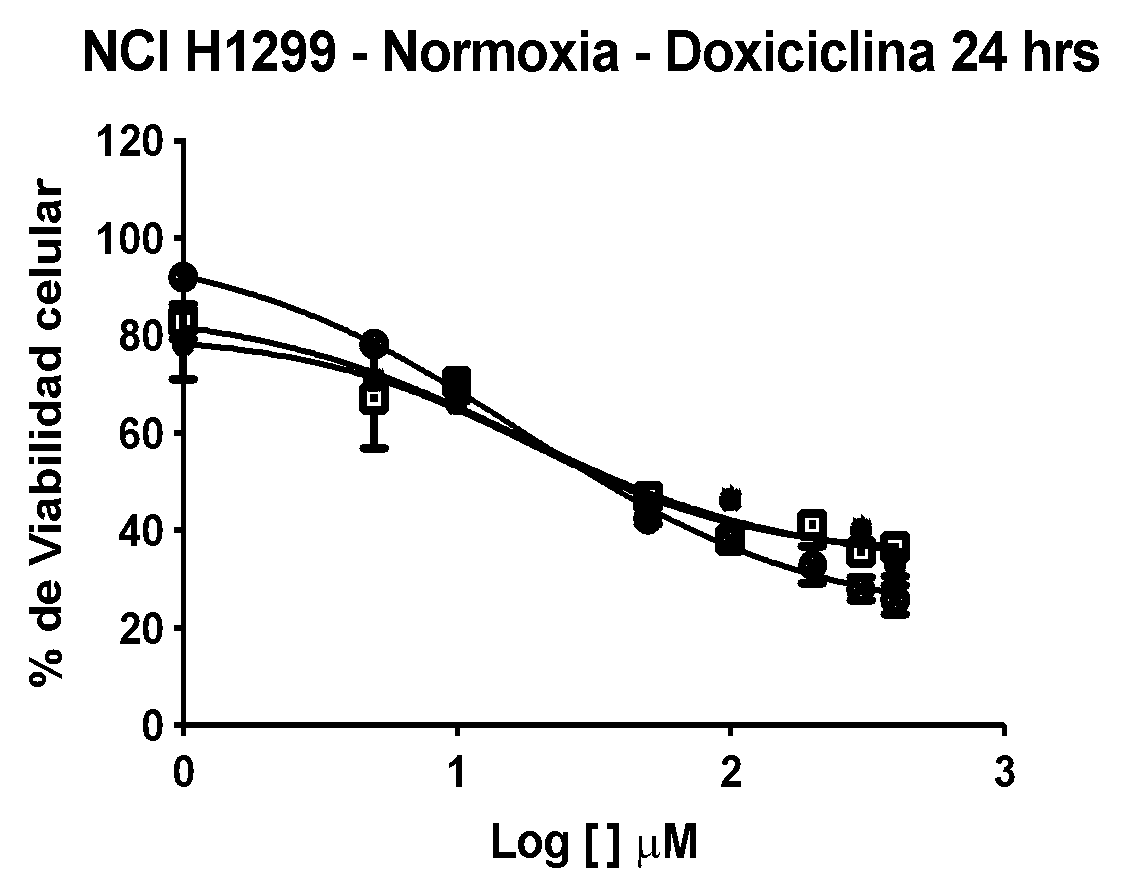**  **A** | **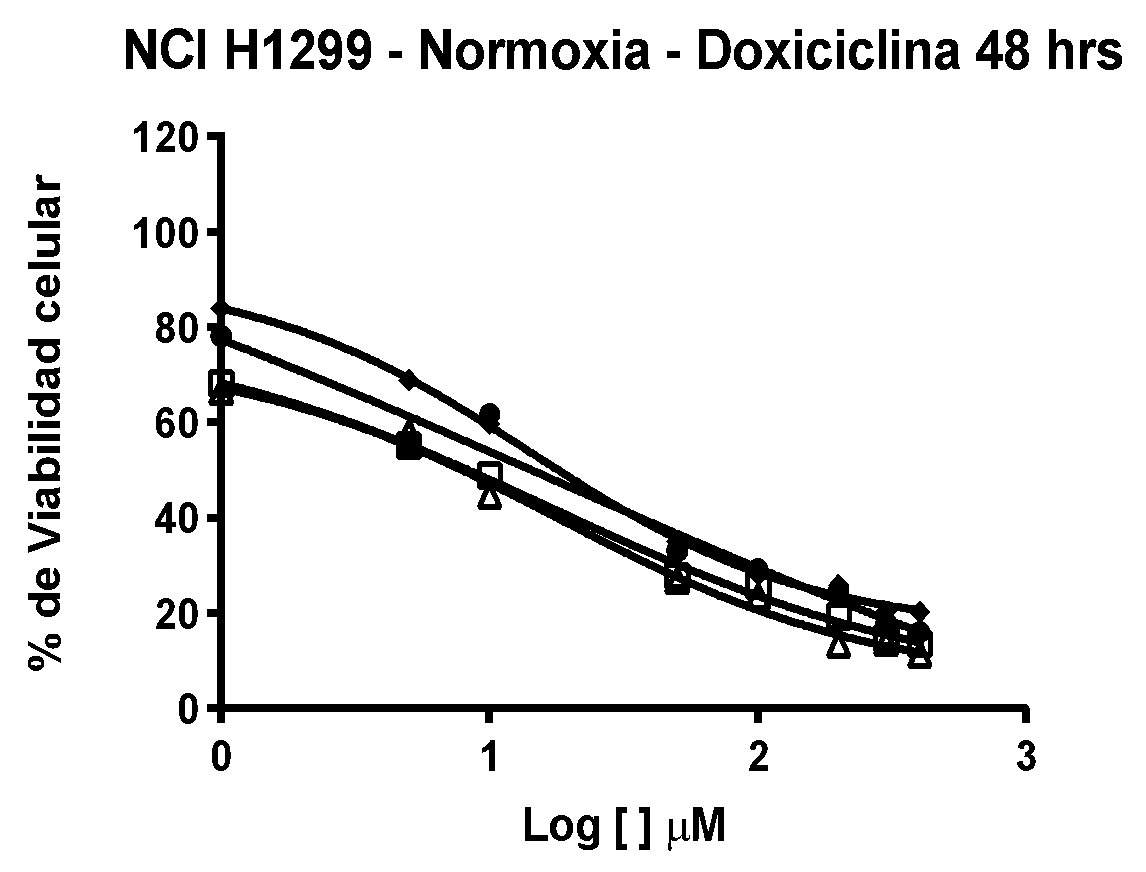**  **B** | **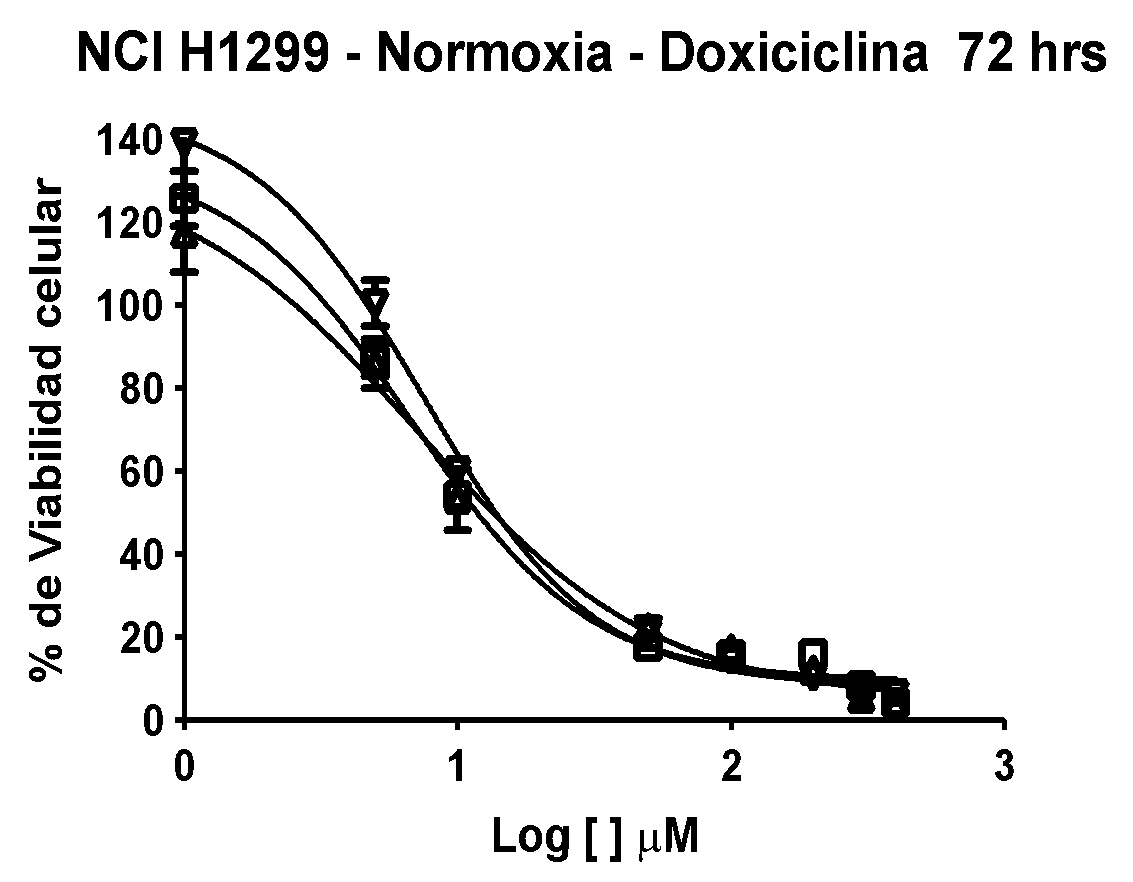**  **C** |
| **IC_50_** | 17,76 ± 0,87 *μM* | 16,39 ± 1,63 *μM* | 7,16 ± 0,35 *μM* |
| **Hypoxia** | **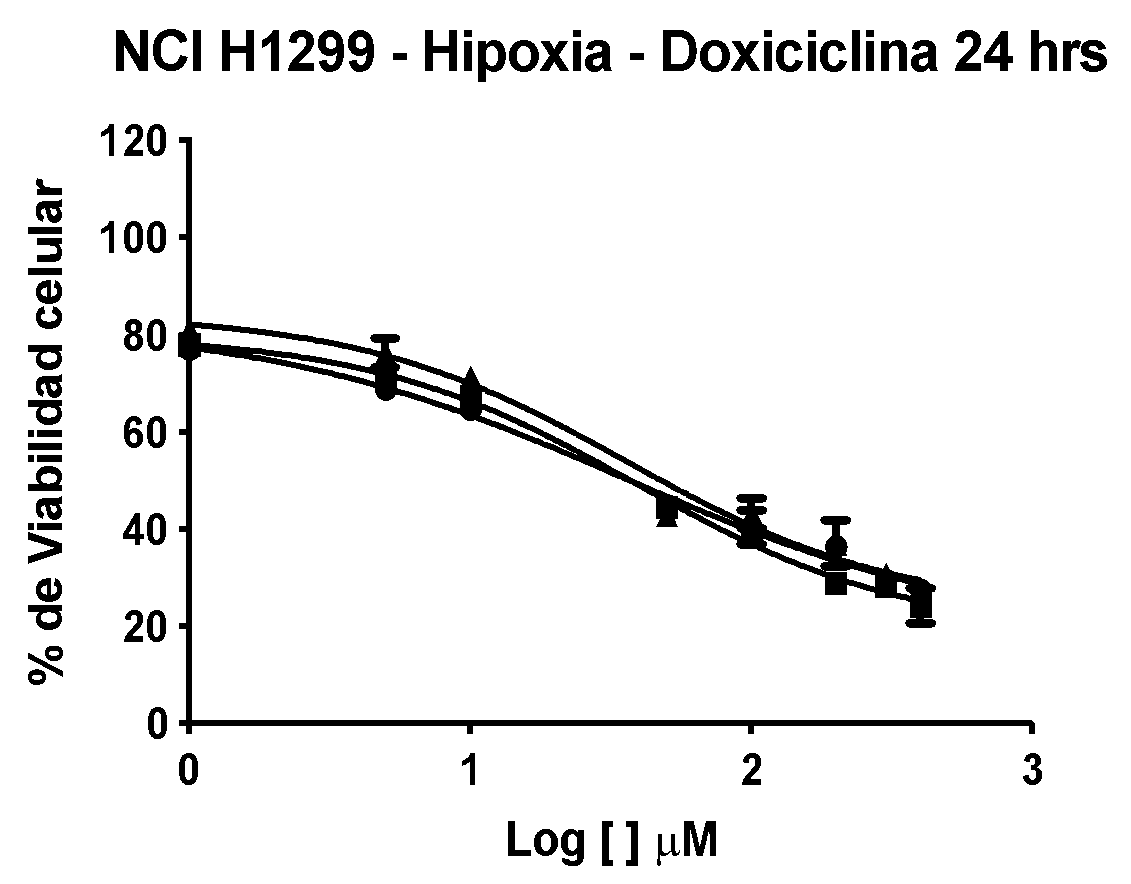**  **D** | **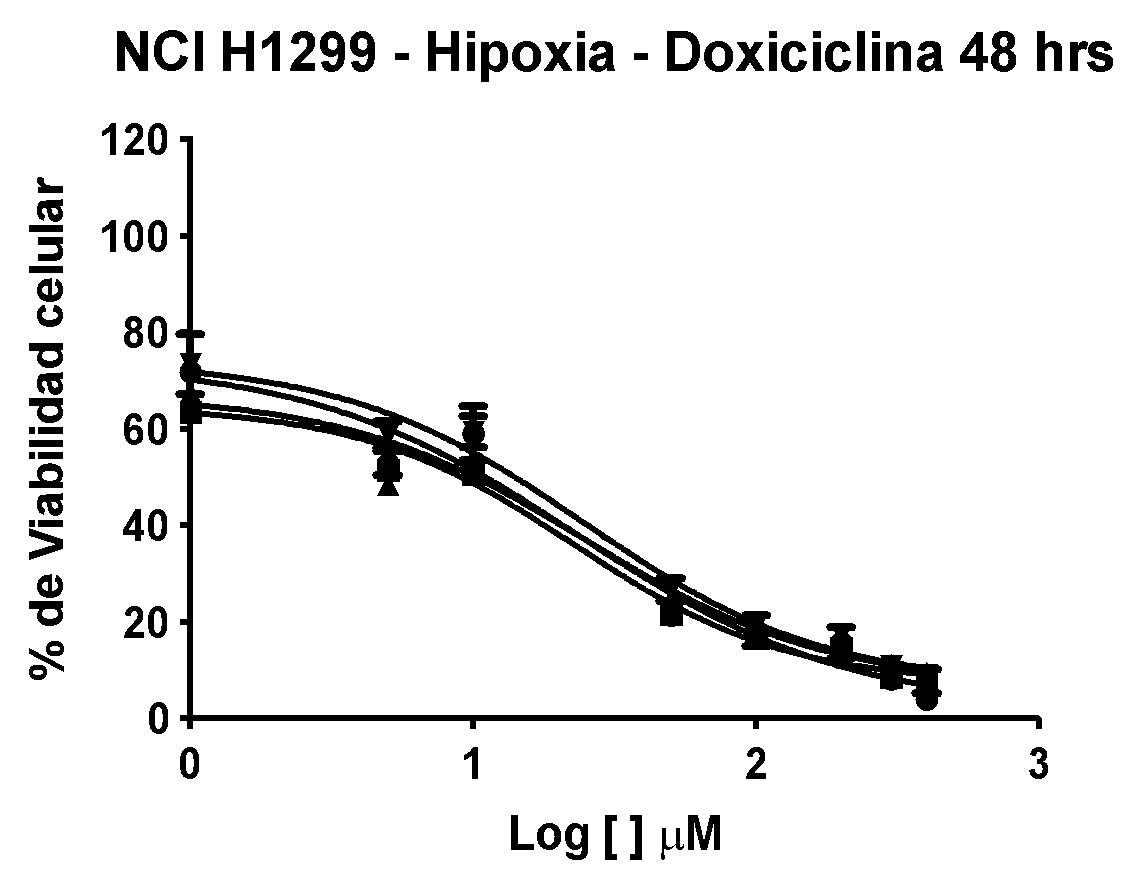**  **E** | **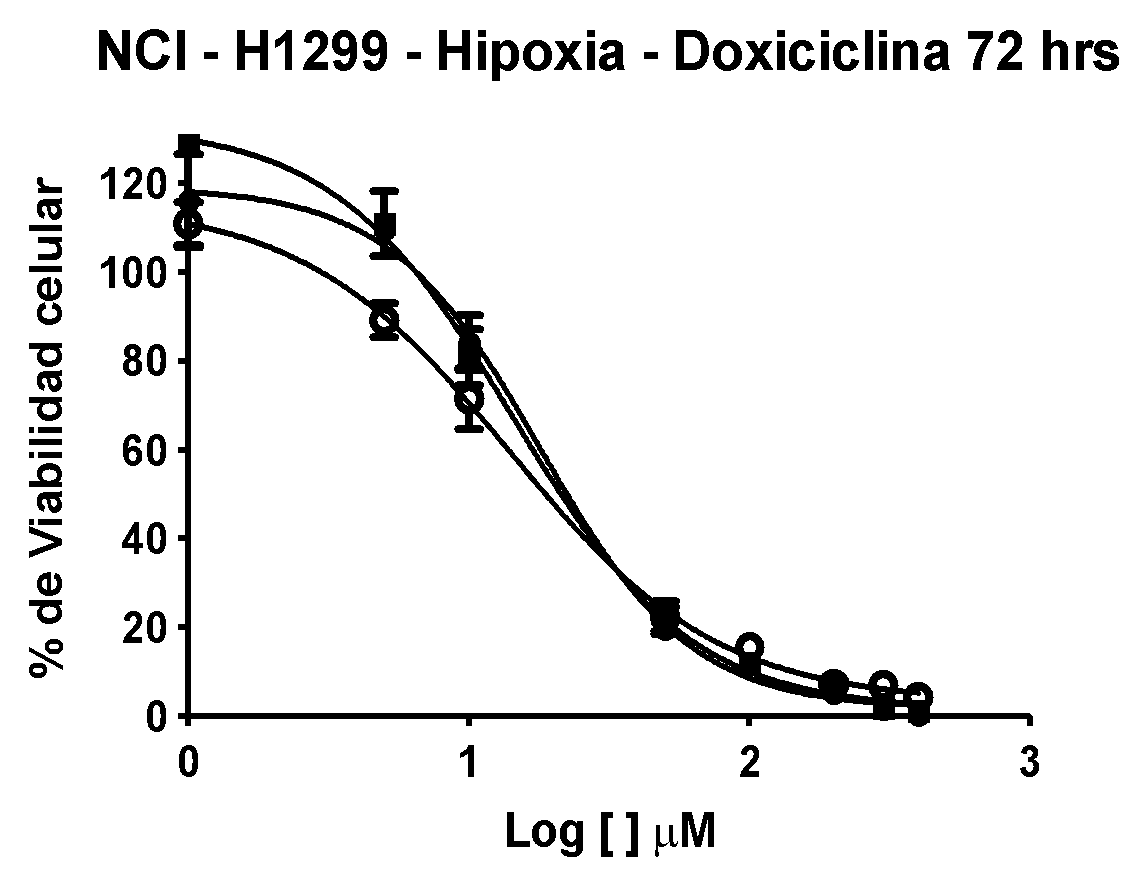**  **F** |
| **IC_50_** | 37,2 ± 1,64 *μM* | 24,09 ± 1,02 *μM* | 15,47 ± 2,13 *μM* |

***Figure. S8.*** Cytotoxic effect of Doxycycline on NCI H1299 cell line monolayer cultures under normoxic and hypoxic conditions. Graphical representation of the effect of the compound Doxycycline on cell viability as a function of the logarithm of the concentration. The cytotoxic effect is observed at 24 h (A and D), 48 h (B and E) and 72 h (C and F). Data obtained by averaging at least 3 independent experiments. The averages of the IC_50_ ± SD are shown.

**Lung fibroblast**

|  | **24 h** | **48 h** | **72 h** |
| --- | --- | --- | --- |
| **Normoxic** | **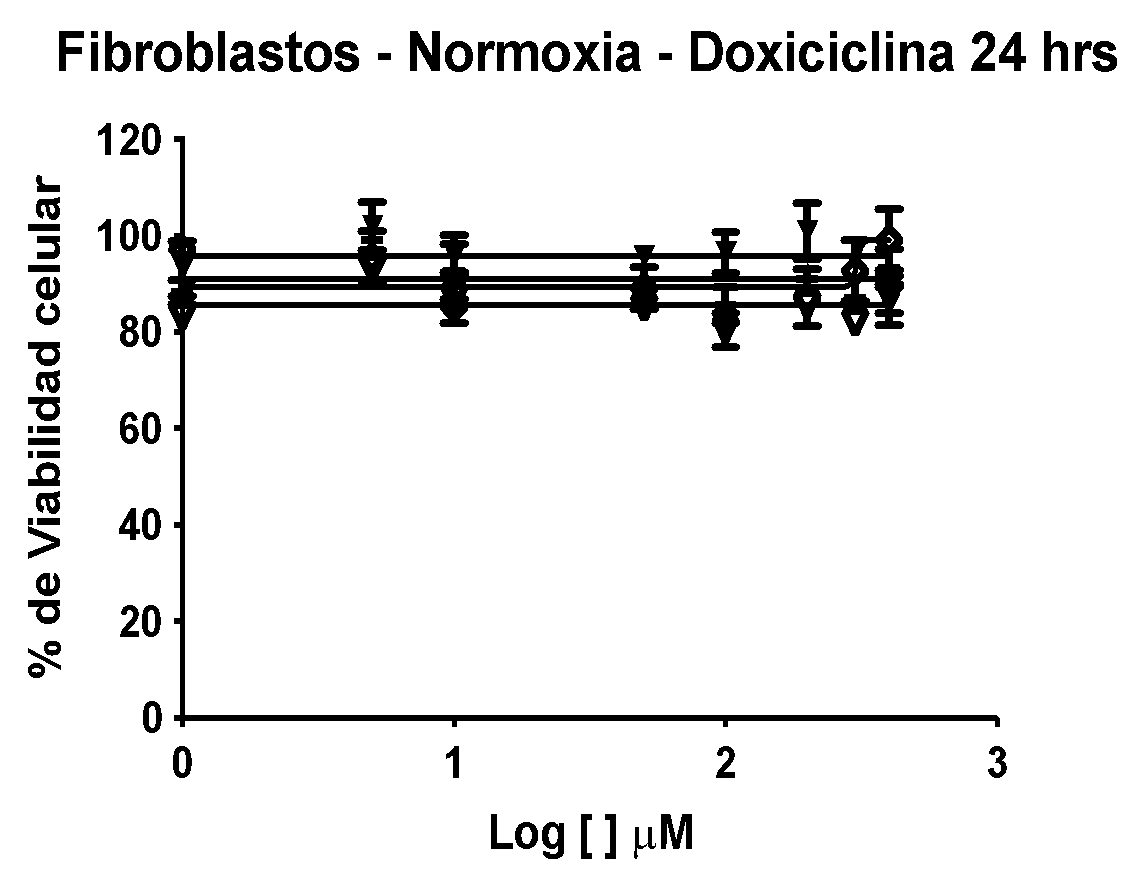**  **A** | **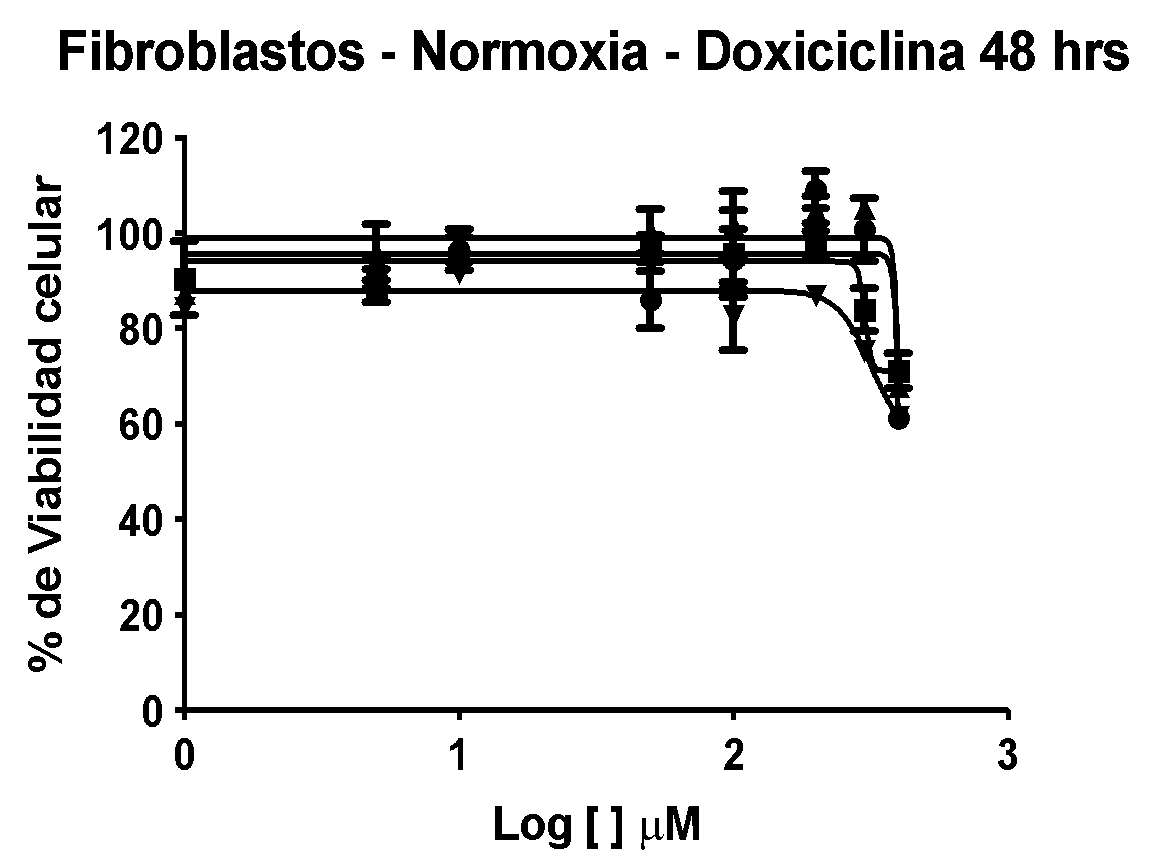**  **B** | **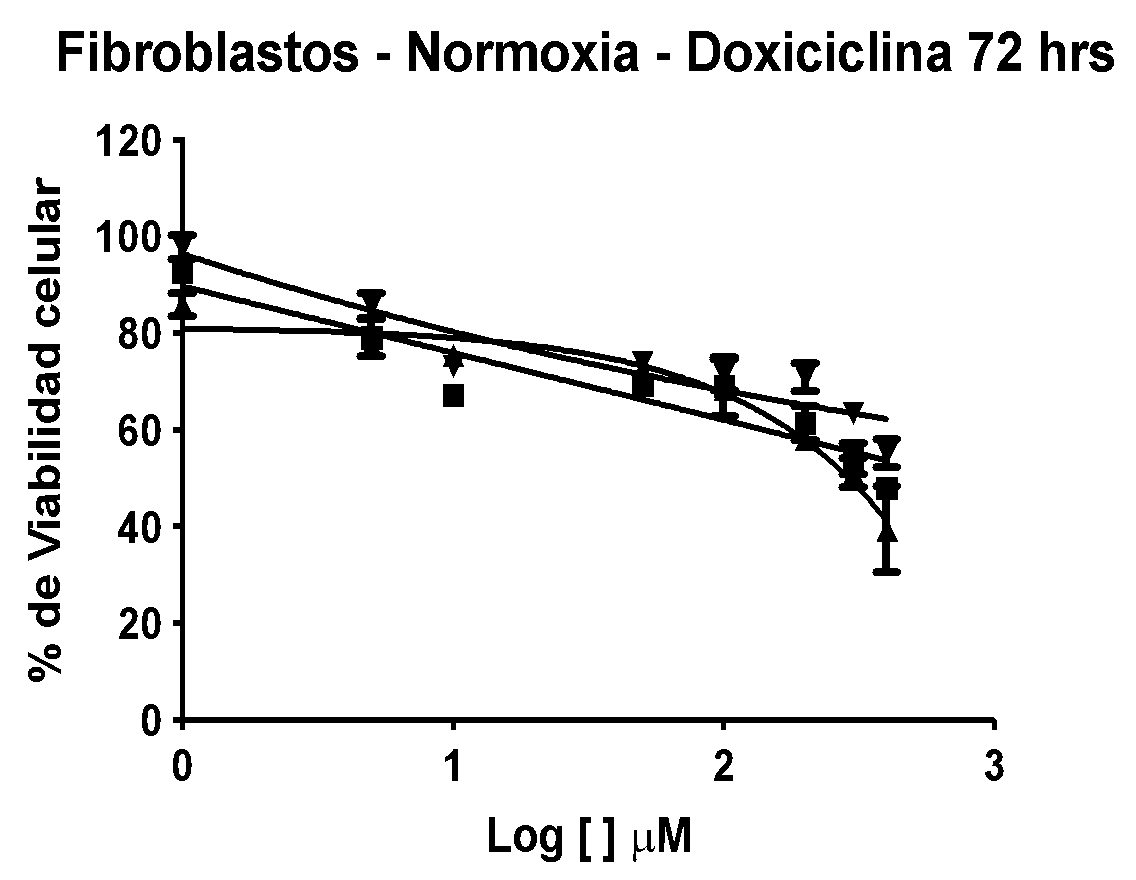**  **C** |
| **IC_50_** | >300 *μM* | >300 *μM* | >300 *μM* |

***Figure. S9.*** Cytotoxic effect of Doxycycline on monolayer cultures of lung fibroblast cell lines under normoxic conditions. Graphical representation of the effect of the compound Doxycycline on cell viability as a function of the logarithm of the concentration. The cytotoxic effect is observed at 24 h (A), 48 h (B) and 72 h (C). Data obtained by averaging at least 3 independent experiments. The averages of the IC_50_ ± SD are shown.

**d. PA-TPP^+^ C10**

**Cell line NCI–H727**

|  | **24 h** | **48 h** | **72 h** |
| --- | --- | --- | --- |
| **Normoxic** | **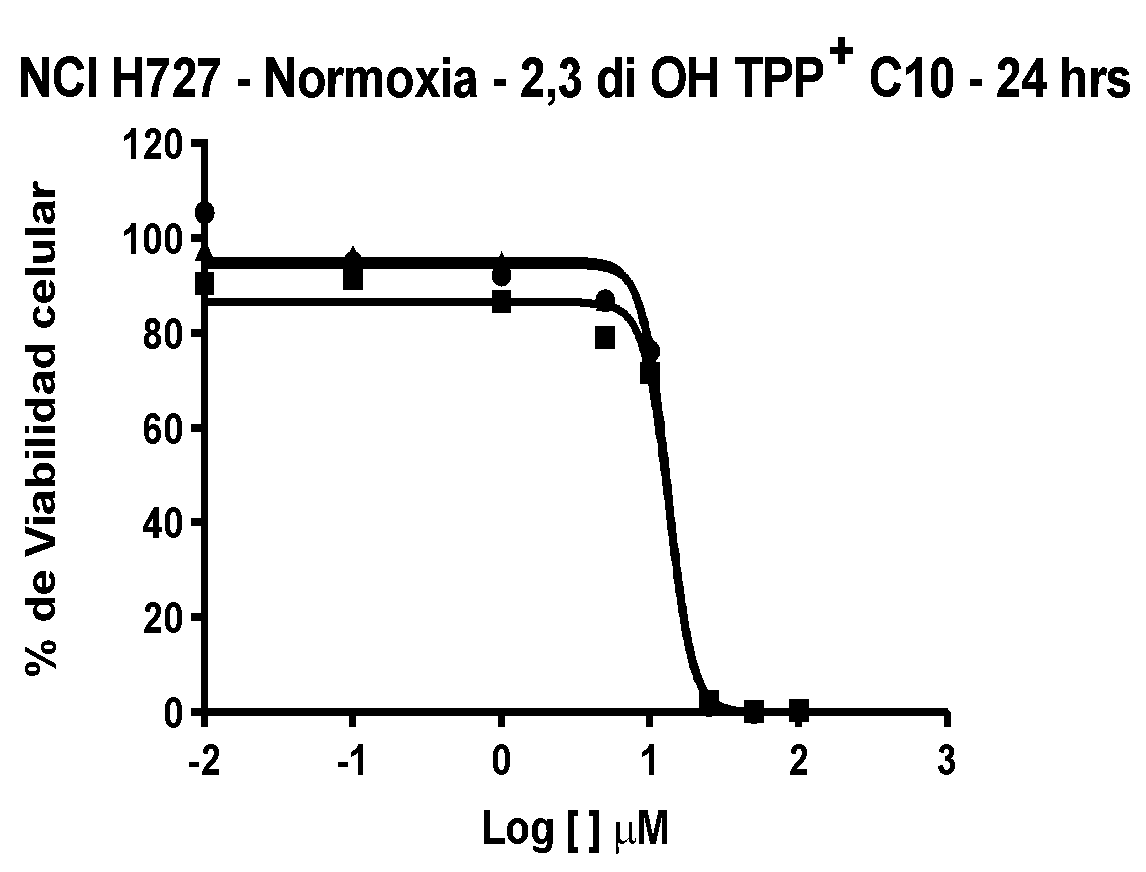**  **A** | **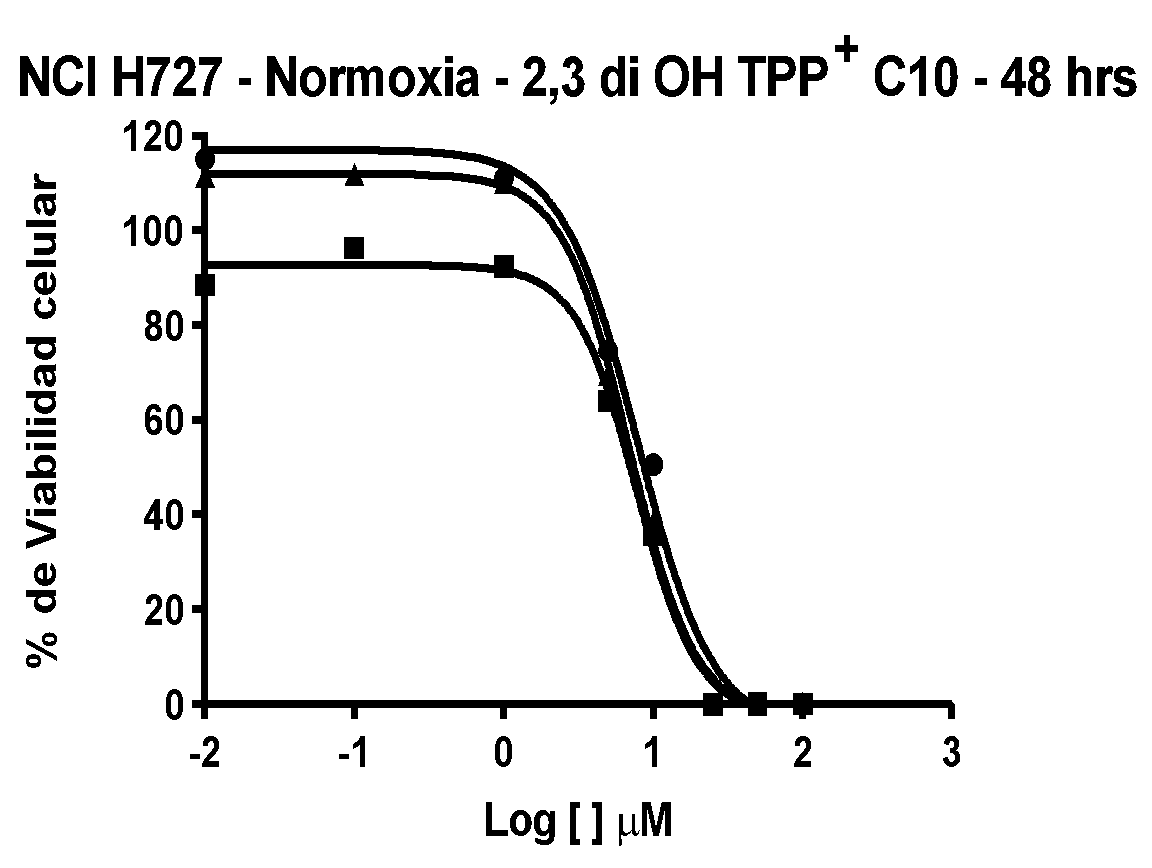**  **B** | **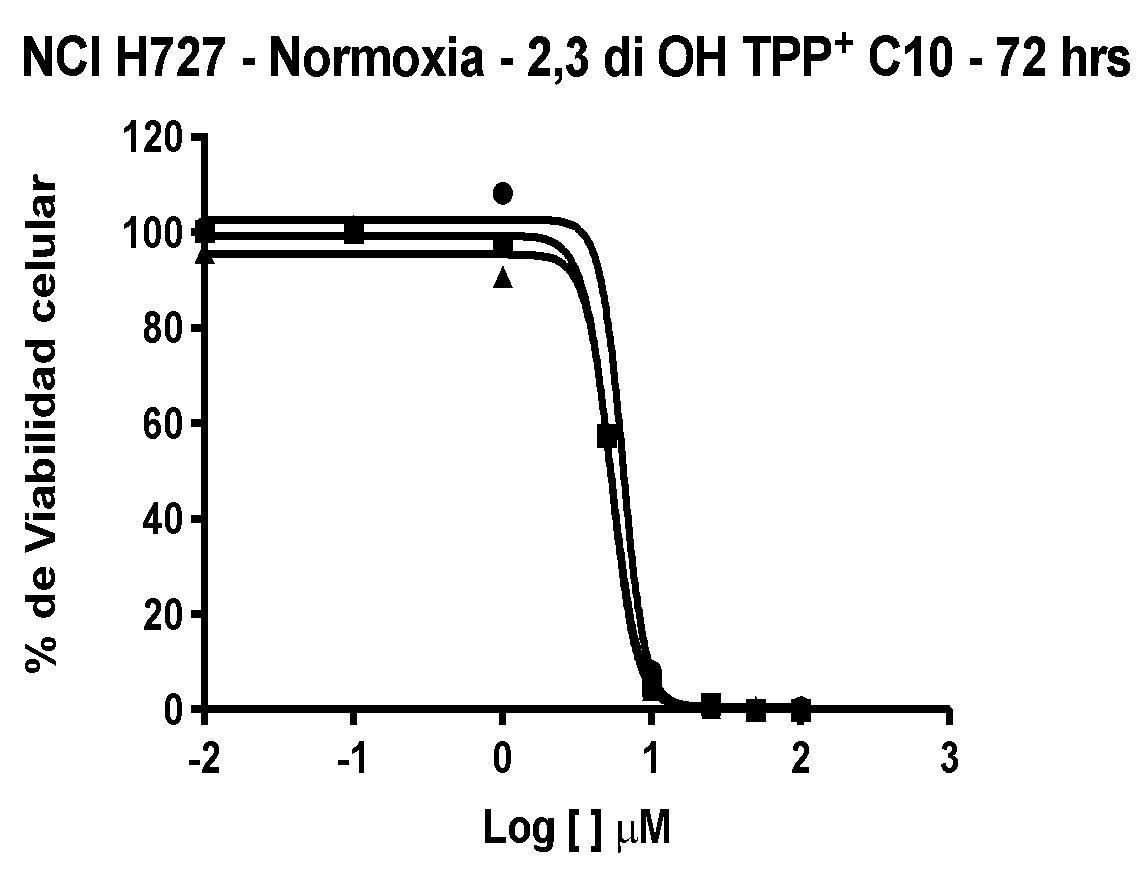**  **C** |
| **IC_50_** | 13,27 ± 0,23 *μM* | 7,54 ± 0,54 *μM* | 5,73 ± 0,62 *μM* |
| **Hipoxia** | **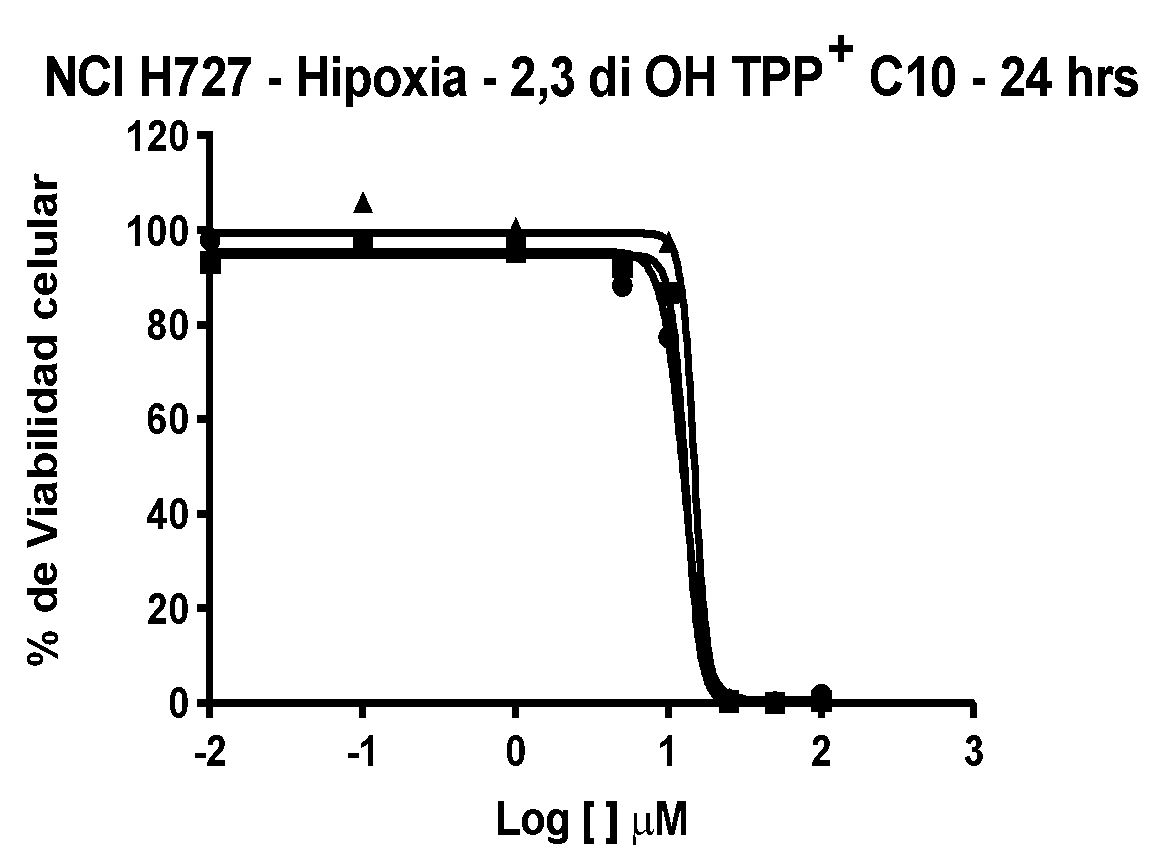**  **D** | **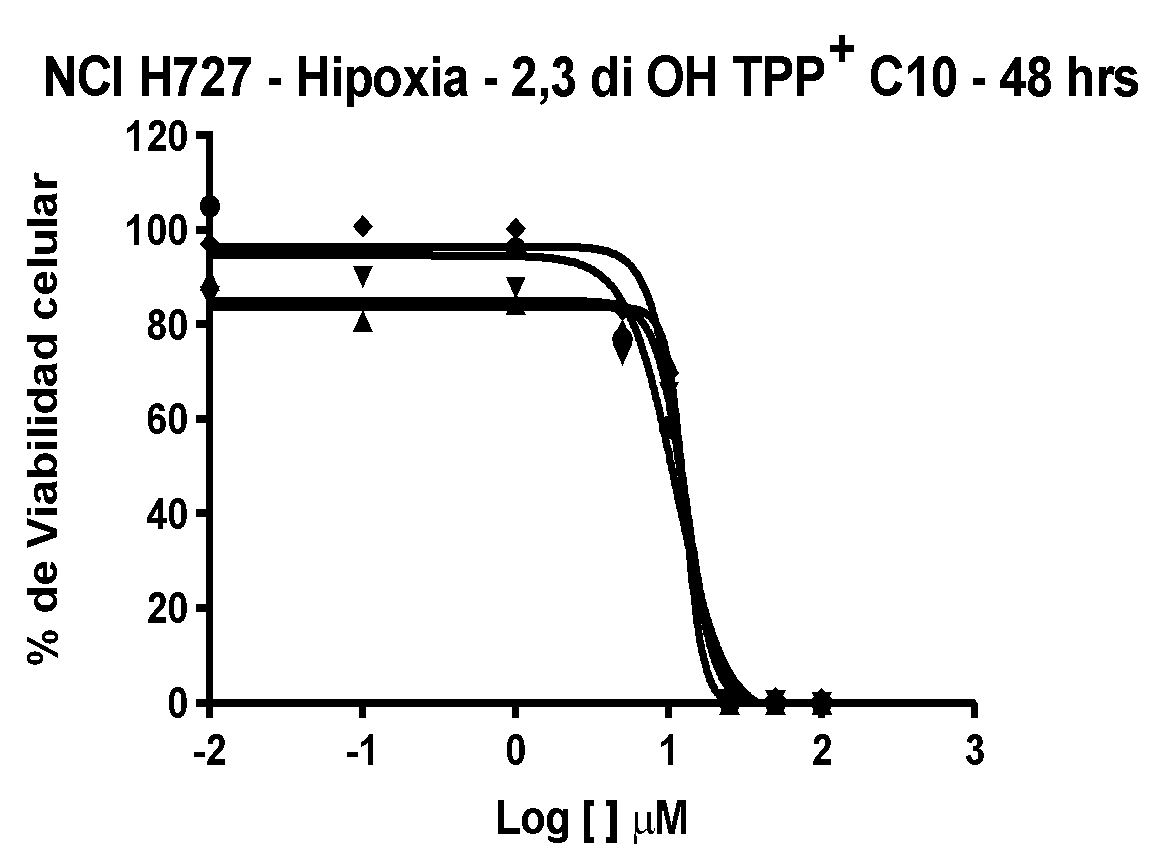**  **E** | **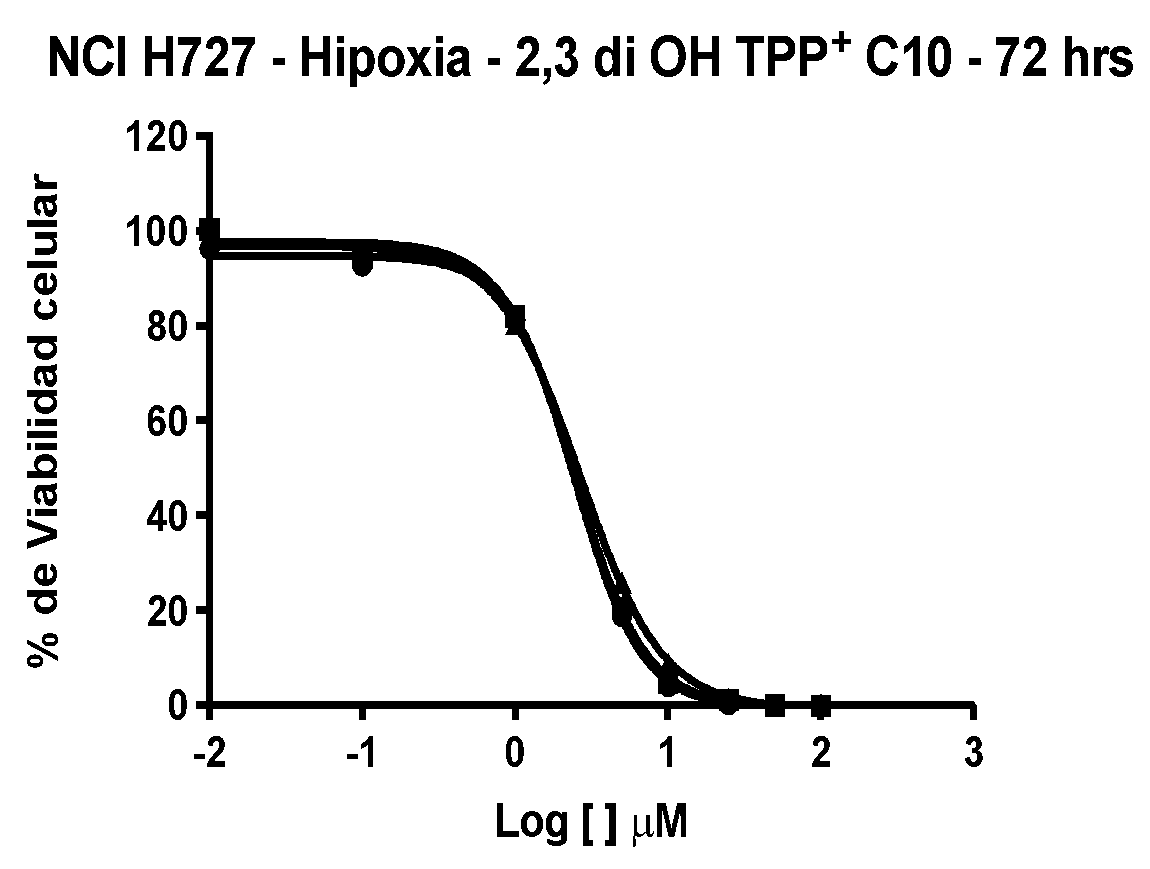**  **F** |
| **IC_50_** | 13,52 ± 1,24 *μM* | 12,34 ± 0,79 *μM* | 2,55 ± 0,13 *μM* |

**Figure S10.** Cytotoxic effect of PA-TPP^+^C_10_ on NCI H727 cell line monolayer cultures under normoxic and hypoxic conditions. Graphical representation of the effect of the compound PA-TPP^+^C_10_ on cell viability as a function of the logarithm of the concentration. The cytotoxic effect is observed at 24 hr (A and D), 48 hr (B and E) and 72 hr (C and F). Data obtained by 3 independent experiments. The averages of the IC_50_ ± SD are shown.

**Cell line NCI–H1299**

|  | **24 h** | **48 h** | **72 h** |
| --- | --- | --- | --- |
| **Normoxic** | **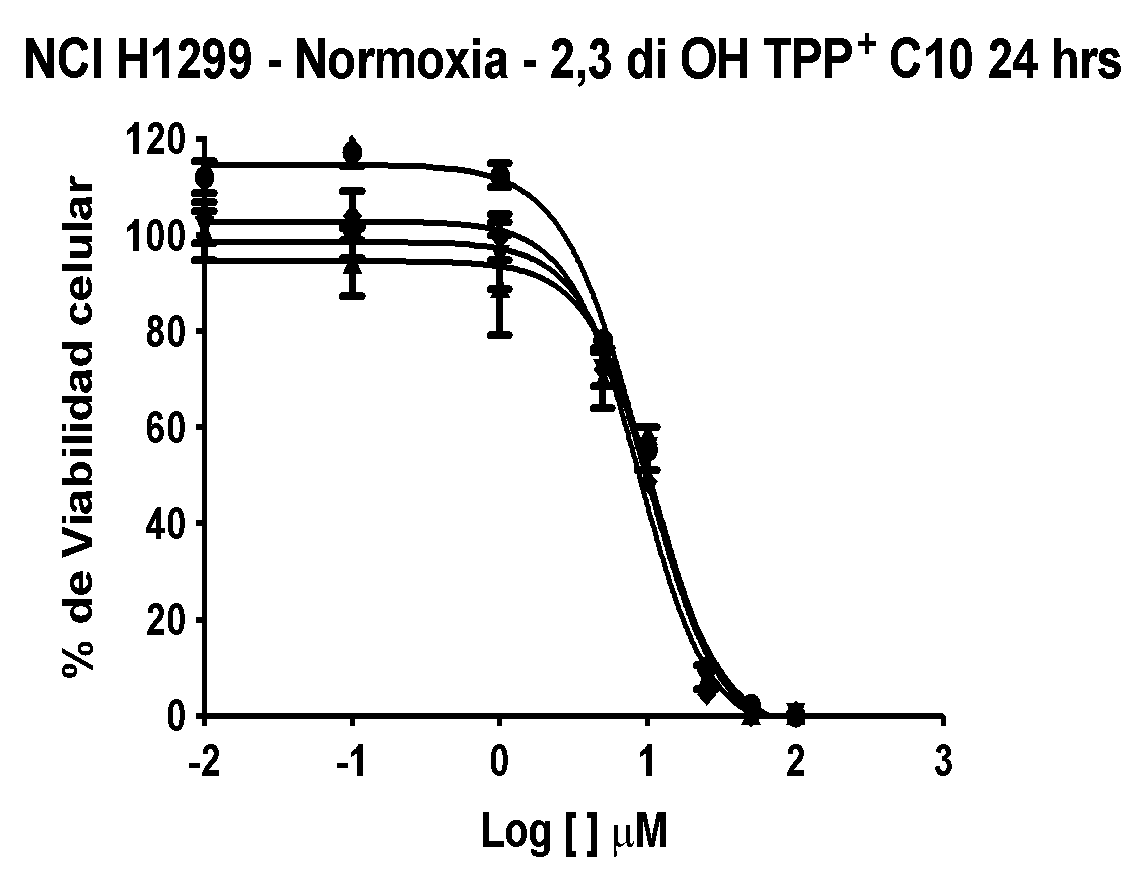**  **A** | **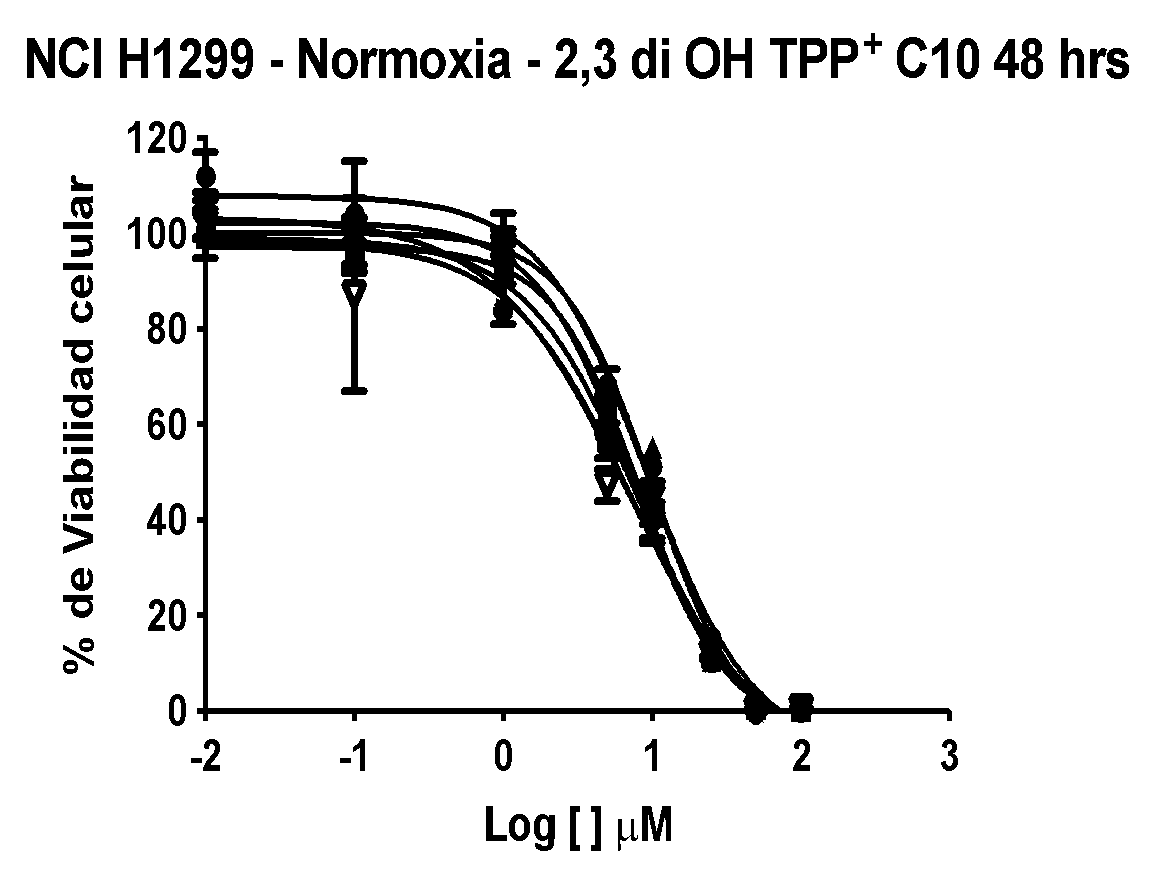**  **B** | **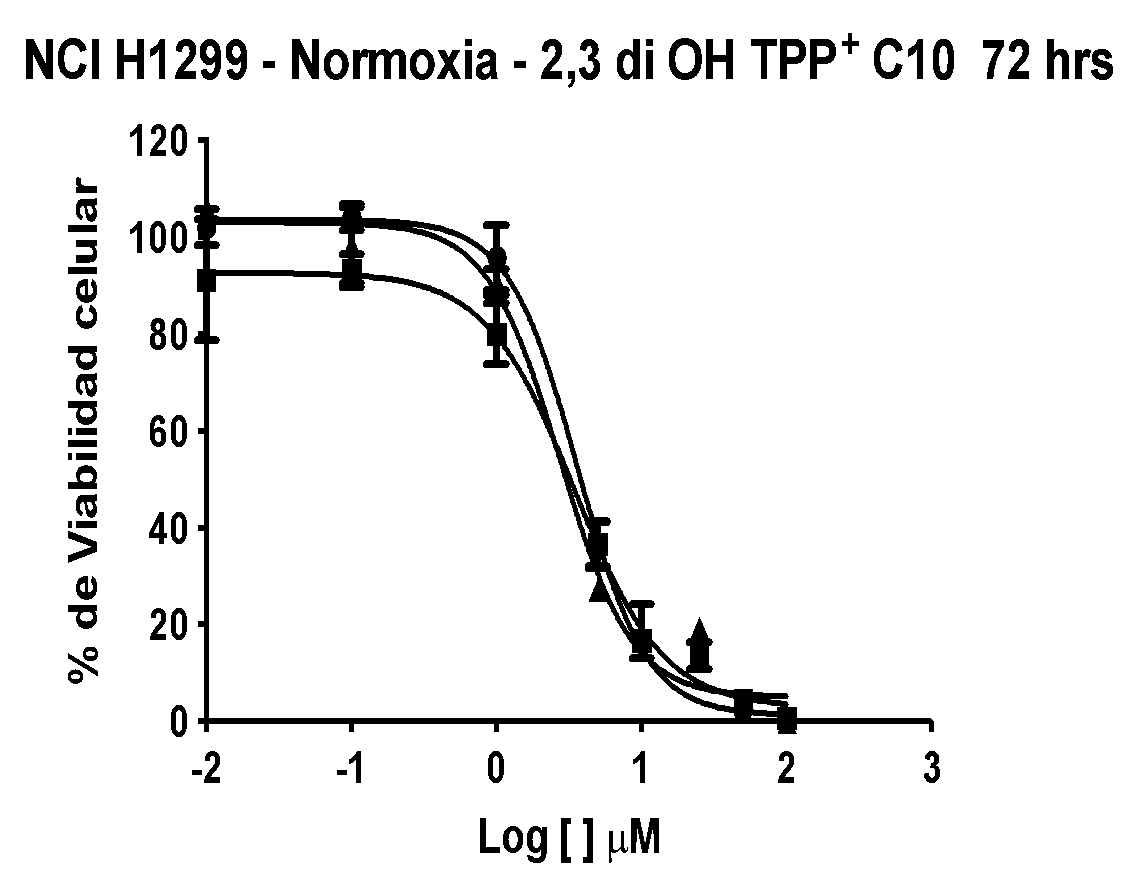**  **C** |
| **IC_50_** | 9,93 ± 1,2 *μM* | 8,19 ± 1,12 *μM* | 3,29 ± 0,47 *μM* |
| **Hipoxia** | **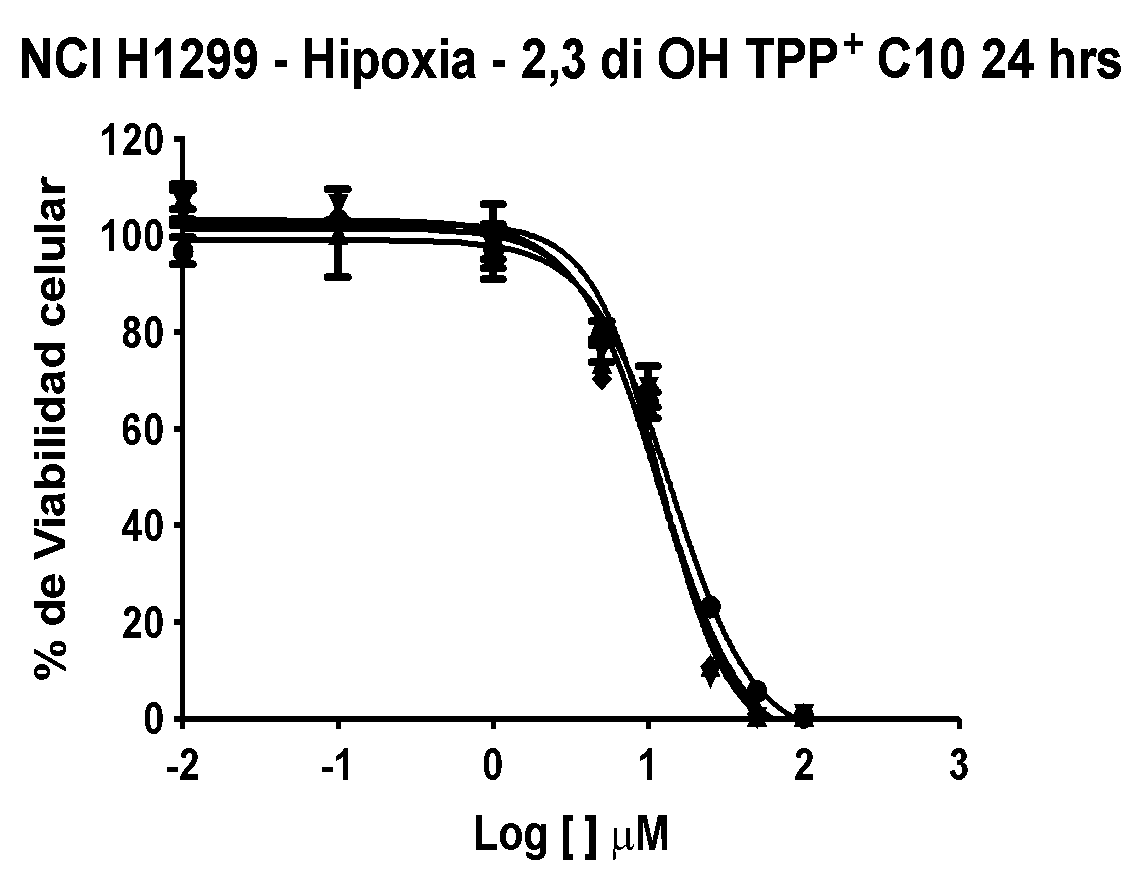**  **D** | **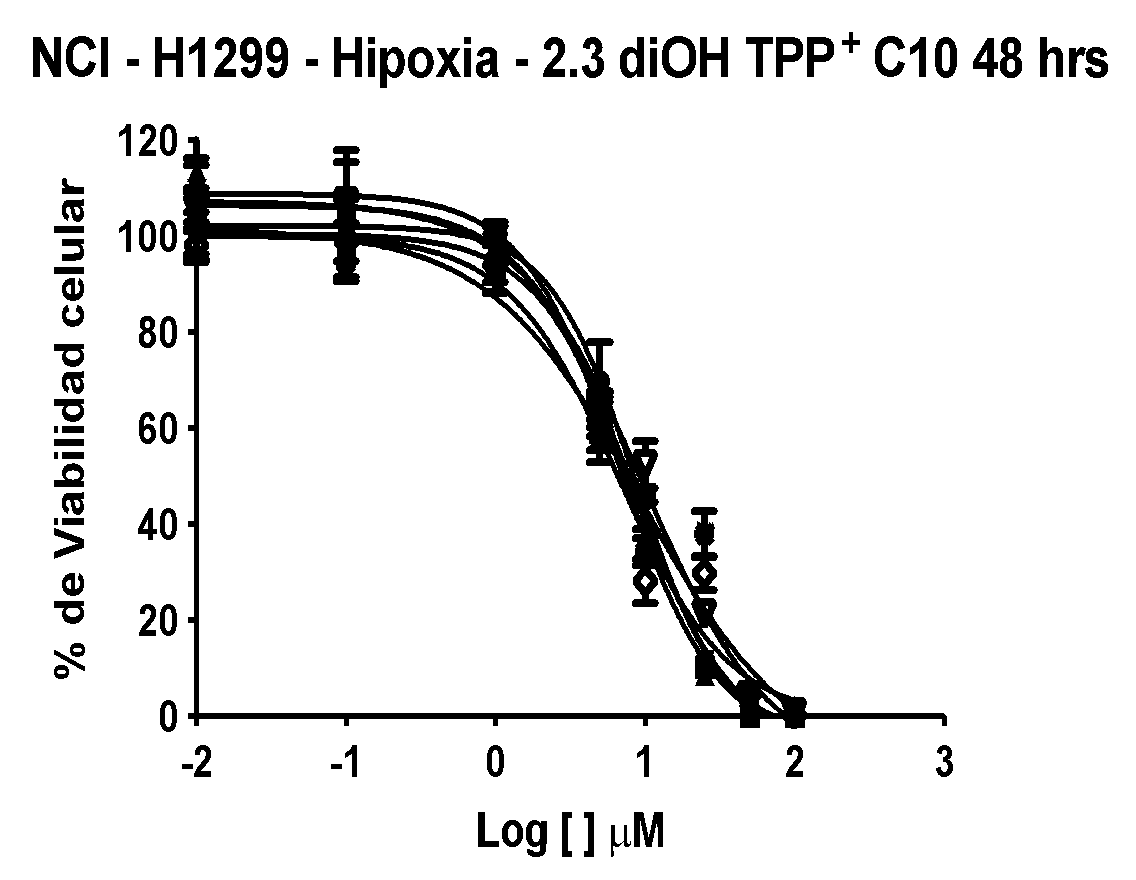**  **E** | **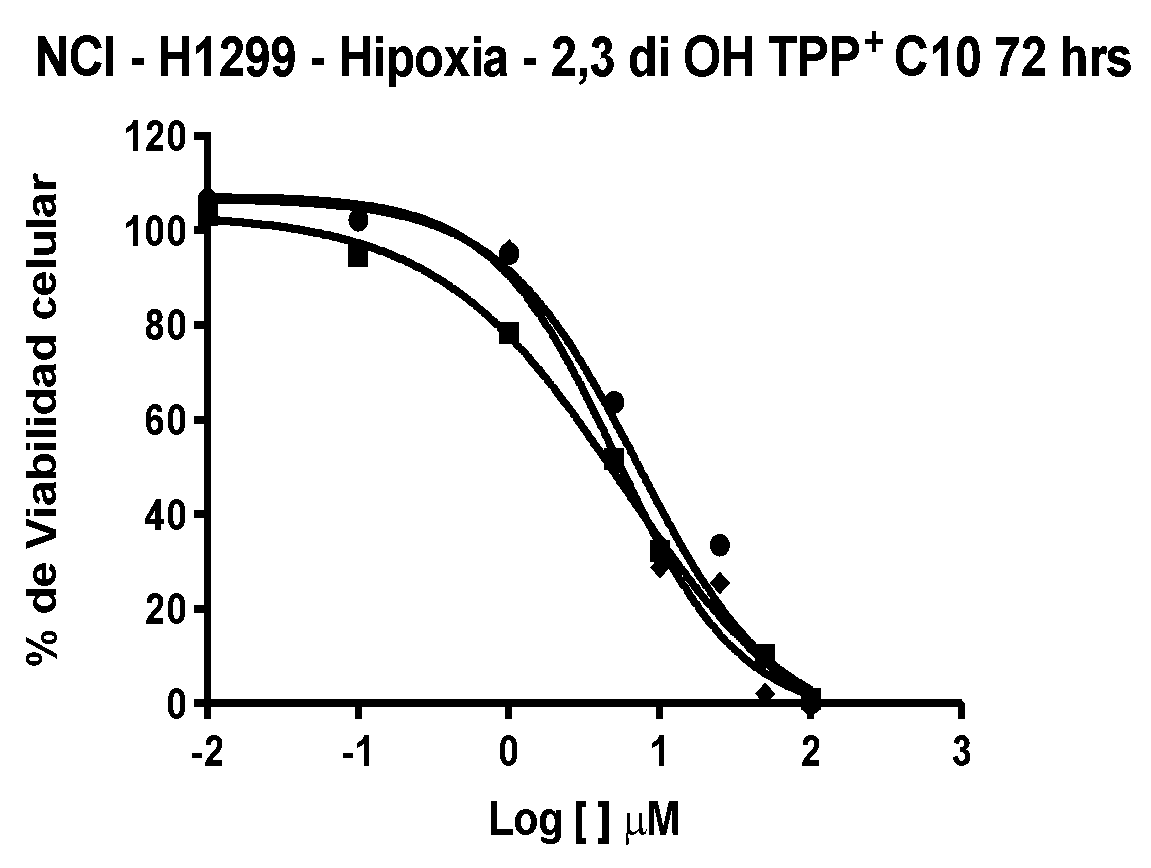**  **F** |
| **IC_50_** | 12,53 ± 1,17 *μM* | 8,27 ± 1,21 *μM* | 6,06 ± 1,03 *μM* |

**Figure S11.** Cytotoxic effect of PA-TPP^+^C_10_ on NCI H1299 cell line monolayer cultures under normoxic and hypoxic conditions. Graphical representation of the effect of the compound PA-TPP^+^C_10_ on cell viability as a function of the logarithm of the concentration. The cytotoxic effect is observed at 24 h (A and D), 48 h (B and E) and 72 h (C and F). Data obtained by averaging at least 3 independent experiments. The averages of the IC_50_ ± SD are shown.

**Lung fibroblast**

|  | **24 h** | **48 h** | **72 h** |
| --- | --- | --- | --- |
| **Normoxic** | **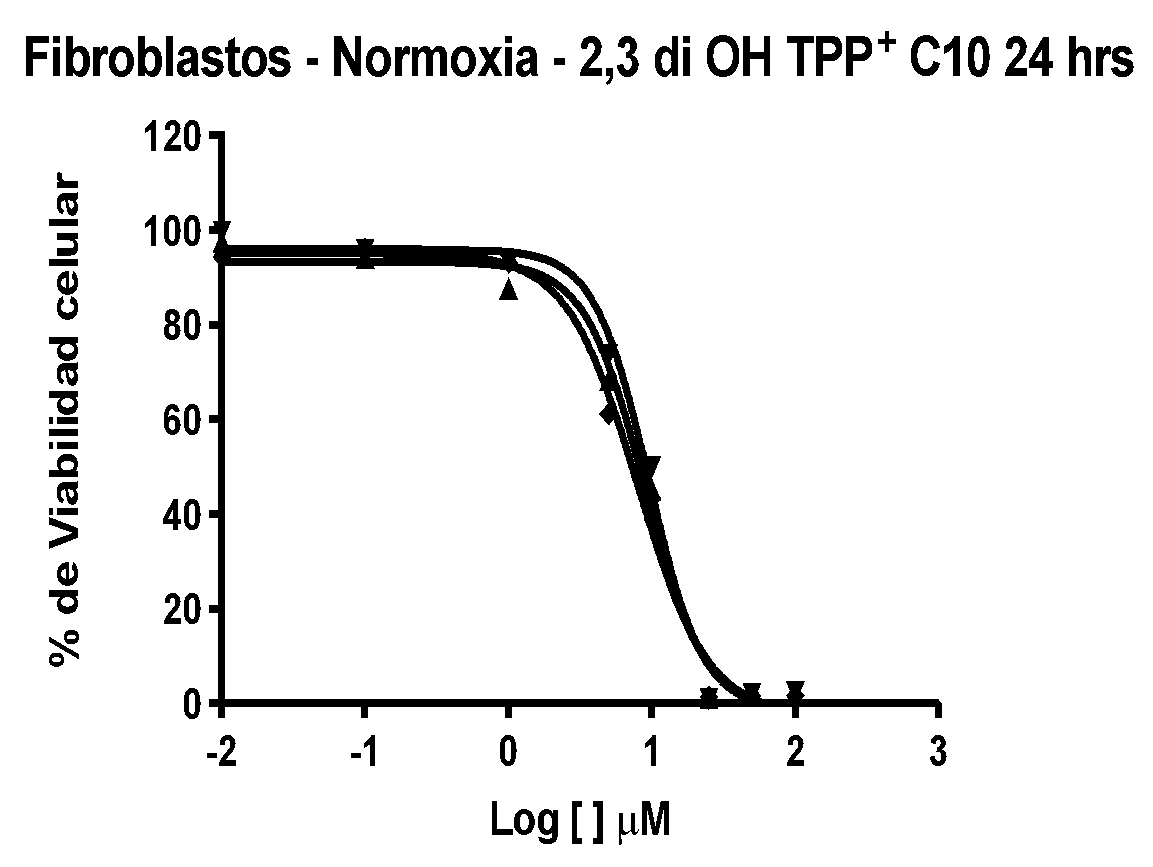**  **A** | **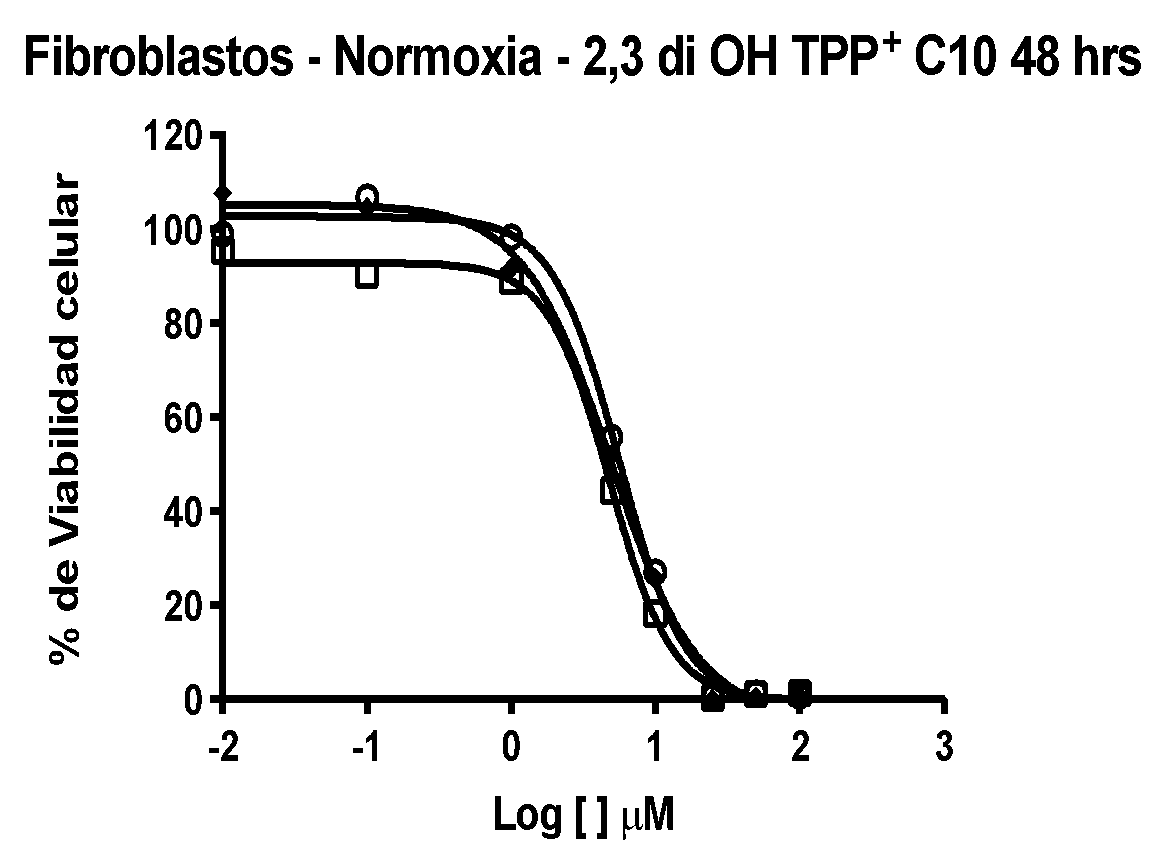**  **B** | **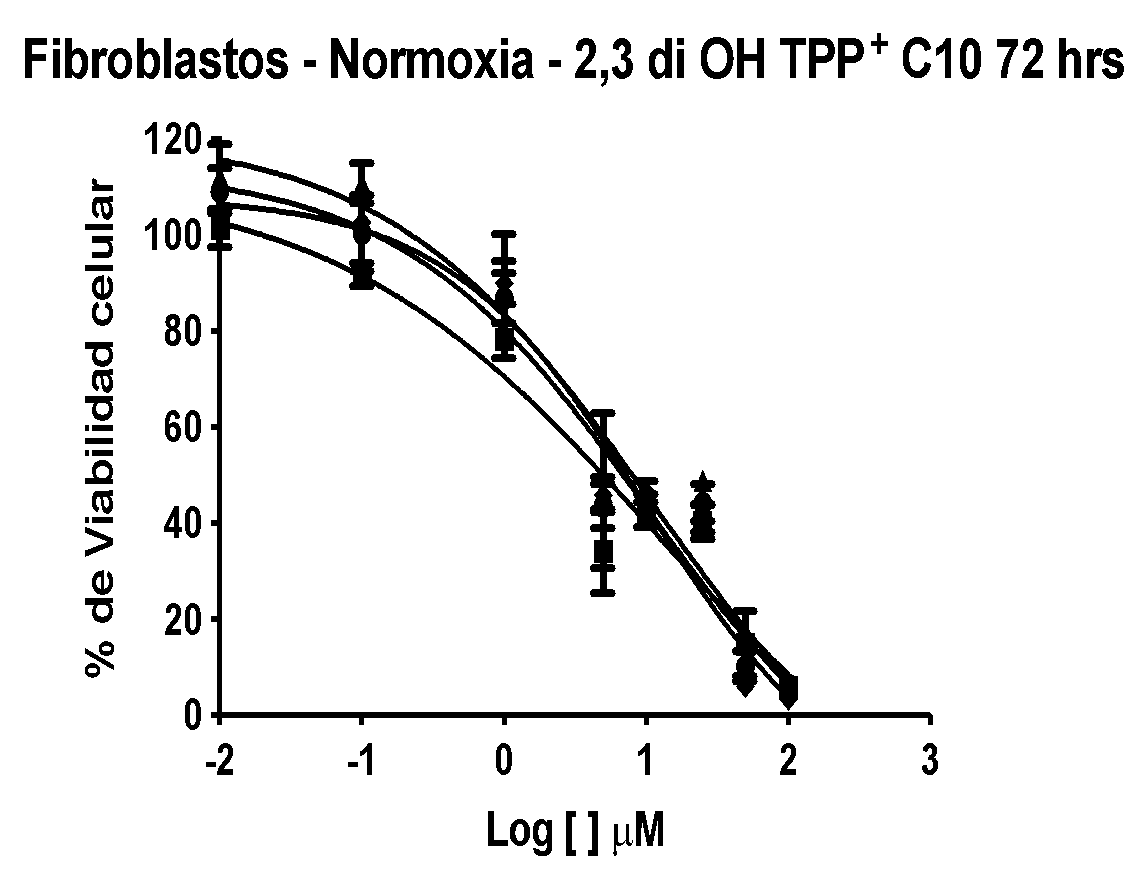**  **C** |
| **IC_50_** | 8.86 ± 0,79 *μM* | 5,13 ± 0,44 *μM* | 10,96 ± 1,16 *μM* |

**Figure S12.** Cytotoxic effect of PA-TPP^+^C_10_ on lung fibroblast cell line monolayer cultures under normoxic conditions. Graphical representation of the effect of the compound PA-TPP^+^C_10_ on cell viability as a function of the logarithm of the concentration. The cytotoxic effect is observed at 24 h (A), 48 h (B) and 72 h (C). Data obtained by averaging at least 3 independent experiments. The averages of the IC_50_ ± SD are shown.

**e. SA-TPP^+^ C10**

**Cell line NCI–H727**

|  | **24 h** | **48 h** | **72 h** |
| --- | --- | --- | --- |
| **Normoxic** | **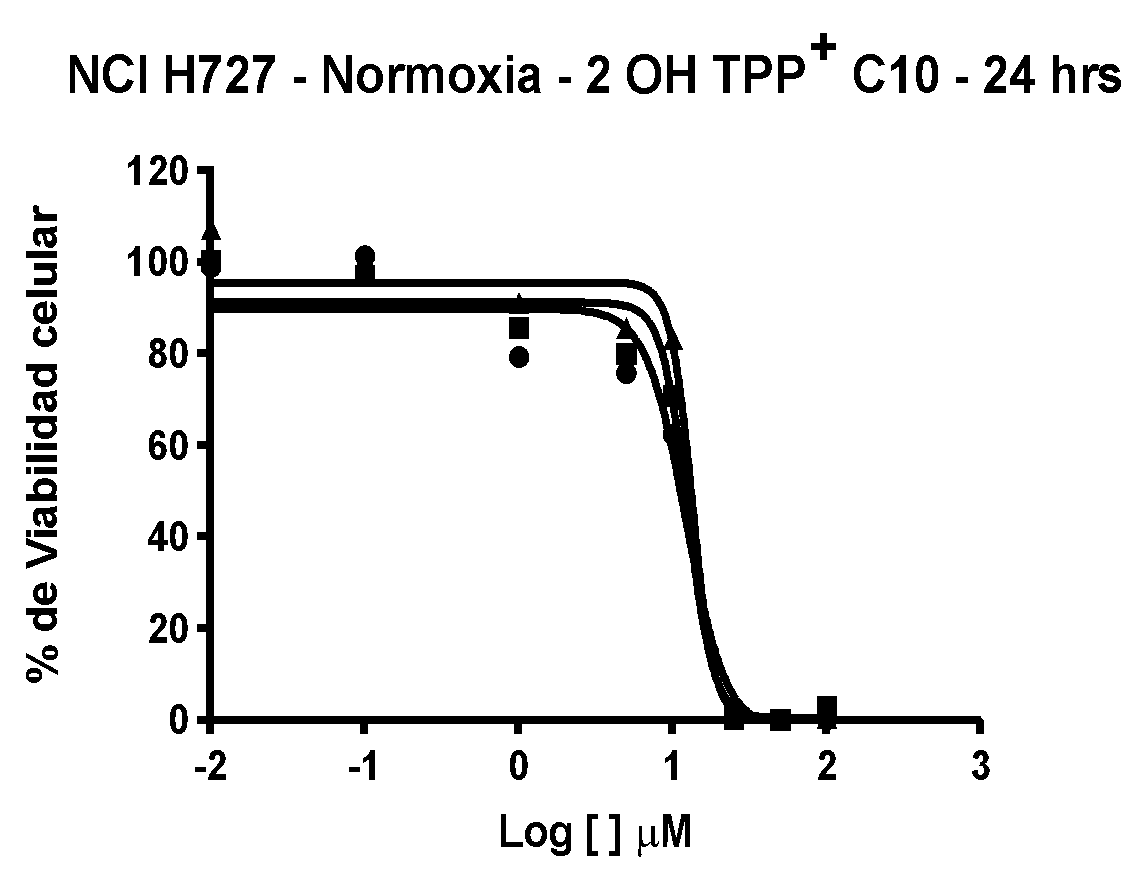**  **A** | **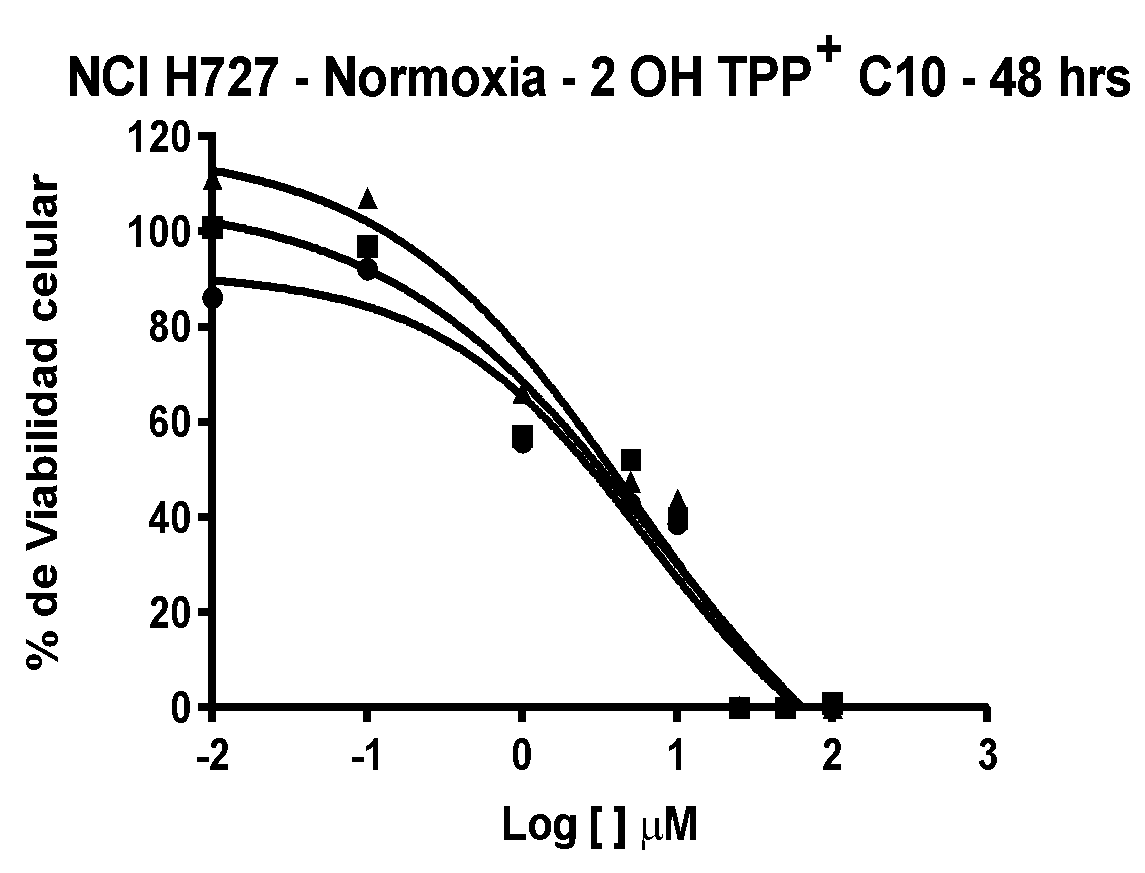**  **B** | **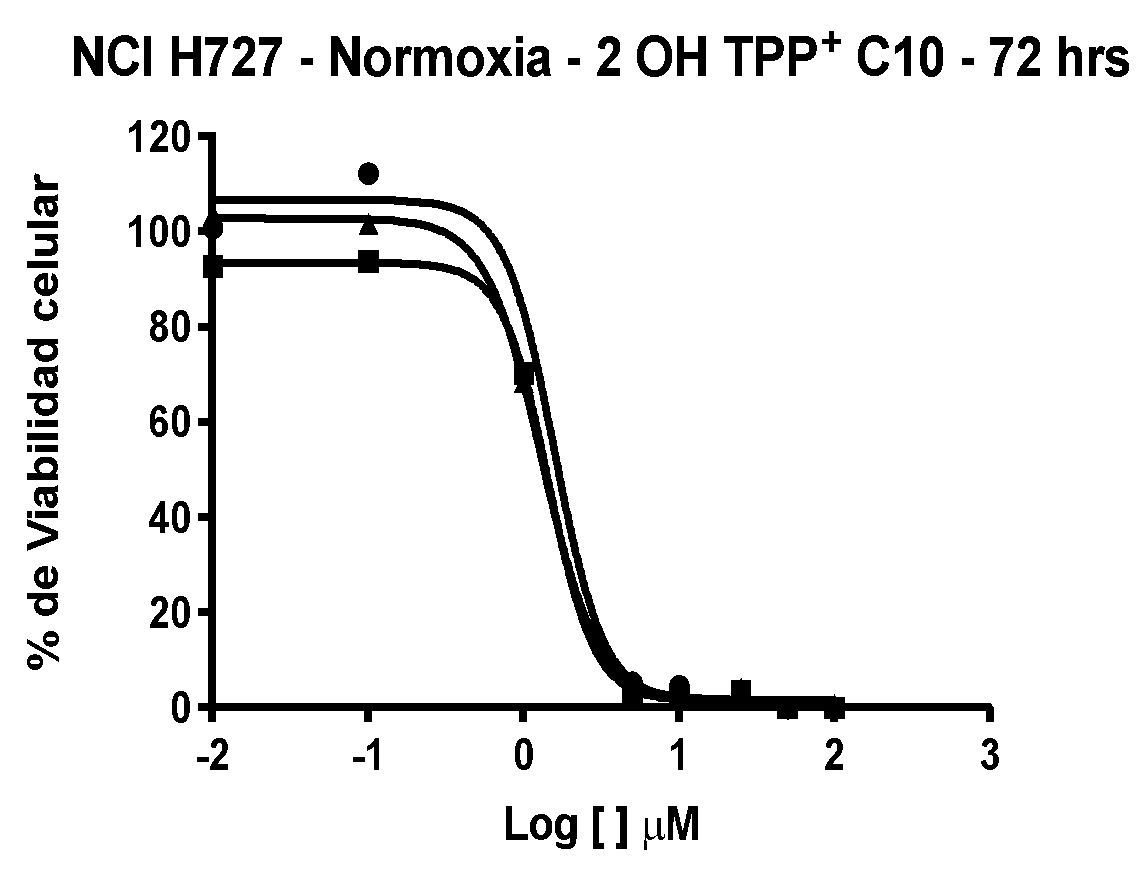**  **C** |
| **IC_50_** | 12,76 ± 0,63 *μM* | 6,46 ± 1,14 *μM* | 1,45 ± 0,14 *μM* |
| **Hipoxia** | **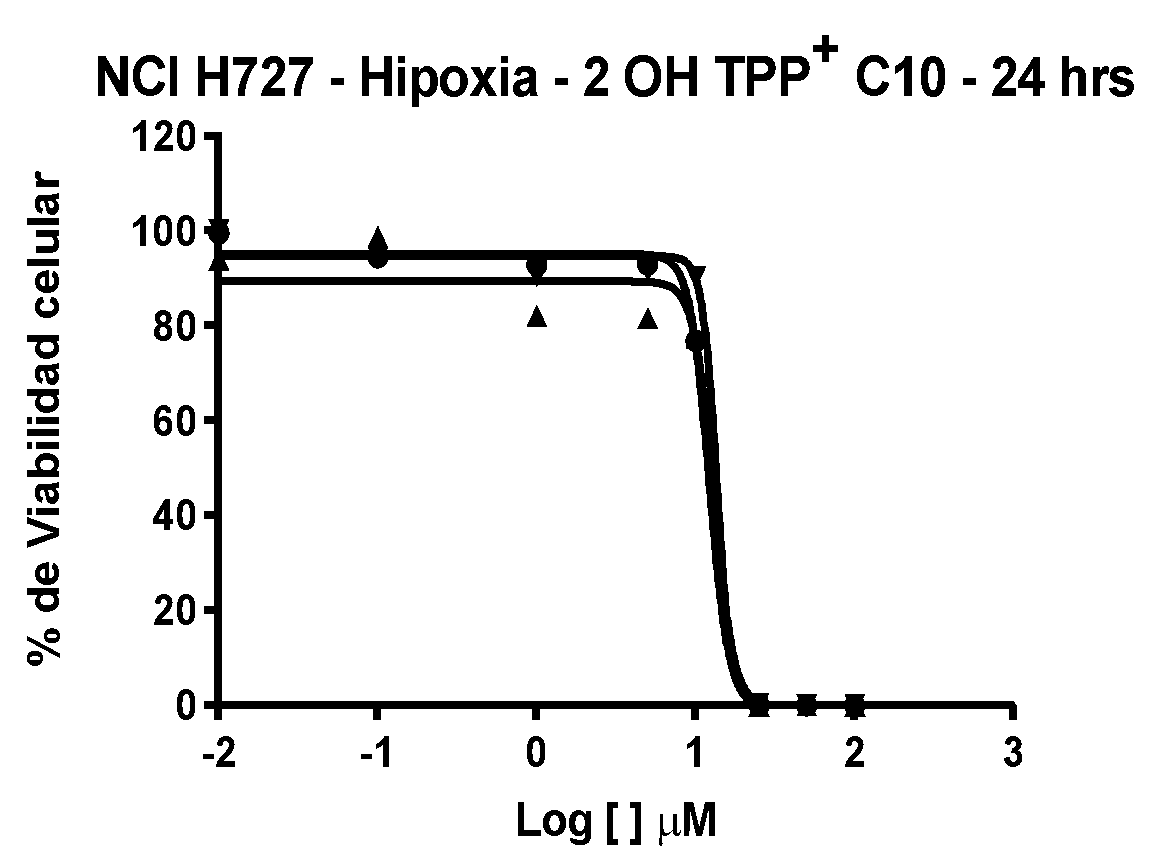**  **D** | **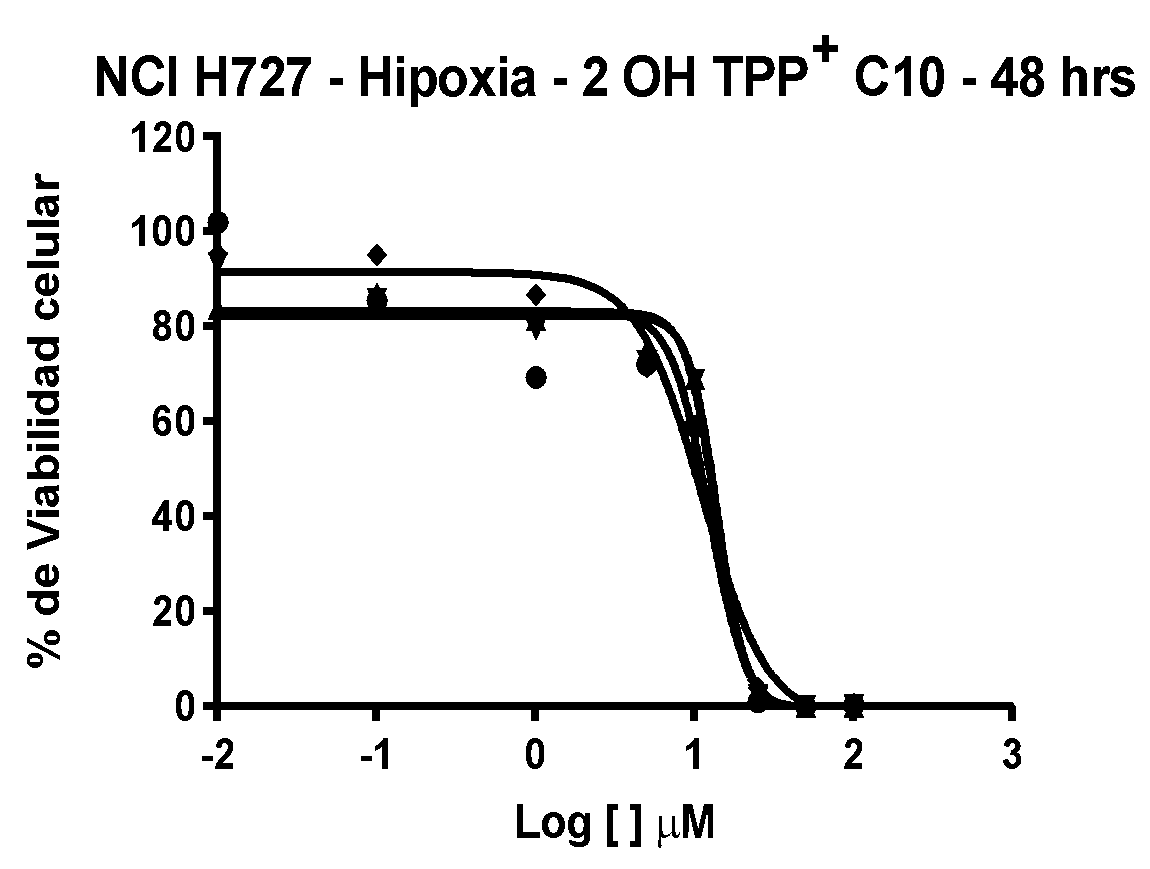**  **E** | **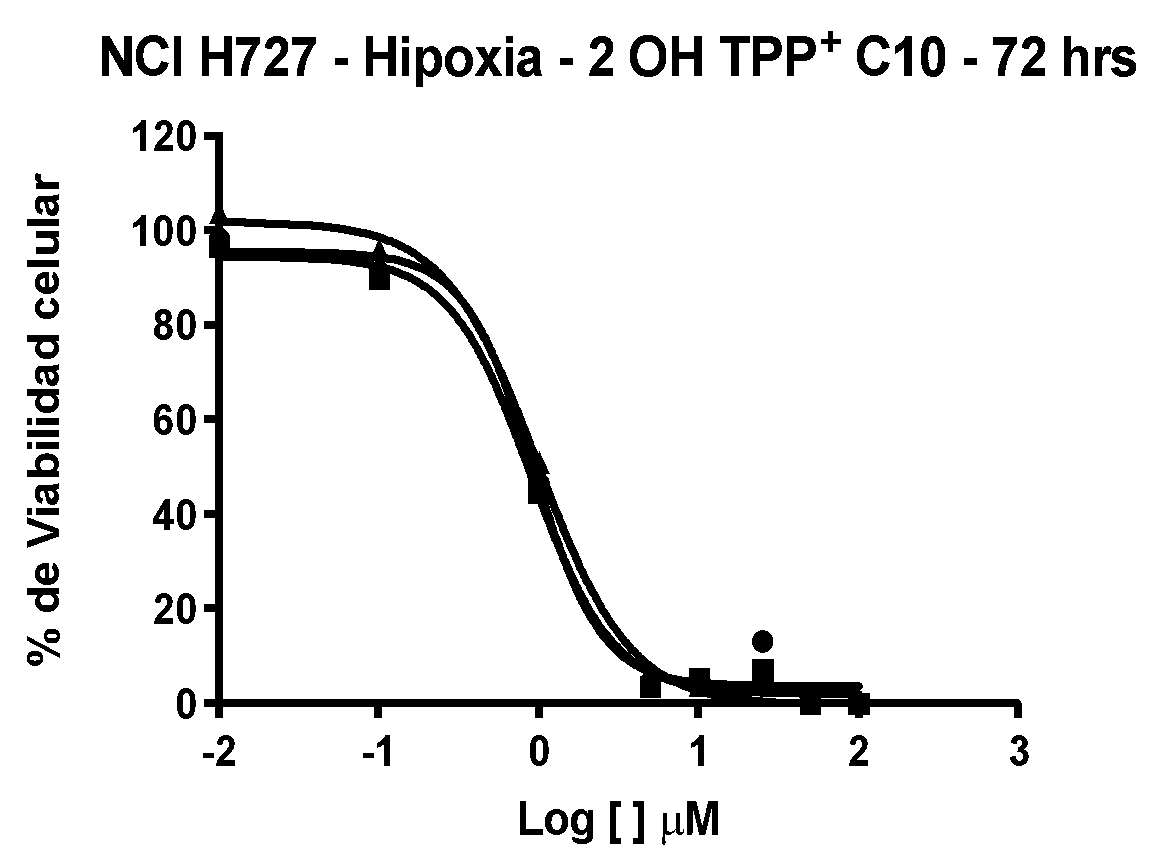**  **F** |
| **IC_50_** | 13,0 ± 0,71 *μM* | 12,8 ± 1,2 *μM* | 0,94 ± 0,05 *μM* |

**Figure S13.** Cytotoxic effect of SA-TPP^+^C_10_ on NCI H727 cell line monolayer cultures under normoxic and hypoxic conditions. Graphical representation of the effect of the compound SA-TPP+C10 on cell viability as a function of the logarithm of the concentration. The cytotoxic effect is observed at 24 h (A and D), 48 h (B and E) and 72 h (C and F). Data obtained by averaging at least 3 independent experiments. The averages of the IC_50_ ± SD are shown.

**Cel line NCI – H1299**

|  | **24 h** | **48 h** | **72 h** |
| --- | --- | --- | --- |
| **Normoxic** | **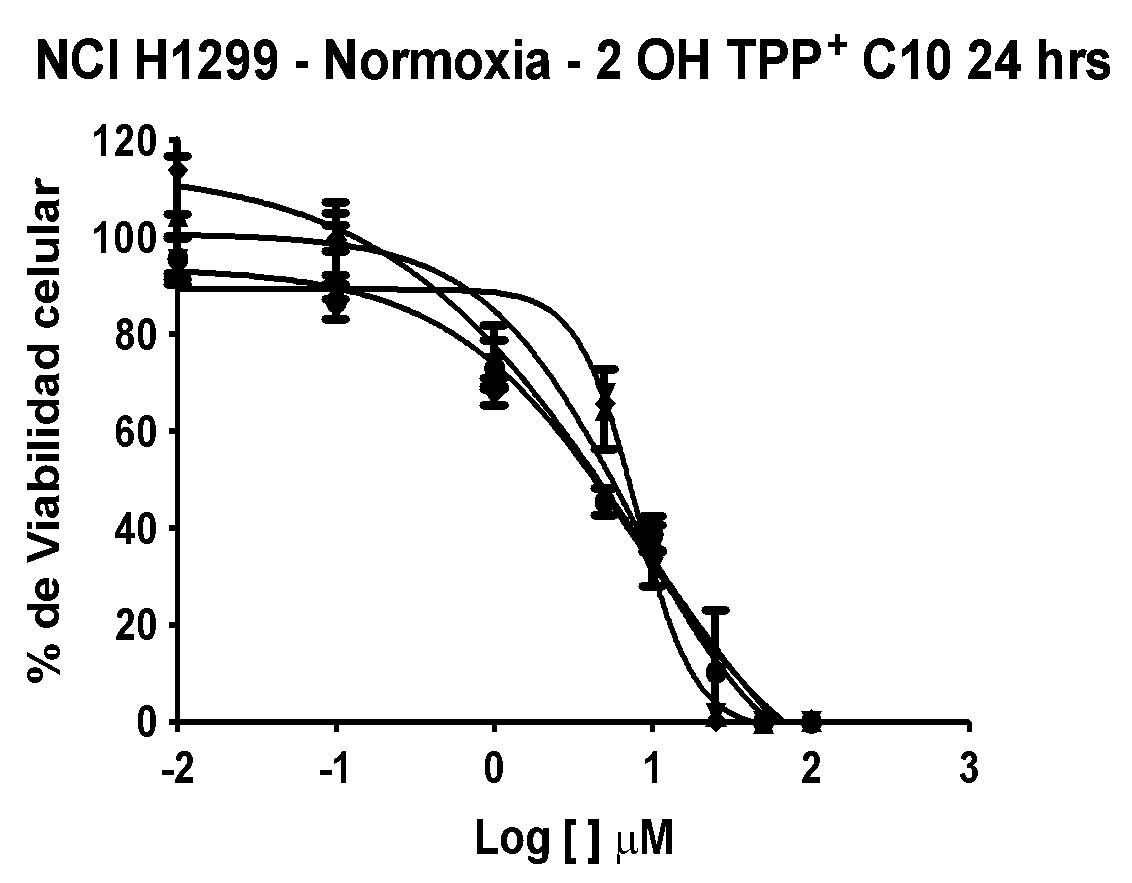**  **A** | **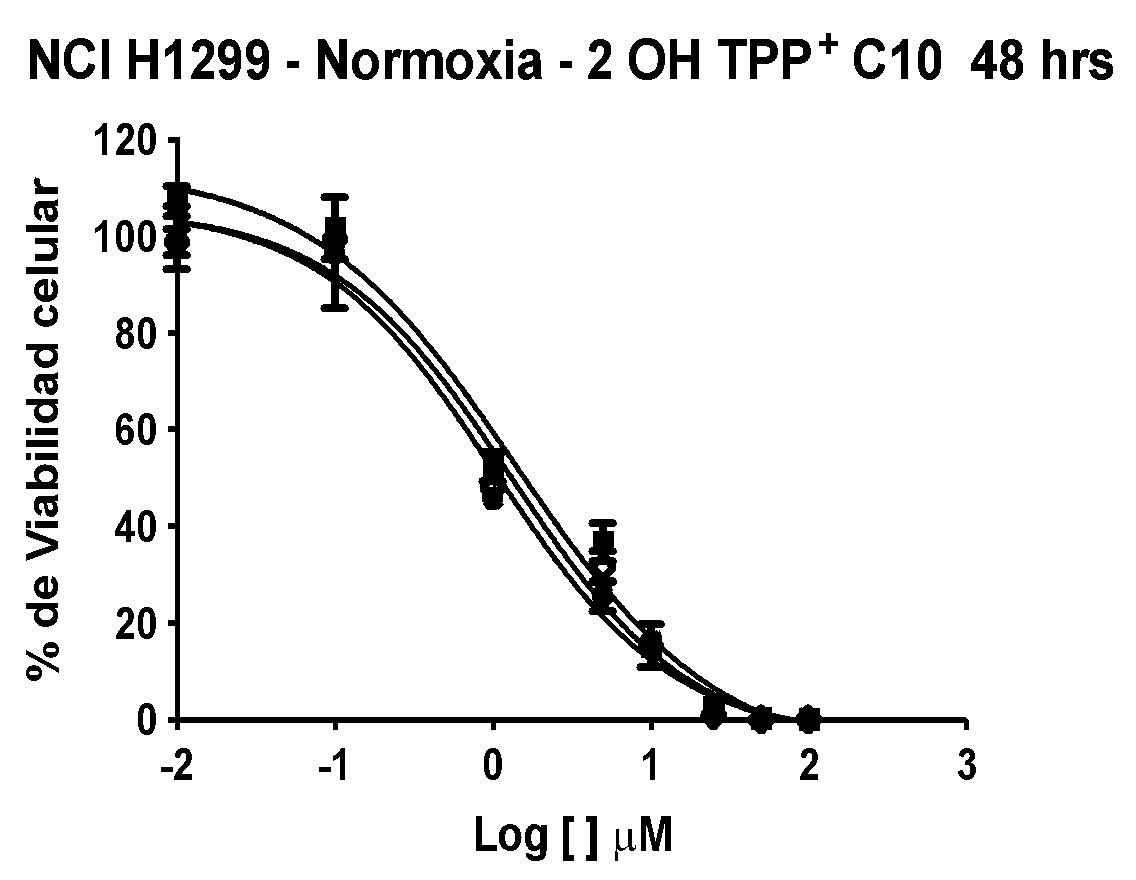**  **B** | **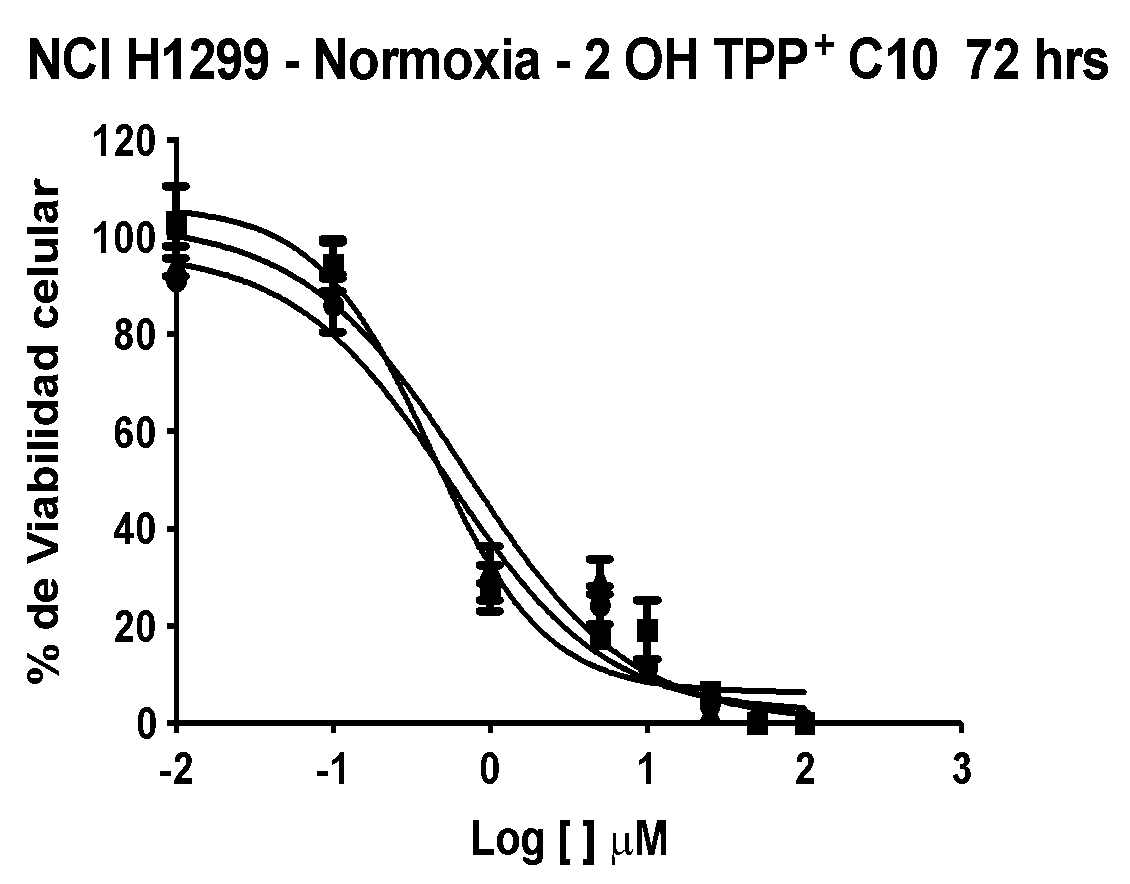**  **C** |
| **IC_50_** | 7,68 ± 0,34 *μM* | 1,25 ± 0,18 *μM* | 0,56 ± 0,14 *μM* |
| **Hipoxia** | **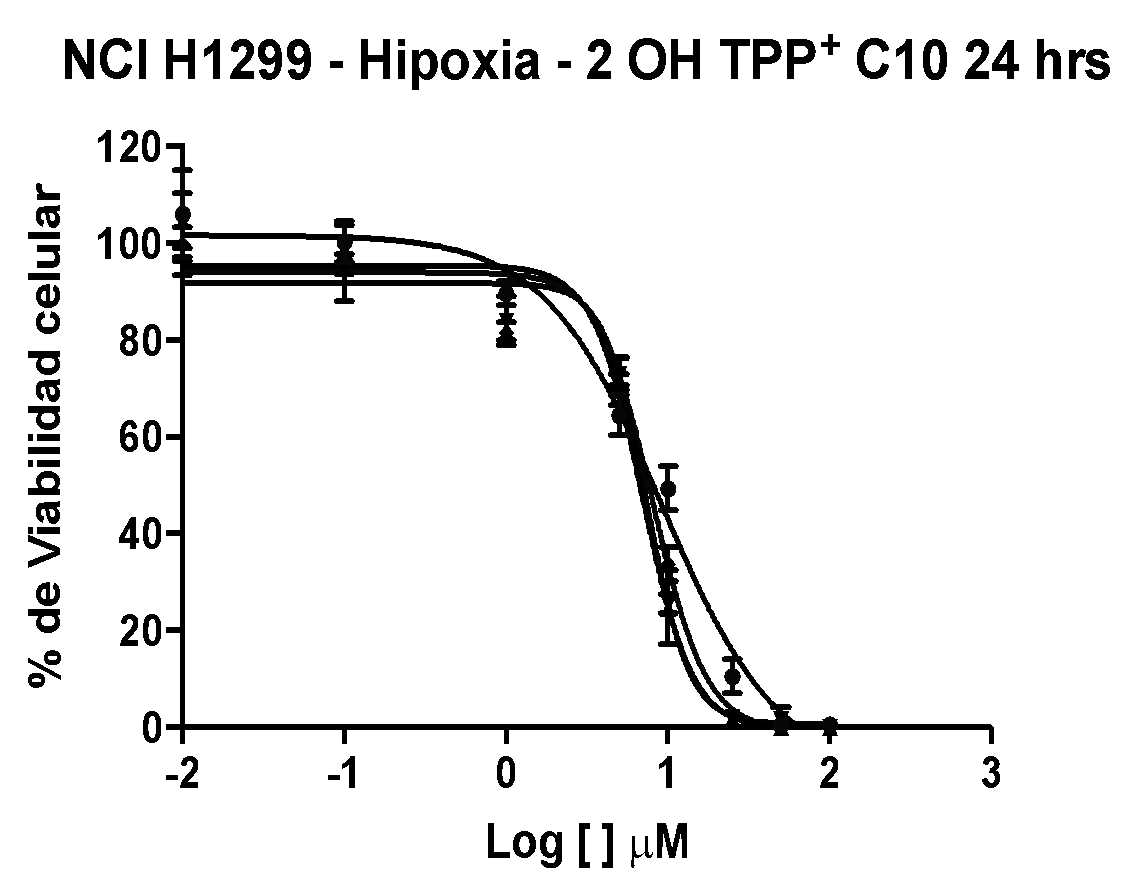**  **D** | **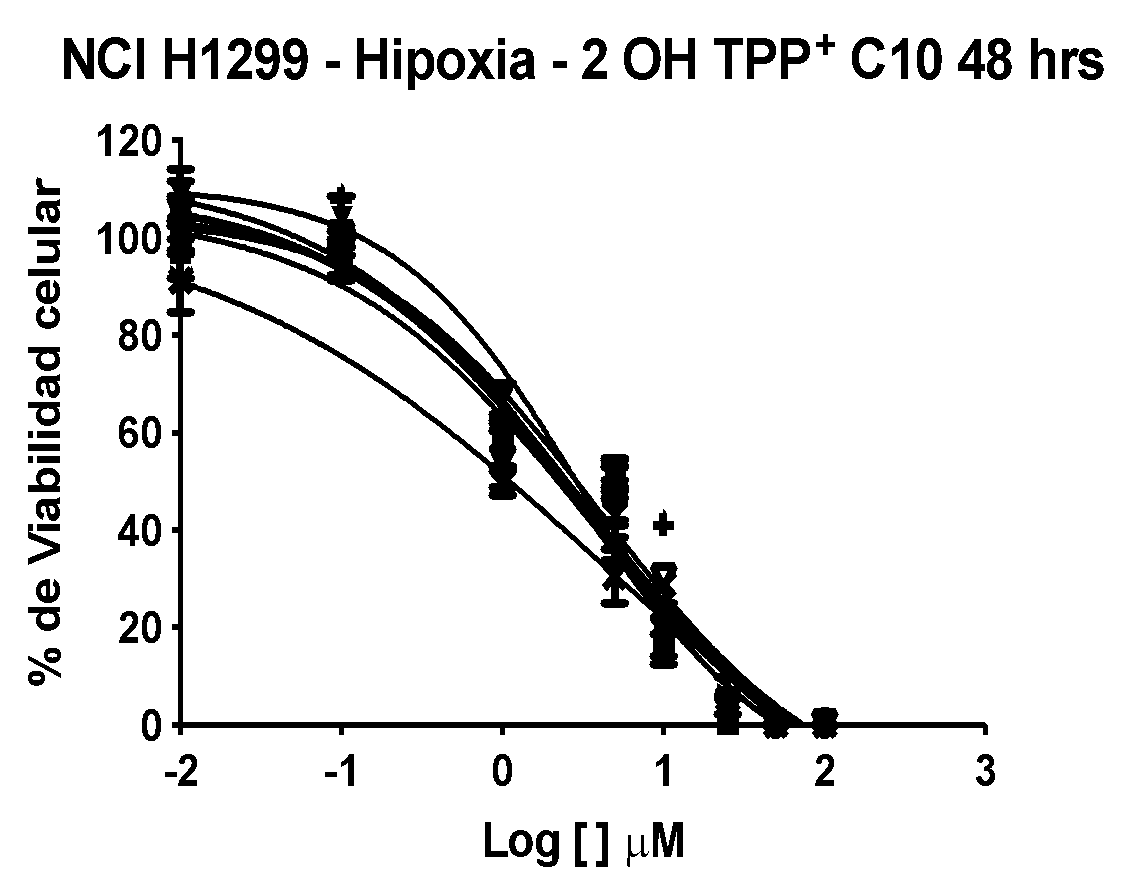**  **E** | **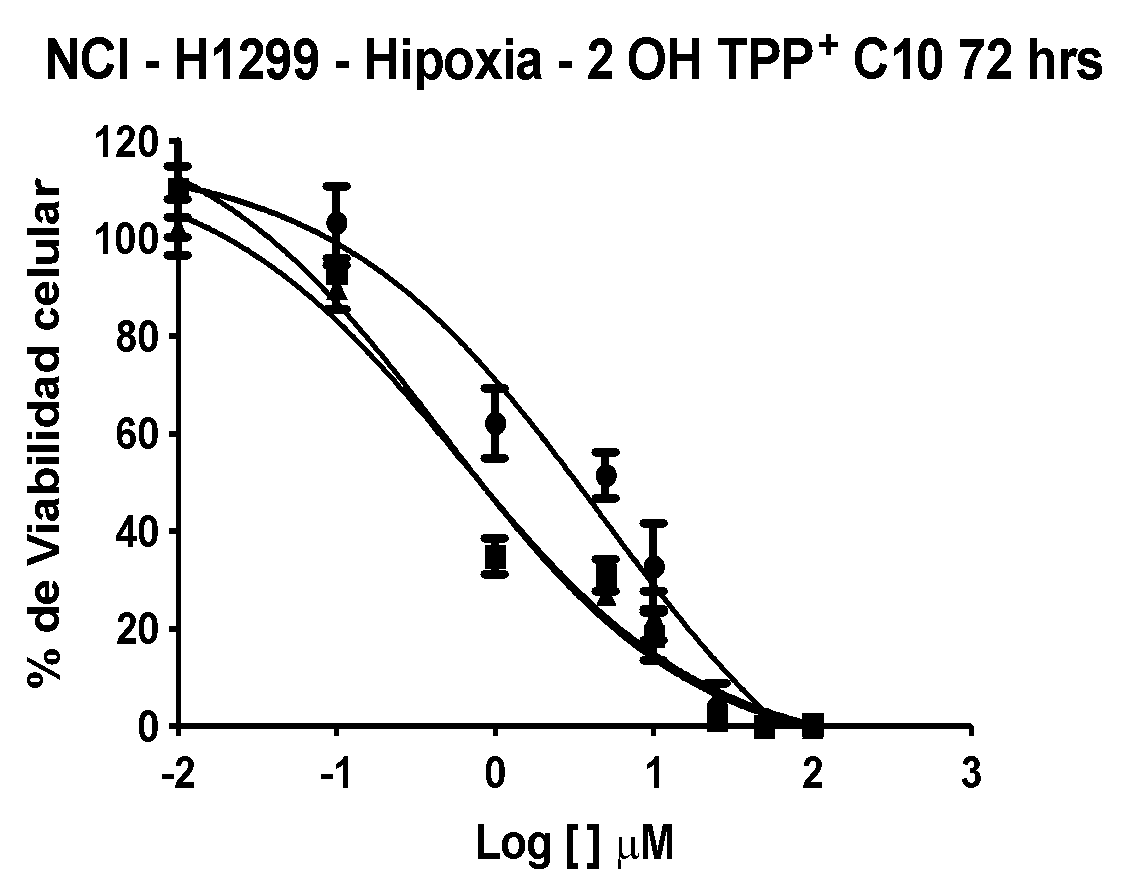**  **F** |
| **IC_50_** | 7,93 ± 0,93 *μM* | 3,33 ± 0,34 *μM* | 0,54 ± 0,09 *μM* |

**Figure S14.** Cytotoxic effect of SA-TPP+C10 on NCI H1299 cell line monolayer cultures under normoxic and hypoxic conditions. Graphical representation of the effect of the compound SA-TPP+C10 on cell viability as a function of the logarithm of the concentration. The cytotoxic effect is observed at 24 h (A and D), 48 h (B and E) and 72 h (C and F). Data obtained by averaging at least 3 independent experiments. The averages of the IC_50_ ± SD are shown.

**Lung fibroblast**

|  | **24 h** | **48 h** | **72 h** |
| --- | --- | --- | --- |
| **Normoxic** | **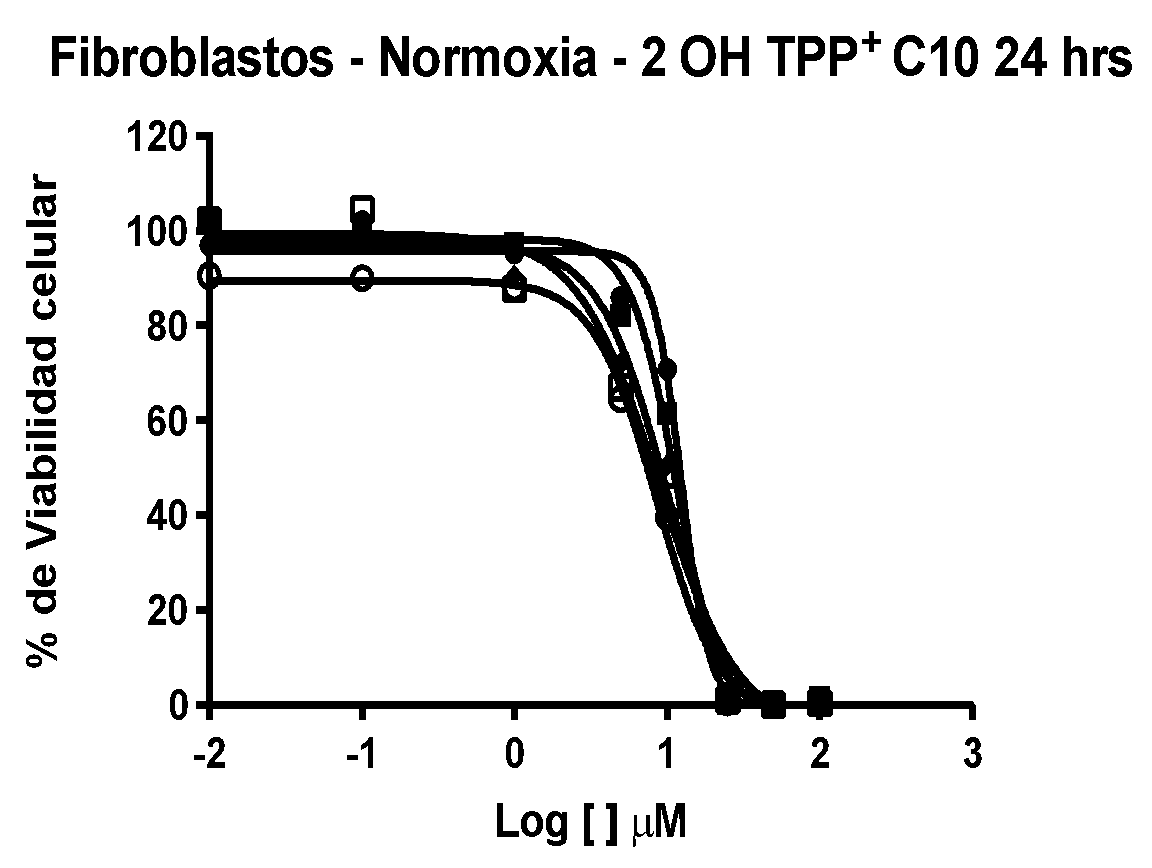**  **A** | **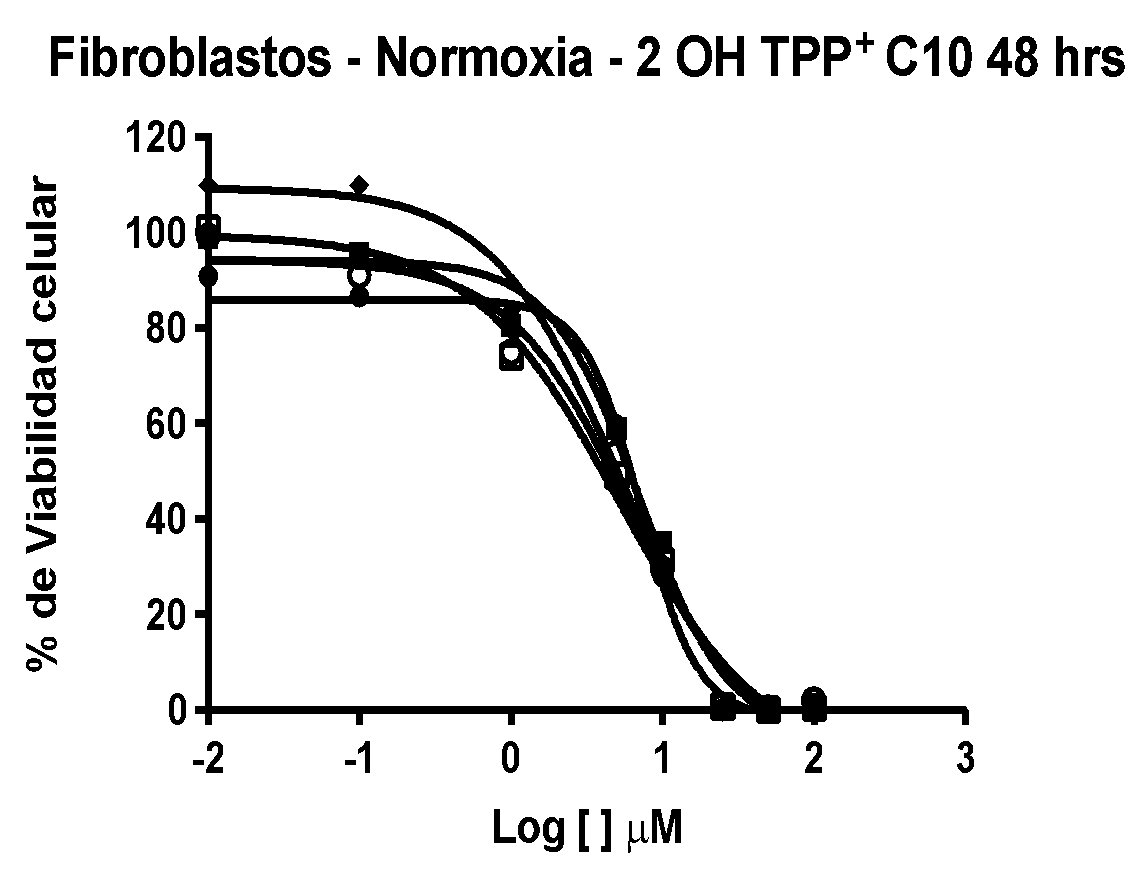**  **B** | **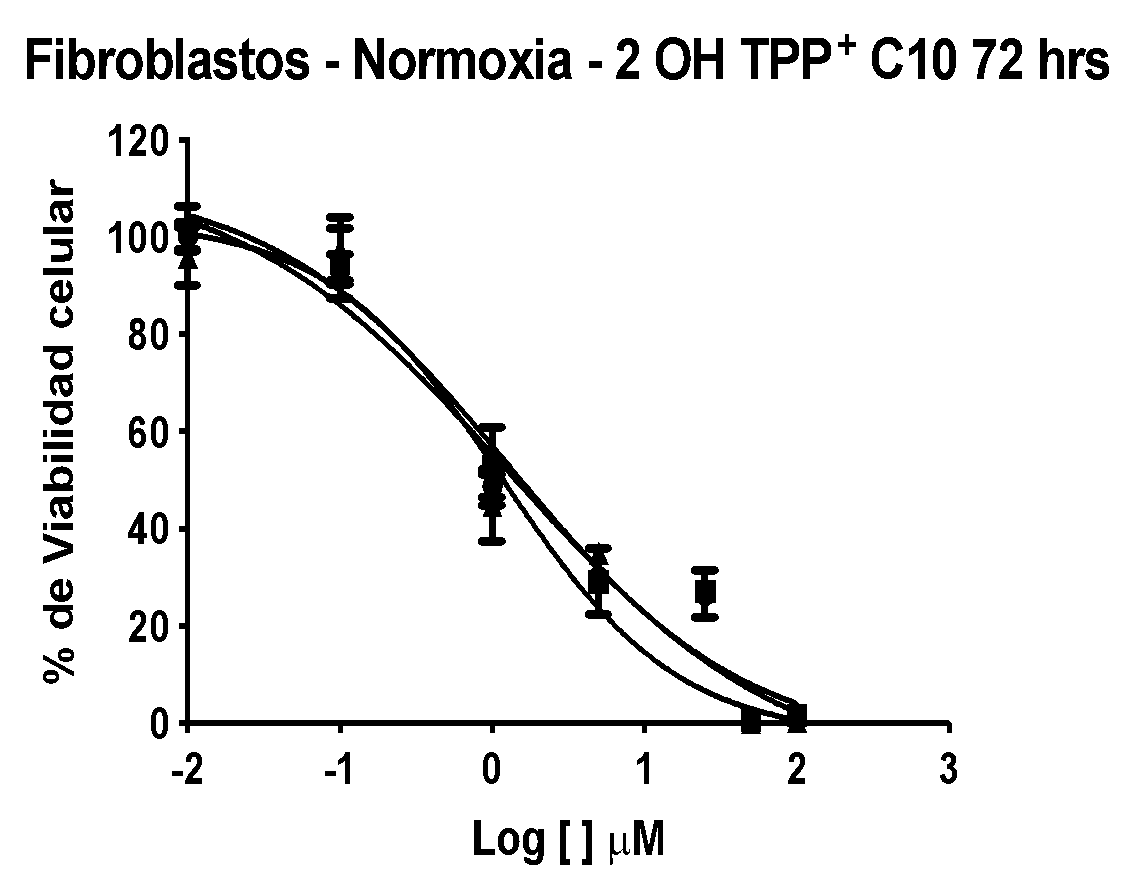**  **C** |
| **IC_50_** | 10,14 ± 1,65 *μM* | 6,09 ± 1,04 *μM* | 1,26 ± 0,08 *μM* |

**Figure S15.** Cytotoxic effect of SA-TPP^+^C_10_ on lung fibroblast cell line monolayer cultures under normoxic conditions. Graphical representation of the effect of the compound SA-TPP^+^C_10_ on cell viability as a function of the logarithm of the concentration. The cytotoxic effect is observed at 24 h (A), 48 h (B), and 72 h (C). Data obtained by averaging at least 3 independent experiments. The averages of the IC_50_ ± SD are shown.
